# Posicionamento sobre Diagnóstico e Tratamento da Amiloidose Cardíaca – 2021

**DOI:** 10.36660/abc.20210718

**Published:** 2021-09-01

**Authors:** Marcus V. Simões, Fabio Fernandes, Fabiana G. Marcondes-Braga, Philip Scheinberg, Edileide de Barros Correia, Luis Eduardo P. Rohde, Fernando Bacal, Silvia Marinho Martins Alves, Sandrigo Mangini, Andréia Biolo, Luis Beck-da-Silva, Roberta Shcolnik Szor, Wilson Marques, Acary Souza Bulle Oliveira, Márcia Waddington Cruz, Bruno Vaz Kerges Bueno, Ludhmila Abrahão Hajjar, Aurora Felice Castro Issa, Felix José Alvarez Ramires, Otavio Rizzi Coelho, André Schmidt, Ibraim Masciarelli Francisco Pinto, Carlos Eduardo Rochitte, Marcelo Luiz Campos Vieira, Cláudio Tinoco Mesquita, Celso Dario Ramos, José Soares-Junior, Minna Moreira Dias Romano, Wilson Mathias, Marcelo Iório Garcia, Marcelo Westerlund Montera, Marcelo Dantas Tavares de Melo, Sandra Marques e Silva, Pedro Manoel Marques Garibaldi, Aristóteles Comte de Alencar, Renato Delascio Lopes, Diane Xavier de Ávila, Denizar Viana, José Francisco Kerr Saraiva, Manoel Fernandes Canesin, Glaucia Maria Moraes de Oliveira, Evandro Tinoco Mesquita

**Affiliations:** 1 Universidade de São Paulo Faculdade de Medicina de Ribeirão Preto Ribeirão Preto Brasil Faculdade de Medicina de Ribeirão Preto da Universidade de São Paulo, Ribeirão Preto – Brasil; 2 Universidade de São Paulo Hospital das Clínicas da Faculdade de Medicina Instituto do Coração São Paulo SP Brasil Instituto do Coração (InCor) do Hospital das Clínicas da Faculdade de Medicina da Universidade de São Paulo (HCFMUSP), São Paulo, SP – Brasil; 3 Hospital da Beneficência Portuguesa de São Paulo São Paulo SP Brasil Hospital da Beneficência Portuguesa de São Paulo, São Paulo, SP – Brasil; 4 Instituto Dante Pazzanese de Cardiologia São Paulo SP Brasil Instituto Dante Pazzanese de Cardiologia, São Paulo, SP – Brasil; 5 Hospital de Clínicas de Porto Alegre Porto Alegre RS Brasil Hospital de Clínicas de Porto Alegre, Porto Alegre, RS – Brasil; 6 Hospital Moinhos de Vento Porto Alegre RS Brasil Hospital Moinhos de Vento, Porto Alegre, RS – Brasil; 7 Universidade Federal do Rio Grande do Sul Porto Alegre RS Brasil Universidade Federal do Rio Grande do Sul (UFRGS), Porto Alegre, RS – Brasil; 8 Pronto Socorro Cardiológico de Pernambuco Recife PE Brasil Pronto Socorro Cardiológico de Pernambuco (PROCAPE), Recife, PE – Brasil; 9 Universidade de Pernambuco Recife PE Brasil Universidade de Pernambuco (UPE), Recife, PE – Brasil; 10 Fundação Faculdade de Medicina São Paulo SP Brasil Fundação Faculdade de Medicina, São Paulo, SP – Brasil; 11 Universidade de São Paulo São Paulo SP Brasil Instituto do Câncer do Estado de São Paulo da Faculdade de Medicina da Universidade de São Paulo, São Paulo, SP – Brasil; 12 Universidade Federal de São Paulo São Paulo SP Brasil Universidade Federal de São Paulo, São Paulo, SP – Brasil; 13 Universidade Federal do Rio de Janeiro Rio de Janeiro RJ Brasil Hospital Universitário Clementino Fraga Filho (HUCFF) da Universidade Federal do Rio de Janeiro (UFRJ), Rio de Janeiro, RJ – Brasil; 14 Faculdade de Ciências Médicas da Santa Casa de São Paulo São Paulo SP Brasil Faculdade de Ciências Médicas da Santa Casa de São Paulo, São Paulo, SP – Brasil; 15 Instituto Nacional de Cardiologia Rio de Janeiro RJ Brasil Instituto Nacional de Cardiologia, Rio de Janeiro, RJ – Brasil; 16 Hospital Israelita Albert Einstein São Paulo SP Brasil Hospital Israelita Albert Einstein, São Paulo, SP – Brasil; 17 Universidade Estadual de Campinas Campinas SP Brasil Faculdade de Ciências Médicas da Universidade Estadual de Campinas (UNICAMP), Campinas, SP – Brasil; 18 Grupo Fleury São Paulo SP Brasil Grupo Fleury, São Paulo, SP – Brasil; 19 Hospital do Coração São Paulo SP Brasil Hospital do Coração (HCor), São Paulo, SP – Brasil; 20 Hospital Pró-Cardíaco Rio de Janeiro RJ Brasil Hospital Pró-Cardíaco, Rio de Janeiro, RJ – Brasil; 21 Universidade Federal Fluminense Rio de Janeiro RJ Brasil Universidade Federal Fluminense (UFF), Rio de Janeiro, RJ – Brasil; 22 Universidade Federal da Paraíba João Pessoa PB Brasil Universidade Federal da Paraíba (UFPB), João Pessoa, PB – Brasil; 23 Hospital de Base do DF Brasília DF Brasil Hospital de Base do DF, Brasília, DF – Brasil; 24 Duke University Durham EUA Duke University, Durham – EUA; 25 Complexo Hospitalar de Niterói Rio de Janeiro RJ Brasil Complexo Hospitalar de Niterói, Rio de Janeiro, RJ – Brasil; 26 Hospital e Maternidade Christóvão da Gama Santo André SP Brasil Hospital e Maternidade Christóvão da Gama, Santo André, SP – Brasil; 27 Hospital Universitário Antônio Pedro Rio de Janeiro RJ Brasil Hospital Universitário Antônio Pedro (Huap), Rio de Janeiro, RJ – Brasil; 28 Universidade do Estado do Rio de Janeiro Rio de Janeiro RJ Brasil Universidade do Estado do Rio de Janeiro, Rio de Janeiro, RJ – Brasil; 29 Sociedade Campineira de Educação e Instrução Campinas SP Brasil Sociedade Campineira de Educação e Instrução, Campinas, SP – Brasil; 30 Universidade Estadual de Londrina Londrina PR Brasil Hospital Universitário da Universidade Estadual de Londrina, Londrina, PR – Brasil; 31 Universidade Federal do Rio de Janeiro Rio de Janeiro RJ Brasil Universidade Federal do Rio de Janeiro (UFRJ), Rio de Janeiro, RJ – Brasil; 32 Centro de Ensino e Treinamento Edson de Godoy Bueno Rio de Janeiro RJ Brasil Centro de Ensino e Treinamento Edson de Godoy Bueno/UHG, Rio de Janeiro, RJ – Brasil

**Table:** 

Posicionamento sobre Diagnóstico e Tratamento da Amiloidose Cardíaca – 2021	
O relatório abaixo lista as declarações de interesse conforme relatadas à SBC pelos especialistas durante o período de desenvolvimento deste posicionamento, 2020/2021.	
Especialista	Tipo de relacionamento com a indústria
Acary Souza Bulle Oliveira	Nada a ser declarado
André Schmidt	Declaração financeira
	A - Pagamento de qualquer espécie e desde que economicamente apreciáveis, feitos a (i) você, (ii) ao seu cônjuge/ companheiro ou a qualquer outro membro que resida com você, (iii) a qualquer pessoa jurídica em que qualquer destes seja controlador, sócio, acionista ou participante, de forma direta ou indireta, recebimento por palestras, aulas, atuação como proctor de treinamentos, remunerações, honorários pagos por participações em conselhos consultivos, de investigadores, ou outros comitês, etc. Provenientes da indústria farmacêutica, de órteses, próteses, equipamentos e implantes, brasileiras ou estrangeiras:
	- Bayer: Anticoagulantes.
	Outros relacionamentos
	Financiamento de atividades de educação médica continuada, incluindo viagens, hospedagens e inscrições para congressos e cursos, provenientes da indústria farmacêutica, de órteses, próteses, equipamentos e implantes, brasileiras ou estrangeiras:
	- Sanofi: Anticoagulantes.
Andréia Biolo	Nada a ser declarado
Aristóteles Comte de Alencar Neto	Nada a ser declarado
Aurora Felice Castro Issa	Nada a ser declarado
Bruno Vaz Kerges Bueno	Declaração financeira
	A - Pagamento de qualquer espécie e desde que economicamente apreciáveis, feitos a (i) você, (ii) ao seu cônjuge/ companheiro ou a qualquer outro membro que resida com você, (iii) a qualquer pessoa jurídica em que qualquer destes seja controlador, sócio, acionista ou participante, de forma direta ou indireta, recebimento por palestras, aulas, atuação como proctor de treinamentos, remunerações, honorários pagos por participações em conselhos consultivos, de investigadores, ou outros comitês, etc. Provenientes da indústria farmacêutica, de órteses, próteses, equipamentos e implantes, brasileiras ou estrangeiras:
	- Pfiser: Eliquis; Alnylam: Patisiran.
Carlos Eduardo Rochitte	Nada a ser declarado
Celso Dario Ramos	Nada a ser declarado
Cláudio Tinoco Mesquita	Declaração financeira
	A - Pagamento de qualquer espécie e desde que economicamente apreciáveis, feitos a (i) você, (ii) ao seu cônjuge/ companheiro ou a qualquer outro membro que resida com você, (iii) a qualquer pessoa jurídica em que qualquer destes seja controlador, sócio, acionista ou participante, de forma direta ou indireta, recebimento por palestras, aulas, atuação como proctor de treinamentos, remunerações, honorários pagos por participações em conselhos consultivos, de investigadores, ou outros comitês, etc. Provenientes da indústria farmacêutica, de órteses, próteses, equipamentos e implantes, brasileiras ou estrangeiras:
	- Pfizer: Participação em advisory board de amiloidose.
	B - Financiamento de pesquisas sob sua responsabilidade direta/pessoal (direcionado ao departamento ou instituição) provenientes da indústria farmacêutica, de órteses, próteses, equipamentos e implantes, brasileiras ou estrangeiras:
	- Alnylam: Investigador Principal do estudo Apollo B com Patisiran.
	Outros relacionamentos
	Financiamento de atividades de educação médica continuada, incluindo viagens, hospedagens e inscrições para congressos e cursos, provenientes da indústria farmacêutica, de órteses, próteses, equipamentos e implantes, brasileiras ou estrangeiras:
	- Pfizer: Amiloidose.
Denizar Viana	Nada a ser declarado
Diane Xavier de Ávila	Declaração financeira
	A - Pagamento de qualquer espécie e desde que economicamente apreciáveis, feitos a (i) você, (ii) ao seu cônjuge/ companheiro ou a qualquer outro membro que resida com você, (iii) a qualquer pessoa jurídica em que qualquer destes seja controlador, sócio, acionista ou participante, de forma direta ou indireta, recebimento por palestras, aulas, atuação como proctor de treinamentos, remunerações, honorários pagos por participações em conselhos consultivos, de investigadores, ou outros comitês, etc. Provenientes da indústria farmacêutica, de órteses, próteses, equipamentos e implantes, brasileiras ou estrangeiras:
	- Alnylam: Patisiran.
	Outros relacionamentos
	Financiamento de atividades de educação médica continuada, incluindo viagens, hospedagens e inscrições para congressos e cursos, provenientes da indústria farmacêutica, de órteses, próteses, equipamentos e implantes, brasileiras ou estrangeiras:
	- Alnylam: Patisiran.
Edileide de Barros Correia	Declaração financeira
	A - Pagamento de qualquer espécie e desde que economicamente apreciáveis, feitos a (i) você, (ii) ao seu cônjuge/ companheiro ou a qualquer outro membro que resida com você, (iii) a qualquer pessoa jurídica em que qualquer destes seja controlador, sócio, acionista ou participante, de forma direta ou indireta, recebimento por palestras, aulas, atuação como proctor de treinamentos, remunerações, honorários pagos por participações em conselhos consultivos, de investigadores, ou outros comitês, etc. Provenientes da indústria farmacêutica, de órteses, próteses, equipamentos e implantes, brasileiras ou estrangeiras:
	- Pfizer: Tafamidis (Vyndaqel); Alnylam: Patsiram (Onpatro); Takeda: Replagal.
Evandro Tinoco Mesquita	Vínculo empregatício com a indústria farmacêutica, de órteses, próteses, Equipamentos e implantes, brasileiras ou estrangeiras, assim como se tem Relação vínculo empregatício com operadoras de planos de saúde ou em Auditorias médicas (incluindo meio período) durante o ano para o qual você está declarando:
	- UnitedHealth Group.
Fabiana G. Marcondes-Braga	Declaração financeira
	A - Pagamento de qualquer espécie e desde que economicamente apreciáveis, feitos a (i) você, (ii) ao seu cônjuge/ companheiro ou a qualquer outro membro que resida com você, (iii) a qualquer pessoa jurídica em que qualquer destes seja controlador, sócio, acionista ou participante, de forma direta ou indireta, recebimento por palestras, aulas, atuação como proctor de treinamentos, remunerações, honorários pagos por participações em conselhos consultivos, de investigadores, ou outros comitês, etc. Provenientes da indústria farmacêutica, de órteses, próteses, equipamentos e implantes, brasileiras ou estrangeiras:
	- Novartis: Palestras; AstraZeneca: Palestras e Conselho Consultivo; Boehringer: Conselho Consultivo.
Fabio Fernandes	Declaração financeira
	A - Pagamento de qualquer espécie e desde que economicamente apreciáveis, feitos a (i) você, (ii) ao seu cônjuge/ companheiro ou a qualquer outro membro que resida com você, (iii) a qualquer pessoa jurídica em que qualquer destes seja controlador, sócio, acionista ou participante, de forma direta ou indireta, recebimento por palestras, aulas, atuação como proctor de treinamentos, remunerações, honorários pagos por participações em conselhos consultivos, de investigadores, ou outros comitês, etc. Provenientes da indústria farmacêutica, de órteses, próteses, equipamentos e implantes, brasileiras ou estrangeiras:
	- Pfiser, Alnylan: palestras.
	Outros relacionamentos
	Financiamento de atividades de educação médica continuada, incluindo viagens, hospedagens e inscrições para congressos e cursos, provenientes da indústria farmacêutica, de órteses, próteses, equipamentos e implantes, brasileiras ou estrangeiras:
	- Phifesr: Participação em congresso internacional.
Felix José Alvarez Ramires	Declaração financeira
	A - Pagamento de qualquer espécie e desde que economicamente apreciáveis, feitos a (i) você, (ii) ao seu cônjuge/companheiro ou a qualquer outro membro que resida com você, (iii) a qualquer pessoa jurídica em que qualquer destes seja controlador, sócio, acionista ou participante, de forma direta ou indireta, recebimento por palestras, aulas, atuação como proctor de treinamentos, remunerações, honorários pagos por participações em conselhos consultivos, de investigadores, ou outros comitês, etc. Provenientes da indústria farmacêutica, de órteses, próteses, equipamentos e implantes, brasileiras ou estrangeiras:
	- Novartis: Sacubitril/Valsartana; Pfizer: Patisiran; Merck: Vericiquat; Amgen.
Fernando Bacal	Nada a ser declarado
Glaucia Maria Moraes de Oliveira	Nada a ser declarado
Ibraim Masciarelli Francisco Pinto	Declaração financeira
	A - Pagamento de qualquer espécie e desde que economicamente apreciáveis, feitos a (i) você, (ii) ao seu cônjuge/companheiro ou a qualquer outro membro que resida com você, (iii) a qualquer pessoa jurídica em que qualquer destes seja controlador, sócio, acionista ou participante, de forma direta ou indireta, recebimento por palestras, aulas, atuação como proctor de treinamentos, remunerações, honorários pagos por participações em conselhos consultivos, de investigadores, ou outros comitês, etc. Provenientes da indústria farmacêutica, de órteses, próteses, equipamentos e implantes, brasileiras ou estrangeiras:
	- Novo Nordisk: Diabetes
José Francisco Kerr Saraiva	Nada a ser declarado
José Soares-Junior	Nada a ser declarado
Ludhmila Abrahão Hajjar	Nada a ser declarado
Luis Beck-da-Silva	Declaração financeira
	A - Pagamento de qualquer espécie e desde que economicamente apreciáveis, feitos a (i) você, (ii) ao seu cônjuge/companheiro ou a qualquer outro membro que resida com você, (iii) a qualquer pessoa jurídica em que qualquer destes seja controlador, sócio, acionista ou participante, de forma direta ou indireta, recebimento por palestras, aulas, atuação como proctor de treinamentos, remunerações, honorários pagos por participações em conselhos consultivos, de investigadores, ou outros comitês, etc. Provenientes da indústria farmacêutica, de órteses, próteses, equipamentos e implantes, brasileiras ou estrangeiras:
	- Novartis: Insuficiência Cardíaca; AstraZeneca: Insuficiência Cardíaca.
	B - Financiamento de pesquisas sob sua responsabilidade direta/pessoal (direcionado ao departamento ou instituição) provenientes da indústria farmacêutica, de órteses, próteses, equipamentos e implantes, brasileiras ou estrangeiras:
	- Amgen: Insuficiência Cardíaca.
Luis Eduardo P. Rohde	Declaração financeira
	A - Pagamento de qualquer espécie e desde que economicamente apreciáveis, feitos a (i) você, (ii) ao seu cônjuge/ companheiro ou a qualquer outro membro que resida com você, (iii) a qualquer pessoa jurídica em que qualquer destes seja controlador, sócio, acionista ou participante, de forma direta ou indireta, recebimento por palestras, aulas, atuação como proctor de treinamentos, remunerações, honorários pagos por participações em conselhos consultivos, de investigadores, ou outros comitês, etc. Provenientes da indústria farmacêutica, de órteses, próteses, equipamentos e implantes, brasileiras ou estrangeiras:
	- Pfizer: Tafamidis; Novartir: Sacubitril-Valsartan; AstraZeneca: Dpaglifozina; Boehringer Ingelheim: Empaglifozina; Merck: Bisoprolol; Amgen: Omecamtiv-Mercabil.
	Outros relacionamentos
	Financiamento de atividades de educação médica continuada, incluindo viagens, hospedagens e inscrições para congressos e cursos, provenientes da indústria farmacêutica, de órteses, próteses, equipamentos e implantes, brasileiras ou estrangeiras:
	- AstraZeneca: Dapaglifozina; Boehringer Ingelheim: Empaglifozina.
Manoel Fernandes Canesin	Nada a ser declarado
Marcelo Dantas Tavares de Melo	Nada a ser declarado
Marcelo Iório Garcia	Nada a ser declarado
Marcelo Luiz Campos Vieira	Nada a ser declarado
Marcelo Westerlund Montera	Nada a ser declarado
Márcia Waddington Cruz	Declaração financeira
	A - Pagamento de qualquer espécie e desde que economicamente apreciáveis, feitos a (i) você, (ii) ao seu cônjuge/ companheiro ou a qualquer outro membro que resida com você, (iii) a qualquer pessoa jurídica em que qualquer destes seja controlador, sócio, acionista ou participante, de forma direta ou indireta, recebimento por palestras, aulas, atuação como proctor de treinamentos, remunerações, honorários pagos por participações em conselhos consultivos, de investigadores, ou outros comitês, etc. Provenientes da indústria farmacêutica, de órteses, próteses, equipamentos e implantes, brasileiras ou estrangeiras:
	- Pfizer, Alnylam, Genzyme, Ionis, Prothena, FoldRx, PTC, NIH: Ppresentação em evento científico como investigadora principal de ensaios clínicos e como consultoria.
	B - Financiamento de pesquisas sob sua responsabilidade direta/pessoal (direcionado ao departamento ou instituição) provenientes da indústria farmacêutica, de órteses, próteses, equipamentos e implantes, brasileiras ou estrangeiras:
	- Pfizer, Alnylam, Genzyme, Ionis, Prothena, FoldRx, PTC, NIH: Apresentação em evento científico como investigadora principal de ensaios clínicos e como consultoria.
	Outros relacionamentos
	Financiamento de atividades de educação médica continuada, incluindo viagens, hospedagens e inscrições para congressos e cursos, provenientes da indústria farmacêutica, de órteses, próteses, equipamentos e implantes, brasileiras ou estrangeiras:
	- Pfizer, Alnylam, Genzyme, Ionis, Prothena, FoldRx, PTC, NIH: Apresentação em evento científico como investigadora principal de ensaios clínicos e como consultoria.
Marcus V. Simões	Declaração financeira
	A - Pagamento de qualquer espécie e desde que economicamente apreciáveis, feitos a (i) você, (ii) ao seu cônjuge/companheiro ou a qualquer outro membro que resida com você, (iii) a qualquer pessoa jurídica em que qualquer destes seja controlador, sócio, acionista ou participante, de forma direta ou indireta, recebimento por palestras, aulas, atuação como proctor de treinamentos, remunerações, honorários pagos por participações em conselhos consultivos, de investigadores, ou outros comitês, etc. Provenientes da indústria farmacêutica, de órteses, próteses, equipamentos e implantes, brasileiras ou estrangeiras:
	- Novartis: Entresto; AstraZeneca: Dapagliflozina.
	B - Financiamento de pesquisas sob sua responsabilidade direta/pessoal (direcionado ao departamento ou instituição) provenientes da indústria farmacêutica, de órteses, próteses, equipamentos e implantes, brasileiras ou estrangeiras:
- Amgen: Omecamtiv/Mecarbil; Beringher Ingelheim: Empagliflozina.
Minna Moreira Dias Romano	Nada a ser declarado
Otavio Rizzi Coelho Filho	Declaração financeira
	A - Pagamento de qualquer espécie e desde que economicamente apreciáveis, feitos a (i) você, (ii) ao seu cônjuge/ companheiro ou a qualquer outro membro que resida com você, (iii) a qualquer pessoa jurídica em que qualquer destes seja controlador, sócio, acionista ou participante, de forma direta ou indireta, recebimento por palestras, aulas, atuação como proctor de treinamentos, remunerações, honorários pagos por participações em conselhos consultivos, de investigadores, ou outros comitês, etc. Provenientes da indústria farmacêutica, de órteses, próteses, equipamentos e implantes, brasileiras ou estrangeiras:
	- Pfizer: Tratamento para amiloidose TTR; Takeda: Doença de Fabry; Novartis: Insuficiência cardíaca; AstraZeneca; Daiichi Sankyo; Merck; Shire; Bayer.
	B - Financiamento de pesquisas sob sua responsabilidade direta/pessoal (direcionado ao departamento ou instituição) provenientes da indústria farmacêutica, de órteses, próteses, equipamentos e implantes, brasileiras ou estrangeiras:
	- Pfizer: Tratamento para amiloidose TTR.
	Outros relacionamentos
	Financiamento de atividades de educação médica continuada, incluindo viagens, hospedagens e inscrições para congressos e cursos, provenientes da indústria farmacêutica, de órteses, próteses, equipamentos e implantes, brasileiras ou estrangeiras:
	- AstraZeneca: Insuficiência cardíaca.
Pedro Manoel Marques Garibaldi	Nada a ser declarado
Philip Scheinberg	Declaração financeira
	A - Pagamento de qualquer espécie e desde que economicamente apreciáveis, feitos a (i) você, (ii) ao seu cônjuge/ companheiro ou a qualquer outro membro que resida com você, (iii) a qualquer pessoa jurídica em que qualquer destes seja controlador, sócio, acionista ou participante, de forma direta ou indireta, recebimento por palestras, aulas, atuação como proctor de treinamentos, remunerações, honorários pagos por participações em conselhos consultivos, de investigadores, ou outros comitês, etc. Provenientes da indústria farmacêutica, de órteses, próteses, equipamentos e implantes, brasileiras ou estrangeiras:
	- Novartis, Abbvie, Roche: Hematologia.
Renato Delascio Lopes	Declaração financeira
	A - Pagamento de qualquer espécie e desde que economicamente apreciáveis, feitos a (i) você, (ii) ao seu cônjuge/ companheiro ou a qualquer outro membro que resida com você, (iii) a qualquer pessoa jurídica em que qualquer destes seja controlador, sócio, acionista ou participante, de forma direta ou indireta, recebimento por palestras, aulas, atuação como proctor de treinamentos, remunerações, honorários pagos por participações em conselhos consultivos, de investigadores, ou outros comitês, etc. Provenientes da indústria farmacêutica, de órteses, próteses, equipamentos e implantes, brasileiras ou estrangeiras:
	- Bayer: Anticoagulante; Boehringer Ingleheim: Anticoagulação e Diabetes; Pfizer: Anticoagulação; Bristol-Myers Squibb; Daiichi Sankyo; Glaxo Smith Kline; Medtronic; Merck; Portola; Sanofi.
	B - financiamento de pesquisas sob sua responsabilidade direta/pessoal (direcionado ao departamento ou instituição) provenientes da indústria farmacêutica, de órteses, próteses, equipamentos e implantes, brasileiras ou estrangeiras:
	- Pfizer: Apixaban; Bayer: Rivaroxaban; Novartis: Sacubitril, Valsartan.
	Outros relacionamentos
	Financiamento de atividades de educação médica continuada, incluindo viagens, hospedagens e inscrições para congressos e cursos, provenientes da indústria farmacêutica, de órteses, próteses, equipamentos e implantes, brasileiras ou estrangeiras:
	- Bayer: Rivaroxabana; Pfizer: Apixabana
Roberta Shcolnik Szor	Declaração financeira
	A - Pagamento de qualquer espécie e desde que economicamente apreciáveis, feitos a (i) você, (ii) ao seu cônjuge/ companheiro ou a qualquer outro membro que resida com você, (iii) a qualquer pessoa jurídica em que qualquer destes seja controlador, sócio, acionista ou participante, de forma direta ou indireta, recebimento por palestras, aulas, atuação como proctor de treinamentos, remunerações, honorários pagos por participações em conselhos consultivos, de investigadores, ou outros comitês, etc. Provenientes da indústria farmacêutica, de órteses, próteses, equipamentos e implantes, brasileiras ou estrangeiras:
	- Pfizer: Amiloidose.
	B - Financiamento de pesquisas sob sua responsabilidade direta/pessoal (direcionado ao departamento ou instituição) provenientes da indústria farmacêutica, de órteses, próteses, equipamentos e implantes, brasileiras ou estrangeiras:
	- Janssen-Cilag Farmacêutica Ltda.: Amiloidose de cadeia leve.
	Outros relacionamentos
	Financiamento de atividades de educação médica continuada, incluindo viagens, hospedagens e inscrições para congressos e cursos, provenientes da indústria farmacêutica, de órteses, próteses, equipamentos e implantes, brasileiras ou estrangeiras:
	- Janssen-Cilag Farmacêutica Ltda.: Amiloidose de cadeia leve.
Sandra Marques e Silva	Declaração financeira
	A - Pagamento de qualquer espécie e desde que economicamente apreciáveis, feitos a (i) você, (ii) ao seu cônjuge/ companheiro ou a qualquer outro membro que resida com você, (iii) a qualquer pessoa jurídica em que qualquer destes seja controlador, sócio, acionista ou participante, de forma direta ou indireta, recebimento por palestras, aulas, atuação como proctor de treinamentos, remunerações, honorários pagos por participações em conselhos consultivos, de investigadores, ou outros comitês, etc. Provenientes da indústria farmacêutica, de órteses, próteses, equipamentos e implantes, brasileiras ou estrangeiras:
	- Sanofi, Takeda, Amicus Therapeutics: Doenças raras; Pfiser.
	Outros relacionamentos
	Financiamento de atividades de educação médica continuada, incluindo viagens, hospedagens e inscrições para congressos e cursos, provenientes da indústria farmacêutica, de órteses, próteses, equipamentos e implantes, brasileiras ou estrangeiras:
	- Sanofi, Takeda, Amicus Therapeutics: Doenças raras; Pfiser.
Sandrigo Mangini	Declaração financeira
	A - Pagamento de qualquer espécie e desde que economicamente apreciáveis, feitos a (i) você, (ii) ao seu cônjuge/companheiro ou a qualquer outro membro que resida com você, (iii) a qualquer pessoa jurídica em que qualquer destes seja controlador, sócio, acionista ou participante, de forma direta ou indireta, recebimento por palestras, aulas, atuação como proctor de treinamentos, remunerações, honorários pagos por participações em conselhos consultivos, de investigadores, ou outros comitês, etc. Provenientes da indústria farmacêutica, de órteses, próteses, equipamentos e implantes, brasileiras ou estrangeiras:
	- Novartis: Sacubitril/Valsartan; Pfizer: Doenças raras.
	Outros relacionamentos
	Financiamento de atividades de educação médica continuada, incluindo viagens, hospedagens e inscrições para congressos e cursos, provenientes da indústria farmacêutica, de órteses, próteses, equipamentos e implantes, brasileiras ou estrangeiras:
	- Pfizer: Doenças raras
Silvia Marinho Martins Alves	Nada a ser declarado
Wilson Marques Junior	Declaração financeira
	A - Pagamento de qualquer espécie e desde que economicamente apreciáveis, feitos a (i) você, (ii) ao seu cônjuge/ companheiro ou a qualquer outro membro que resida com você, (iii) a qualquer pessoa jurídica em que qualquer destes seja controlador, sócio, acionista ou participante, de forma direta ou indireta, recebimento por palestras, aulas, atuação como proctor de treinamentos, remunerações, honorários pagos por participações em conselhos consultivos, de investigadores, ou outros comitês, etc. Provenientes da indústria farmacêutica, de órteses, próteses, equipamentos e implantes, brasileiras ou estrangeiras:
	- Pfizer: Tafamidis; Alnylam: Patisiran; PTC: Inotersen.
	B - Financiamento de pesquisas sob sua responsabilidade direta/pessoal (direcionado ao departamento ou instituição) provenientes da indústria farmacêutica, de órteses, próteses, equipamentos e implantes, brasileiras ou estrangeiras:
	- Pfizer: Amiloidose.
	Outros relacionamentos
	Financiamento de atividades de educação médica continuada, incluindo viagens, hospedagens e inscrições para congressos e cursos, provenientes da indústria farmacêutica, de órteses, próteses, equipamentos e implantes, brasileiras ou estrangeiras:
	- Pfizer: Tafamidis; Alnylam: Patisiran; PTC: Inotersen.
Wilson Mathias Junior	Nada a ser declarado

## 1. Introdução

Nos últimos anos, foram consolidados avanços expressivos no conhecimento de amiloidose cardíaca (AC), trazendo uma profunda reformulação do seu significado clínico. Além de haver evidências convincentes de que AC seja uma causa relativamente comum de insuficiência cardíaca com fração de ejeção preservada (ICFEP), assistimos o surgimento de terapias específicas modificadoras do curso natural da doença, capazes de prolongar a sobrevida dos pacientes acometidos.

Em paralelo, relevantes progressos nas técnicas de imagem cardiovascular têm contribuído grandemente para o reconhecimento mais acurado e precoce da doença. Particularmente, o emprego da cintilografia cardíaca com radiotraçadores ósseos tornou possível o diagnóstico não invasivo da AC ligada à transtirretina (TTR), prescindindo da biópsia endomiocárdica, o que simplificou muito o fluxo diagnóstico.

Assim, o presente posicionamento tem por objetivo apresentar as recomendações mais atuais para o diagnóstico, estadiamento prognóstico e tratamento da AC, com base na revisão crítica das evidências científicas atuais.

Neste posicionamento, as tabelas de classes de recomendação e níveis de evidência foram realizadas conforme a padronização a seguir.

**Table:** 

Classes (graus) de recomendação:
**Classe I –** Condições para as quais há evidências conclusivas ou, em sua ausência, consenso geral de que o procedimento é seguro e útil/eficaz
**Classe IIa –** Condições para as quais há evidências conflitantes e/ou divergência de opinião sobre segurança e utilidade/eficácia do procedimento. Peso ou evidência/opinião a favor do procedimento. Amaioria dos estudos/especialistas aprova
**Classe IIb –** Condições para as quais há evidências conflitantes e/ou divergência de opinião sobre segurança e utilidade/eficácia do procedimento. Segurança e utilidade/eficácia menos bem estabelecida, não havendo predomínio de opiniões a favor
**Classe III –** Condições para as quais há evidências e/ou consenso de que o procedimento não é útil/eficaz e, em alguns casos, pode ser prejudicial
**Níveis de evidência:**
**Nível A –** Dados obtidos a partir de múltiplos estudos randomizados de bom porte, concordantes e/ou de metanálise robusta de estudos clínicos randomizados
**Nível B –** Dados obtidos a partir de metanálise menos robusta, a partir de um único estudo randomizado ou de estudos não randomizados (observacionais)
**Nível C –** Dados obtidos de opiniões consensuais de especialistas

## 2. Conceitos Gerais

A amiloidose sistêmica é uma doença causada pela deposição tecidual de agregados proteicos fibrilares e insolúveis em diferentes órgãos, incluindo o coração, levando à disfunção orgânica.^1^ Mais de 30 tipos de proteínas amiloidogênicas são descritas,^2^ e cinco delas podem acometer o coração (cadeias pesada e leve da imunoglobulina, transtirretina, amiloide A e apoA1), sendo que dois tipos são responsáveis por 95% dos casos de AC: a cadeia leve de imunoglobulinas (forma AL) e a transtirretina (forma ATTR), tanto em suas formas selvagem ou *>wild type* (ATTRwt) quanto hereditária ou variante (ATTRv).^3^^–^^7^

A transtirretina (TTR) é uma proteína composta por quatro monômeros, que circulam como um tetrâmero.^8^ Age como um transportador de tiroxina (T4) e de retinol (vitamina A), em condições fisiológicas. A etapa limitante na taxa de formação das fibrilas amiloides pela TTR é a dissociação do tetrâmero em monômeros, o que possivelmente envolve proteólise. Posteriormente, a desnaturação parcial do monômetro permite a montagem incorreta em várias estruturas agregadas. A amiloidose por mutação do gene da TTR (ATTRv) apresenta caráter autossômico dominante, seu gene está localizado no cromossomo 18 e mais de 140 mutações já foram descritas. Mediante a produção de TTR menos estável, leva à deposição amiloide de forma agressiva e sistêmica.^9^

Na ATTRwt, a sequência de aminoácidos é normal e não está completamente esclarecido o processo pelo qual a proteína selvagem se torna instável e se agrega em fibrilas amiloides. No entanto, o envelhecimento parece estar envolvido na fisiopatologia da doença.^8^,^9^

Na forma AL, a cadeia leve amiloidogênica origina-se de plasmócitos ou, menos frequentemente, linfócitos B anômalos, configurando, portanto, uma doença hematológica clonal e neoplásica. No coração, o depósito de fibrilas amiloides causa dano estrutural ao aumentar a rigidez cardíaca e vascular, prejudicando a contração e o relaxamento cardíaco e gerando distúrbios de condução. Em paralelo, as cadeias leves circulantes também apresentam toxicidade direta ao miocárdio, através de disfunção lisossomal, autofagia defeituosa, produção de espécies reativas de oxigênio, disfunção celular e mitocondrial, alterações na homeostase do cálcio do cardiomiócito e, por fim, morte celular.^10^

**Figura 1:**
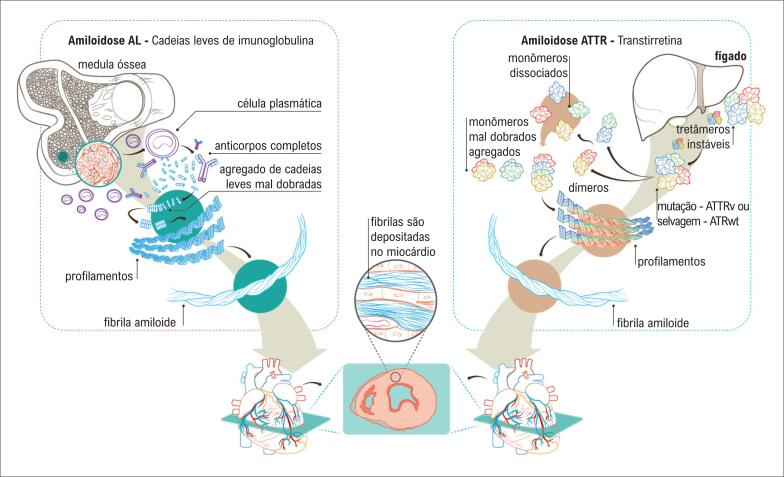
Fisiopatogênese da amiloidose cardíaca.

A Figura 1 representa a fisiopatogênese da amiloidose cardíaca nas formas ATTR e AL.

Diferentes subtipos de amiloidose podem originar manifestações clínicas sobrepostas e, uma vez diagnosticada a amiloidose, é imprescindível caracterizar corretamente a proteína precursora para instituição do tratamento específico.^11^^–^^13^

A depender dos órgãos acometidos e do grau de disfunção ocasionada, um amplo espectro de manifestações clínicas pode ser observado, com evolução progressiva e potencialmente fatal. Os principais órgãos que podem ser acometidos na amiloidose sistêmica são coração, rins, olhos, sistema nervoso central e periférico e fígado. Manifestações clínicas inespecíficas são frequentemente observadas e incluem fadiga, perda ponderal, edema periférico e hipotensão ortostática. Por esse motivo, é comum o diagnóstico tardio, de modo que são necessários conhecimento sobre a doença e elevado grau de suspeição clínica para conclusão diagnóstica.

Na ATTRv, a depender da mutação, o quadro clínico é dominado por neuropatia ou cardiopatia. Na ATTRw*t*, a cardiopatia é a principal manifestação clínica, ocorrendo principalmente em homens idosos que desenvolvem ICFEP sem fatores de risco previamente conhecidos.

Algumas alterações extracardíacas podem anteceder, emanos, o desenvolvimento da AC, destacando-se a síndrome do túnel do carpo bilateral e a ruptura espontânea do tendão do bíceps. O reconhecimento de tais sinais como parte do quadro clínico de amiloidose é fundamental, podendo levar a diagnóstico mais precoce e evitar a progressão da cardiopatia pela instituição de tratamento específico.^14^,^15^

A ATTRwt tem incidência aumentada em pacientes mais idosos, usualmente acima de 70 anos de idade; contudo, as manifestações clínicas da ATTRv também costumam ocorrer em idosos, fazendo com que a idade não deva ser levada em consideração para diferenciação entre as duas formas de ATTR. No que diz respeito ao sexo, há uma forte predominância no sexo masculino, em 80% a 90%, apenas na ATTRwt.

Com relação à ATTRv, a mutação V30M é a mais disseminada pelo mundo, sendo endêmica em Portugal, Suécia e Japão e, provavelmente, a mais comum no Brasil. Outra mutação bastante frequente é a V122I, que está presente em 3,4% dos afro-americanos e relacionada ao desenvolvimento de cardiopatia em pacientes acima dos 60 anos de idade. ^7^^,^^9^^,^^16^^,^^17^

A Tabela 1 resume as características demográficas e clínicas dos subtipos AL, ATTRv e ATTRw*t*.

**Tabela 1:** Aspectos demográficos e de apresentação clínica, comparativo entre as formas AL, ATTRv e ATTRwt

Aspectos demográficos	AL	ATTRv	ATTRwt
Idade de início (anos)	> 60 anos	Depende do genótipo	> 70 anos
Gênero	Leve predominância em homens	Sem predominância	Predominância em homens
Origem étnica	Nenhuma	Mutação mais frequente: Afrodescendentes americanos = Val122Ile Portugueses = Val30Met	Nenhuma
Prevalência/Incidência	10 casos/milhão de pessoas/ano, aumenta com a idade	Variável, conforme o genótipo	Desconhecida, aumenta com a idade
**Aspectos clínicos**			
ICFEP	√	√	√
Neuropatia periférica e/ou autonômica	√	√	-
Proteinúria	√	-	-
Púrpura periorbitária	√	-	-
Macroglossia	√	-	-
Síndrome do túnel do carpo bilateral	√	-	√
Ruptura espontânea do tendão do bíceps	-	-	√

*ICFEP: insuficiência cardíaca com fração de ejeção preservada.*

A amiloidose cardíaca por cadeias leves (AL) apresenta incidência de 6 a 10/milhão de pessoas/ano e era considerada a principal causa de AC.^18^ Com o desenvolvimento de técnicas menos invasivas de diagnóstico de AC por ATTR^19^ e a perspectiva de tratamentos efetivos, o número de casos diagnosticados, especialmente de ATTRwt, vem aumentando de forma expressiva,^20^ sendo atualmente a causa mais comum de AC. Estudos demonstram que 13%^21^ dos pacientes com ICFEP e 25% das necropsias de idosos,^22^,^23^ principalmente do sexo masculino, apresentam depósito de ATTR no coração.^24^

Muito mais que uma doença rara, na verdade, a AC é uma condição subdiagnosticada. Dados recentes nos EUA registram o progressivo aumento na prevalência, aumentando de 18 para 55,2 (100.000 pessoas-ano),^25^ e fortalecem o conceito da falta do diagnóstico que equivocadamente era considerado como inexistência da doença. A jornada do paciente até o diagnóstico é longa, e estima-se que exista um atraso de mais de 2 anos do início dos sintomas até o diagnóstico, passando, em média, por cinco diferentes profissionais.^26^ Assim, iniciativas que disseminem o conhecimento sobre AC são fundamentais, fazendo com que os clínicos e cardiologistas pensem nessa entidade, visando a um diagnóstico mais precoce e orientasção adequada da terapêutica, melhorando o prognóstico e a sobrevida dos pacientes.

Com relação ao prognóstico, a amiloidose AL causa envolvimento multiorgânico, de caráter mais agressivo em relação aos demais subtipos. O diagnóstico tardio ainda faz com que a mortalidade precoce nos primeiros 6 a 12 meses seja elevada em decorrência de complicações da cardiopatia avançada.^7^,^8^ Na ATTR, a sobrevida mediana estimada para o subtipo *wild type* é de 3,6 anos e, na ATTRv, o prognóstico depende da mutação. Nos casos de fenótipo neurológico, a progressão da neuropatia leva à incapacidade sensitivo-motora, mas a mortalidade é mais relacionada a comprometimento cardíaco.^12^,^13^

## 3. Manifestações Neurológicas

Mutações no gene TTR estão associadas a uma grande variedade de manifestações clínicas, que refletem o depósito da proteína variante (TTRv) em diferentes tipos de tecidos, sendo o envolvimento cardíaco e o do sistema nervoso periférico os mais frequentes; o primeiro particularmente causado pela mutação V122I e o segundo, pela mutação V30M.^27^

Neste capítulo, descreveremos as principais manifestações neurológicas que deveriam levantar a possibilidade de ATTRv.

As manifestações neurológicas na ATTRv podem se dividir em: neuropatia periférica, manifestações tardias de acometimento do sistema nervoso central (SNC) ligadas à angiopatia amiloide e manifestações do SNC associadas à infiltração oculomeníngea.

### 3.1. Neuropatia Periférica

A forma clássica de acometimento dos nervos periféricos na ATTRv é a polineuropatia axonal, autonômica-sensitiva-motora de evolução comprimento dependente, ou seja: afeta inicialmente segmentos mais distais dos membros, sobretudo os inferiores, e evolui para o acometimento de segmentos proximais e para os membros superiores.^28^,^29^

Na forma de início precoce (< 50 anos), em geral associada à mutação ATTRv V30M (V50M), as fibras finas, pouco ou não mielinizadas (fibras autonômicas, do calor, do frio e da dor) são as acometidas inicialmente, seguindo-se, à medida que a doença progride, comprometimento das fibras grossas, muito mielinizadas, responsáveis pelas sensibilidades vibratória, cinético-postural e pela motricidade. Assim, os primeiros sintomas são disfunção erétil, saciedade precoce, náuseas, vômitos, diarreia, constipação, alternância de diarreia com constipação, hipotensão ortostática, síncopes, arritmias, alteração da condução atrioventricular, olho seco, retenção ou incontinência urinária, dor neuropática, perda das sensibilidades ao calor e ao frio e uma importante perda ponderal. Já nessa fase inicial, podem surgir lesões indolores, mal perfurante plantar e suas repercussões, tais como infecções localizadas, celulite, osteomielite e até septicemia. Após alguns anos, surgem instabilidade à marcha e fraqueza por atrofia muscular, sempre evoluindo de distal para proximal.^28^,^29^

Já nas formas tardias, a neuropatia, desde o início, compromete todos os tipos de fibras, e a disautonomia não é tão importante, pelo menos na fase inicial da doença. Estas formas tanto podem estar associadas à mutação TTRv V30M quanto a várias outras mutações, e a evolução costuma ser mais agressiva. Em estudo brasileiro, 26 % dos pacientes com ATTRv V30M tiveram início tardio.^30^

A síndrome do túnel do carpo bilateral é uma manifestação frequente na ATTRv, podendo ser a manifestação inicial. Está associada a qualquer mutação, mas é particularmente importante em algumas delas, incluindo a TTR V122I, que aparenta ser particularmente frequente no Brasil, associada à doença cardíaca, a quem pode preceder por vários anos.^31^

### 3.2. Manifestações do Sistema Nervoso Central

O prolongamento da sobrevida, inicialmente associada ao transplante hepático e agora pelos novos medicamentos, tem possibilitado o aparecimento de manifestação que antes eram incomuns. A produção de TTR pelo plexo coroide (apenas 2% do total), a longo prazo, tanto está associada a uma angiopatia amiloide como à infiltração meníngea. A angiopatia amiloide se manifesta como episódios focais tanto do tipo deficitário *stroke-like*, *TIA-like*, ou *aura-like*, quanto do tipo irritativo epilético. Nos casos mais graves, pode se instalar isquemia ou mesmo hemorragia intracraniana. Dentre outras manifestações neurológicas, destacam-se: comprometimento auditivo; migrânia; demência; síndrome cerebelar; mielopatia e radiculopatia.

Algumas mutações raras têm predileção pelo acometimento oculocerebral e constituem quadros de amiloidose oculoleptomeníngea (ATTR Y69H: oculocerebral; Val30Gly: oculoleptomeníngea).^32^

**Estágios da neuropatia**: Os estágios de Coutinho para ATTRv são demarcados pela polineuropatia. O estágio 1 é aquele em que a polineuropatia sensitivo-motora afeta a marcha, mas o paciente não necessita de apoio para deambular. No estágio 2, um ou mais apoios são necessários para a deambulação. No estágio 3, o paciente está restrito à cadeira de rodas ou ao leito.

### 3.3. Análise Genética

A ATTRv é uma doença de herança autossômica dominante, mas que apresenta penetrância variável, que é ao mesmo tempo mutação dependente, idade dependente e também sofre influência regional.^33^ A mutação TTRv V30Met, por exemplo, tem penetrância em Portugal de 80% aos 50 anos e de 91% aos 70 anos. Na Suécia, a mesma mutação tem os valores de 11% e 36%, respectivamente.

**Figura 2:**
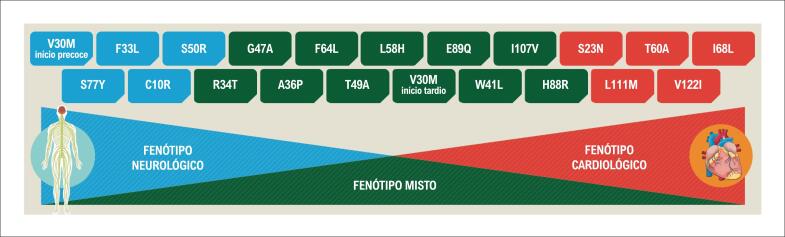
Distribuição das diferentes mutações da ATTRv associadas a fenótipos neurológicos, cardiológicos ou mistos.

Pelo menos 140 diferentes mutações foram descritas até o momento, mas nem todas são patogênicas. Algumas são polimorfismos já bem determinados, enquanto outras ainda têm significado indeterminado.^34^ Essas duas características implicam que o teste diagnóstico deve ser sempre o sequenciamento completo de gene TTR e que a interpretação das variantes raras ou ainda não descritas deve ser cuidadosa. Dentre as mutações patogênicas, existem as que causam preferentemente neuropatia (TTRv V30Met), as que causam preferentemente cardiopatia (TTRv V122I) e as que causam tanto neuropatia como cardiopatia (Leu58Hist) (Figura 2).^35^ Deve-se considerar, no entanto, que a correlação genotípica/fenotípica não é estrita.

Um capítulo especial dentre os aspectos genéticos é o teste pré-sintomático, ou seja, o teste de parentes de pessoas sabidamente afetadas. Diferentemente do teste diagnóstico, ele deve ser feito por pessoas treinadas e deve haver uma equipe de suporte, incluindo psicólogo. Deve incluir obrigatoriamente uma fase de preparação, pré-diagnóstica, o teste genético e uma fase de suporte, pós-resultado. Não deve ser realizado em crianças, e somente deve ser aplicado quando o candidato manifestar expressamente que esta é sua vontade e for considerado psicologicamente preparado.^36^

## 4. Manifestações Cardiovasculares

A AC cursa com infiltração da matriz extracelular cardíaca por fibrilas amiloides, resultando em um aumento progressivo da espessura da parede ventricular e aumento acentuado na rigidez da câmara, acarretando em comprometimento da função diastólica, levando à insuficiência cardíaca com fisiologia restritiva.^37^ A função sistólica também é comprometida, normalmente refletida por tensão longitudinal anormal, apesar de uma fração de ejeção normal, que pode se encontrar preservada até os estágios finais da doença.^38^^–^^40^ É frequente a infiltração amiloide atrial, levando à disfunção contrátil. Os depósitos também podem ocorrer nas válvulas cardíacas, geralmente sem causar disfunções importantes, e na região perivascular.^3^

Dessa forma, a manifestação clínica mais frequente é a síndrome de insuficiência cardíaca, mais comumente com fração de ejeção preservada (ICFEP), mas que pode evoluir com queda da fração de ejeção nas fases mais avançadas da doença. A síndrome clínica pode se apresentar com sintomas predominantes de IC esquerda, com congestão pulmonar (dispneia, ortopneia, dispneia paroxística noturna), ou sintomas de IC direita (edema, ascite, hematomegalia, aumento do volume abdominal, saciedade precoce, fadiga severa) ou ambos os conjuntos de sintomas. A amiloidose cardíaca deve ser considerada no diagnóstico diferencial da etiologia da ICFEP em homens idosos,^41^ particularmente quando a história pregressa de hipertensão arterial sistêmica (HAS) não é evidente ou há aumento da espessura do septo interventricular ≥ 12 mm, levantando a possibilidade de miocardiopatia infiltrativa.^21^

Síncope e hipotensão ortostática são sintomas comuns e indicam a presença de disautonomia. Um aspecto clínico característico que pode levantar a suspeita de amiloidose é a necessidade de reduzir a dose ou descontinuar medicamentos anti-hipertensivos em pacientes com diagnóstico prévio de HAS, principalmente betabloqueadores e inibidores da enzima conversora da angiotensina/bloqueadores dos receptores de angiotensina.^15^

Pode ainda ocorrer infiltração amiloide causando doença do sistema de condução cardíaco desde as fases precoces da doença, com graus variáveis de bloqueio atrioventricular, resultando, em alguns casos, em bradicardia de alto risco, com necessidade de implante de marca-passo. Outra alteração importante é decorrente do endurecimento das paredes atriais, com taxas elevadas de arritmias atriais, incluindo a fibrilação atrial, e presença de trombos atriais, sendo o acidente vascular encefálico cardioembólico uma manifestação clínica comum, mesmo em indivíduos com ritmo sinusal. Arritmias ventriculares complexas parecem ser frequentes nas fases avançadas da doença, aspecto mais bem documentado na amiloidose AL.

### 4.1. Elevação do Grau de Suspeita da Amiloidose Cardíaca

A amiloidose cardíaca, particularmente na forma ATTR, é frequentemente subdiagnosticada por razões associadas à avaliação médica e por características da própria doença, incluindo: conhecimento fragmentado entre diferentes especialidades e subespecialidades, escassez de centros e especialistas dedicados ao manejo dessa doença, conhecimento equivocado acreditando-se ser uma doença rara e incurável, heterogeneidade fenotípica e genotípica nas formas ATTR.^42^ Vale ressaltar que o diagnóstico precoce da amiloidose cardíaca é fundamental, pois o prognóstico piora rapidamente com a deposição contínua da proteína amiloide e subsequente avanço da disfunção dos órgãos.

Dessa forma, o reconhecimento de “sinais de alerta” pode auxiliar na presunção do diagnóstico da amiloidose cardíaca nos pacientes com IC,^35^,^43^ sendo os mais relevantes resumidos na Tabela 2.

**Tabela 2:** Pistas clínicas que podem levantar a suspeita de amiloidose cardíaca em pacientes com manifestações de insuficiência cardíaca

**História clínica e exame físico**
ICFEp, particularmente em homens idosos (acima de 65 anos)
Intolerância a iECA/BRA/INRA e/ou betabloqueadores
Bloqueio AV inexplicado com implante prévio de marca-passo
Síndrome do túnel do carpo bilateral
Estenose do canal vertebral
Ruptura do tendão do bíceps
Polineuropatia sensorial/motora não explicada (parestesia, dor neuropática, fraqueza)
Disfunção autonômica (hipotensão postural, diarreia pós-prandial alternando com constipação, disfunção erétil)
Púrpura periorbitária espontânea ou com traumatismo mínimo
Macroglossia
Opacidade vítrea e alterações pupilares
História familiar de miocardiopatia ou polineuropatia
**Exames de imagem**
Fenótipo infiltrativo ao ecocardiograma (SIV ≥ 12 mm), hipertrofia biventricular, hiper-refringência do miocárdio, espessamento valvar, espessamento do septo interatrial
Espessamento concêntrico das paredes do VE com amplitude do QRS reduzida ou não aumentada na proporção do aumento da espessura das paredes do VE
**Pistas combinadas**
Estenose aórtica em pacientes idosos (acima de 60 anos) com baixo fluxo/baixo gradiente
Apresentação clínica de miocardiopatia hipertrófica iniciada tardiamente (acima de 60 anos)

*ICFEp: insuficiência cardíaca de fração de ejeção preservada; VE: ventrículo esquerdo; SIV: septo interventricular; iECA: inibidor da enzima conversora da angiotensina; BRA: bloqueador do receptor da angiotensina II; INRA: inibidor da neprilisina e do receptor da angiotensina.*

Síndrome do túnel do carpo bilateral é muitas vezes um dos primeiros indicadores de ATTR, é a manifestação não cardíaca mais comum e pode preceder os sintomas de insuficiência cardíaca em vários anos. Um estudo recente observou que aproximadamente 50% dos indivíduos com ATTRwt apresentava síndrome do túnel do carpo 5 a 7 anos antes do diagnóstico.^44^ Estenose lombar e ruptura atraumática do tendão do bíceps também foram identificados como manifestações clínicas de deposição extracardíaca na ATTRwt. A ruptura do tendão do bíceps pode estar presente em até 33% dos casos de ATTRwt.^45^ Por outro lado, na forma AL, a presença de macroglossia e a púrpura periorbitária são altamente específicas, porém ocorrem em apenas 15% dos casos.^46^ A presença de polineuropatia sentitivo/motora ou disautonomia em pacientes com IC deve levantar a suspeita de AC.^47^,^48^

Outros sinais de alerta para a doença podem emergir de alterações típicas nos exames complementares cardiológicos de rotina, e são abordadas em tópicos específicos deste documento.

Vale salientar, ainda, que a amiloidose cardíaca pode muitas vezes apresentar-se simulando outras cardiopatias. A amiloidose deve ser considerada como uma das possíveis etiologias de pacientes que apresentam fenótipo de cardiomiopatia hipertrófica, particularmente se iniciada em fases tardias da vida (> 60 anos). O padrão assimétrico de hipertrofia miocárdica (HVE) nos pacientes com amiloidose ATTR difere dos pacientes com forma AL, geralmente simétrico. Em estudo de 263 pacientes com AC por ATTR confirmada e comparados com 50 pacientes com a forma AL, observou-se na forma ATTR presença de hipertrofia assimétrica em 79% dos casos, simétrica em 18% e 3% sem HVE.^49^

Pacientes idosos portadores de estenose aórtica grave de baixo fluxo e baixo gradiente podem exibir AC em até 10% a 15% dos casos, com prognóstico desfavorável.^50^

## 5. Exames Complementares Diagnósticos

### 5.1. Eletrocardiograma

**Figura 3:**
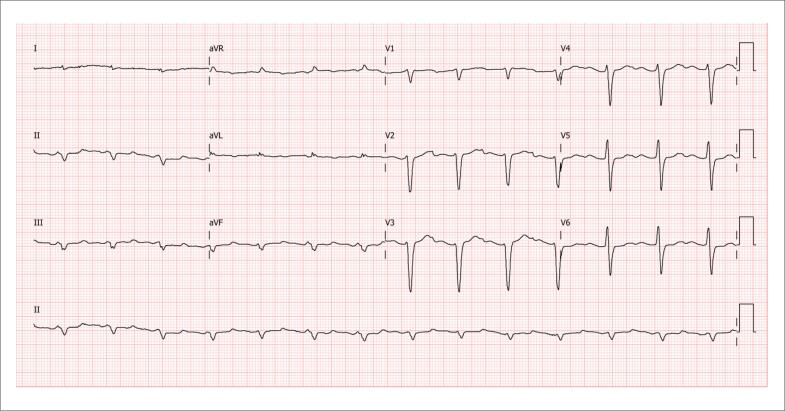
Imagem ilustrativa de eletrocardiograma (ECG) de paciente com amiloidose cardíaca (AC) forma ATTRwt, mostrando baixa voltagem em derivações periféricas, ausência de progressão de R em derivações precordiais V1 a V3 (padrão de pseudoinfarto) e BAV de I grau. (Imagem de arquivo pessoal dos autores)

O eletrocardiograma (ECG) é um exame essencial na avaliação diagnóstica e no planejamento terapêutico dos pacientes, sendo importante sua interpretação em conjunto com as informações clínicas e ecocardiográficas. Embora o achado de sinais de baixa voltagem ao ECG tenha grande especificidade no diagnóstico de infiltração miocárdica secundária à AC, este não é o achado mais prevalente na doença. A ausência de progressão de ondas R em derivações precordiais, simulando uma zona elétrica inativa anterosseptal (padrão de pseudoinfarto) é achado muito mais frequente, chegando à prevalência de 60% a 70% dos casos com diagnóstico confirmado, independentemente do tipo de amiloidose (Figura 3).

Nos casos de AC por ATTR, menos de 40% dos pacientes com diagnóstico confirmado por biópsia apresentam sinais de baixa voltagem ao ECG.^51^

Assim, a ausência de critérios de baixa voltagem ao ECG, ou mesmo a presença de sinais de sobrecarga de VE, não deve afastar a suspeita diagnóstica de AC, especialmente a ATTR. A desproporção da voltagem em relação à espessura miocárdica também é sinal de alerta importante e alcança prevalência de 73% a 80% em pacientes com AC, independentemente de seu tipo.^52^,^53^

Dentre as alterações do ritmo cardíaco, a fibrilação atrial é mais prevalente em pacientes com ATTR, assim como os bloqueios atrioventriculares.

### 5.2. Ecocardiograma

**Figura 4:**
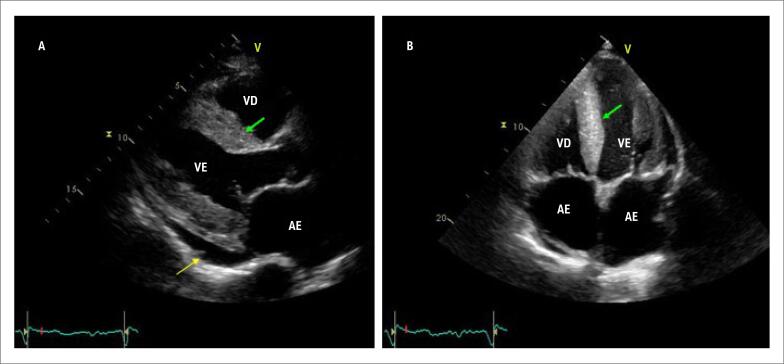
Apresentação ecocardiográfica clássica de amiloidose cardíaca (AC). Em A, a projeção paraesternal longitudinal apresenta o ventrículo esquerdo (VE) de tamanho normal, mas com espessura de paredes aumentada e aspecto granulado em septo interventricular (seta verde). Há, ainda, sinais de aumento do átrio esquerdo (AE) e leve derrame pericárdico (seta amarela). Em B, a projeção apical 4c evidencia os átrios grandes, ventrículos de dimensões normais e aumento de espessura de paredes, também com aspecto granulado do septo interventricular (seta verde). As valvas mitral e tricúspide são levemente espessadas. VE: ventrículo esquerdo; VD: ventrículo direito; AD: átrio direito. (Imagens de arquivo pessoal dos autores).

A ecocardiografia deve ser realizada em todos os pacientes com suspeita clínica da doença. Os achados clássicos de AC geralmente estão presentes em uma fase avançada da doença e englobam as características de uma cardiomiopatia restritiva, do tipo infiltrativa. As dimensões do ventrículo esquerdo (VE) não são aumentadas, os volumes são normais ou reduzidos e há aumento da espessura de paredes ventriculares. O aumento de dimensões atriais é comum, refletindo uma disfunção diastólica precoce e progressiva, com aumento das pressões de enchimento. As valvas atrioventriculares podem estar espessadas e as regurgitações valvares (mitral e tricúspide) são funcionais. Pode, ainda, haver sinais sugestivos de infiltração do septo interatrial e também elevação da pressão sistólica da artéria pulmonar. O ventrículo direito também pode estar acometido. É muito comum a presença de derrames pleurais e pericárdicos e, em casos em que há intensa infiltração tecidual, é possível observar o clássico, embora subjetivo, aspecto granular da imagem de paredes miocárdicas (*granular sparkling*) (Figura 4)^35^,^54^

Na AC, a fração de ejeção do VE (FEVE) permanece normalmente preservada até os estágios mais avançados da doença, mas a função contrátil longitudinal é reduzida precocemente.^55^ Quantitativamente, a função sistólica pode ser avaliada pelo modo 2D (bidimensional), utilizando cálculo de volumes e FEVE e por técnicas derivadas do Doppler como a estimativa do dP/dT do ventrículo esquerdo.^56^

**Figura 5:**
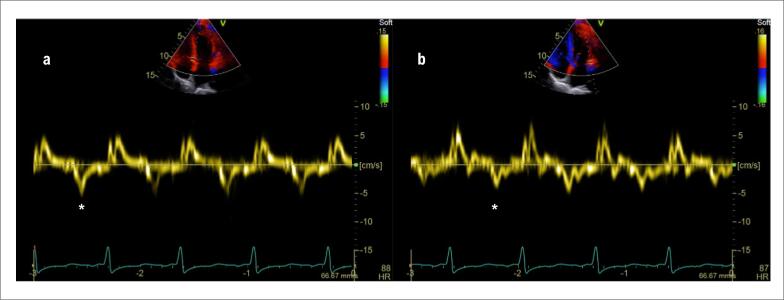
Imagens de Doppler tecidual em anel mitral. Há redução da velocidade da onda e’(*) tanto no Doppler tecidual do anel mitral medial (a) quanto no do lateral (b). Ambas as velocidades se encontram abaixo de 4 cm/s (VR > 8 cm/s). (Imagens de arquivo pessoal dos autores).

A avaliação do padrão de enchimento diastólico dos ventrículos é essencial e frequentemente demonstra algum grau de disfunção diastólica. Nas fases iniciais, é possível observar alterações compatíveis com disfunção diastólica tipo I (inversão da relação das ondas E/A do influxo mitral, prolongamento do tempo de relaxamento isovolumétrico e diminuição da rampa de desaceleração diastólica inicial. No registro das velocidades miocárdicas pelo Doppler tissular, observa-se redução da velocidade da onda e’) (Figura 5).

Com a progressão da doença, pode se instalar padrão pseudonormal de disfunção diastólica (relação E/A preservada e pelo tempo de desaceleração normal) por consequência da elevação da pressão no átrio esquerdo.

Na fase mais adiantada da doença, observa-se o padrão restritivo do enchimento ventricular (aumento da relação E/A > 2, diminuição do tempo de relaxamento e acentuação da inclinação da rampa de desaceleração da onda E).

**Figura 6:**
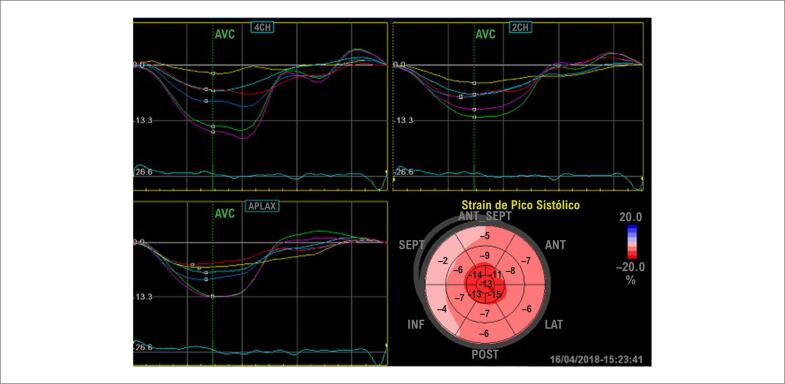
Análises da deformação miocárdica global e regional de VE em paciente com amiloidose cardíaca, mostrando a representação gráfica paramétrica, conhecida como bulls eye, demonstram os segmentos apicais em vermelho-escuro (valores preservados), e os segmentos basais em vermelho-claro (valores reduzidos) com o padrão de “cereja no bolo”. (Imagem de arquivo pessoal dos autores).

O estudo da deformação miocárdica permite identificar, precocemente em relação à FEVE, sinais de disfunção miocárdica.^57^^–^^59^ A deformação sistólica global longitudinal (GLS) – função predominantemente exercida pelo endocárdio – encontra-se precocemente reduzida.^60^ O padrão regional da deformação miocárdica também está frequentemente alterado apresentando o padrão de *apical sparing*,^61^,^62^ traduzido como o padrão de “cereja do bolo”. A melhor visualização desse aspecto é possível na imagem paramétrica do VE, conhecida como *bulls eye* (Figura 6).

Outras análises da deformação miocárdica – tais quais a capacidade em estudar a função sistólica de VD,^63^ a função atrial^65^ e, ainda, estimar o trabalho sistólico miocárdico^59^ – têm sido aplicadas a pacientes com AC e vêm demonstrando boa acurácia diagnóstica.

### 5.3. Ressonância Magnética Cardíaca

A ressonância magnética cardiovascular (RMC) dispõe de técnicas que permitem avaliar de forma precisa as alterações teciduais miocárdicas que ocorrem na AC.^65^ Classicamente, o depósito de miofibrilas leva ao aumento de espessura da parede miocárdica do VE e do septo interatrial, que podem ser visualizadas pelas técnicas morfológicas de RMC.^66^,^67^ Outra alteração tecidual é o aumento do conteúdo total de água no miocárdio, que pode ser derivado do aumento do volume extracelular (VEC) causado diretamente pelo depósito proteico e sua atração osmótica de água, assim como o aumento de água intracelular em miócitos sofrendo a agressão citotóxica do depósito ou mesmo diminuição da perfusão miocárdica (distância aumentada dos capilares e/ou obstrução destes pelo depósito).

O aumento global de água tecidual miocárdica leva a aumento dos tempos médios de relaxamento do hidrogênio, sejam eles T1 (longitudinal) ou T2 (transversal).^68^ No entanto, a alteração tecidual miocárdica mais dramática e relevante na AC é o aumento extremo do VEC miocárdico, causado nas fases clínicas da doença, não só pelo depósito de fibrilas amiloides, mas também pela fibrose miocárdica de reparação.

Assim, o conjunto do depósito de miofibrilas e fibrose intersticial pode ser facilmente detectado de forma muito precisa, e até mesmo quantificado pelas técnicas de realce tardio (RT)^67^,^69^ e cálculo do volume extracelular do miocárdio.^70^^–^^72^ Apenas como exemplo inicial, valores de espaço extracelular do miocárdio normal estão em torno de 25%, enquanto, na AC, pode atingir valores tão altos quanto 60% (principalmente na ATTR).^72^,^73^

O contraste utilizado na RMC baseia-se em gadolínio que, por sua vez, é ligado a um quelante macromolecular que não permite sua passagem pela membrana celular íntegra, distribuindo-se assim exclusivamente no VEC do miocárdio. A imagem de RT permite levantar a suspeita de AC pelo seu padrão de distribuição (global subendocárdico, não envolvimento apical do VE e de distribuição que não respeita território vascular coronariano).^67^

#### 5.3.1. Avaliação da Morfologia e da Função Cardíaca

A AC pode modificar a aparência de todas as câmaras cardíacas.^74^,^75^ Desde as etapas iniciais, podem ser observadas alterações no plano atrial, com dilatação daquelas cavidades e aparente espessamento do septo interatrial que, na maior parte das vezes, é composta por gordura.^74^ Em etapas mais adiantadas de comprometimento cardíaco, quando há diminuição da função atrial, observam-se sinais de fluxo lento e de trombos no apêndice atrial esquerdo, que podem não ser observados à ressonância no caso de haver artefatos de ritmo e de não se utilizarem séries específicas para este fim.

A AC também é habitualmente associada à presença de aumento da espessura miocárdica, que é, na maior parte das vezes, mais expressiva do que nos casos secundários à hipertensão, e a espessura do músculo cardíaco geralmente é maior nos casos de ATTR do que nos casos AL.^52^,^76^ Este aumento de espessura pode ser concêntrico ou excêntrico,^74^,^75^ e pode envolver o ventrículo direito.^74^,^75^ Durante muito tempo, a fração de ejeção pode estar preservada, e as modificações mais precoces da função ventricular incluem restrição diastólica e alterações da deformidade ventricular (*strain*).^75^,^76^

**Figura 7:**
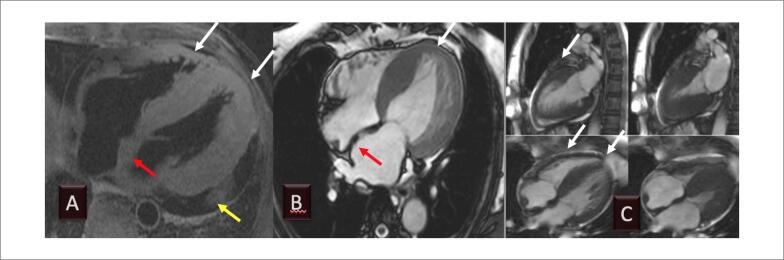
Exemplo de RMC de pacientes com amiloidose por TTR, mostrando derrame pericárdico (A, seta amarela e C), aumento das dimensões dos átrios (A e B), aumento da espessura do septo interatrial (A e B, setas vermelhas) e das paredes ventriculares (A a C, setas brancas). A função diastólica é diminuída, mas a contratilidade pode estar preservada até as fases mais avançadas da doença. (Imagem de arquivo pessoal dos autores).

Em consequência da disfunção diastólica, não é raro observar derrame pericárdico ou pleural.^74^ As características morfológicas avaliadas pela ressonância (Figura 7) são úteis e podem sugerir o diagnóstico; contudo, na maior parte das vezes, há a necessidade de se realizar a caracterização tecidual, pelas técnicas de realce tardio e de mapa T1, como discutiremos em outras seções deste documento.

#### 5.3.2. Avaliação pelo Realce Tardio

**Figura 8:**
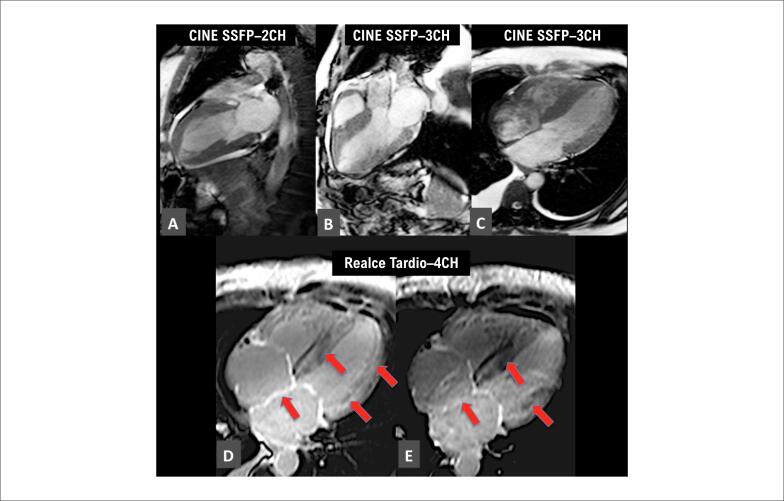
Exemplo de paciente com amiloidose tipo TTR com hipertrofia concêntrica de ventrículo esquerdo (VE) e aumento de ambos os átrios nas imagens em cine (A a C). Realce tardio apresenta padrão predominantemente transmural difuso (setas vermelhas, D e E). (Imagem de arquivo pessoal dos autores).

A técnica de RT após injeção do contraste gadolínio tem sido amplamente reconhecida como um dos pilares no diagnóstico por imagem da AC.^77^,^78^ O contraste de gadolínio ao atravessar o espaço intersticial no tecido normal não sofre atrasos e rapidamente o deixa, ficando o tecido normal escuro. Em casos em que há presença de deposição amiloide no espaço intersticial, este passa a reter o trânsito do contraste à base de gadolínio, e o miocárdio brilha (realce) nas sequências dedicadas, e foi demonstrada correlação anatomopatológica.^65^ Foram descritos padrões de infiltração amiloide: subendocárdico, transmural e focal, sendo este último menos frequente (Figura 8).^67^ Metanálise recente estabeleceu em 85% a sensibilidade e em 92% a especificidade do realce tardio na amiloidose.^79^

Em série com 250 pacientes com amiloidose de distintas formas, a presença de RT com padrão transmural esteve associada a um risco de morte 5,4 vezes maior (IC:2,1-13,7; p < 0,0001).^80^ Além disso, o RT apresenta efeito prognóstico incremental aos marcadores cardíacos na AC forma AL^81^ ou de forma isolada.^82^

#### 5.3.3. Mapas de T1

Diferentes grupos têm investigado a utilidade de mapas de T1 derivados da RMC para melhorar o desempenho diagnóstico e prognóstico na AC.^70^^,^^71^,^73^^,^^83^^,^^84^ Tanto os dados de T1 nativo, que não necessitam de administração de contraste, assim como os dados de VEC, têm identificado com eficácia pacientes com AC, mostrando valores marcadamente elevados para o T1 nativo e VEC comparado a pacientes-controles saudáveis^70^^,^^73^^,^^83^ – alterações que podem ser detectadas antes da presença de RT.^70^^,^^72^^,^^73^ Portanto, combinar mapeamento T1 nativo e medições de VEC pode ajudar a delinear a carga amiloide e confirmar o diagnóstico de AC .^85^,^86^

### 5.4. Cintilografia Cardíaca com Radiotraçadores Ósseos

Os radiotraçadores marcados com tecnécio-99m derivados do bifosfonato, originalmente desenvolvidos para imagens ósseas, encontraram um novo papel como ferramenta diagnóstica não invasiva da AC por ATTR.^1^^–^^7^ As imagens com radiotraçadores ósseos permitem, de modo seguro, o diagnóstico não invasivo de AC por ATTR, uma vez excluída a presença de gamopatia monoclonal.^91^

Os principais radiotraçadores ósseos marcados com ^99m^Tc utilizados para o diagnóstico da AC por ATTR são o ^99m^Tc-pirofosfato, o ^99m^Tc-DPD (ácido 3,3-difosfono-1,2-propanodicarboxílico) e o ^99m^Tc-HMDP (hidroximetilenodifosfonato marcado com ^99m^Tc).^1^^–^^7^ O ^99m^Tc-pirofosfato é o único disponível no Brasil. É importante ressaltar que o ^99m^Tc-MDP (metilenodifosfonato marcado com ^99m^Tc), apesar de comprovadamente eficiente para a realização da cintilografia óssea, apresenta baixa sensibilidade para o diagnóstico da AC por ATTR e não deve ser usado para esta finalidade.^89^

Embora o componente estrutural do depósito amiloide ao qual o ^99m^Tc-pirofosfato se liga no coração não seja conhecido, um mecanismo de captação cálcio-dependente é amplamente aceito.^93^ Em animais, verificou-se a existência de vários sítios de ligação: microcalcificações, depósitos de cálcio, pirofosfato intracelular e macromoléculas intracelulares. Provavelmente, o mecanismo de captação de ^99m^Tc-pirofosfato no miocárdio esteja relacionado à presença de microcalcificações.^88^ A AC por ATTR apresenta mais microcalcificações que a AL e apresenta maior captação de ^99m^Tc-pirofosfato, enquanto a amiloidose cardíaca AL tem mínima ou nenhuma avidez pelos radiotraçadores ósseos; além disso, a AC por ATTR apresenta evolução mais indolente, o que propicia mais microcalcificações e, consequentemente, maior acúmulo do radiotraçador.

Embora os achados do ecocardiograma e da RMC possam ser indicativos de AC, eles não são capazes de diferenciar a forma ATTR da forma AL. Esta é a principal vantagem da cintilografia cardíaca com ^99m^Tc-pirofosfato: um método simples, de fácil execução, amplamente disponível, com baixa dosimetria, capaz de diferenciar de forma não invasiva e com alta especificidade a AC por ATTR da AL, orientando assim a conduta. Esta diferenciação é útil, pois as formas AL e ATTR têm implicações prognósticas e terapêuticas completamente diversas.

O papel dos radiotraçadores ósseos na ATTR foi recentemente reavaliado por um grupo internacional de vários centros com *expertise* em AC: em análise cumulativa de 1.217 pacientes, sendo 867 com amiloidose confirmada por biópsia e 360 portadores de cardiomiopatia não amiloide, a cintilografia foi altamente sensível (99%) e específica (86%) para ATTR.^19^ Neste estudo, demonstrou-se ainda que o achado combinado de uma cintilografia com radiotraçador ósseo positiva em pacientes sem evidência de proteína monoclonal detectável na urina ou soro (utilizando medida sérica de cadeias leves livres e eletroforese com imunofixação) foi 100% específica para AC por ATTR, levando os autores a concluir que a cintilografia permite a detecção de modo acurado sem a necessidade de biópsia cardíaca.^19^ Outro estudo recente com casos agrupados de três centros dos EUA mostrou que, entre um total de 171 pacientes (121 ATTR, 34 AL e 16 ICFEP não amiloide), ^99m^Tc-pirofosfato apresentou sensibilidade de 88% e especificidade de 88% para ATTR quando se utilizou apenas a avaliação visual (escore ≥ 2) ^94^ Quando se utilizou a análise semiquantitativa (relação de contagens coração/contralateral > 1,6), a sensibilidade foi de 91% e a especificidade, 92%, para a detecção de ATTR. Além disso, ao levar em conta todas as variáveis, uma relação de contagens coração/contralateral ≥ 1,6 foi preditiva de pior prognóstico, ou seja, associada com pior sobrevida em pacientes com AC por ATTR.^94^ Vraniam et al.^95^ também demonstraram que, em pacientes com suspeita de AC, a intensidade de captação cardíaca de ^99m^Tc-pirofosfato foi preditiva de mortalidade geral e hospitalização por IC. Nesses estudos, a combinação da intensidade de captação do radiotraçador no miocárdio (relação coração/contralateral) com variáveis anatômicas, funcionais e biomarcadores melhorou a estratificação de risco.

#### 5.4.1. Aspectos Técnicos Recomendados para Aquisição das Imagens

Não é necessário preparo. As imagens são obtidas após a administração venosa de 10 a 25 mCi (370 a 925 Mbq) de ^99m^Tc-pirofosfato (dosimetria: 3,2 mSv para corpo inteiro para 15 mCi). Imagens planas e tomográficas do tipo SPECT (*single photon emission computed tomography*) do tórax são realizadas 1 e 3h após a administração do radiofármaco.

- *Imagens planas do tórax*: podem ser obtidas nas projeções anterior, oblíqua anterior esquerda e lateral esquerda, utilizando o fotopico do ^99m^Tc (140 keV, janela de 15%), colimador de baixa energia/alta resolução e matriz de 256×256, 500–750 mil contagens.
- *Imagens SPECT do tórax:* obtidas com matriz de 128×128 (64×64 é aceitável), rotação de 180 graus de oblíqua anterior direita a oblíqua posterior esquerda (360 graus é aceitável), 1 imagem a cada 3 a 6 graus. Se disponíveis, imagens SPECT/CT (SPECT associado à tomografia computadorizada) proporcionam maior segurança na interpretação das imagens.
- *Imagens mínimas recomendadas:* SPECT de 1h e imagens planas de 1 e 3h na projeção anterior. Recomenda-se a utilização de imagens SPECT, para diferenciar captação difusa no miocárdio de persistência do radiofármaco no *pool* sanguíneo, captação focal por infarto do miocárdio e sobreposição óssea. Além disso, imagens planas de 1 e 3h são úteis para a quantificação e para acompanhar o *whashout* do *pool* sanguíneo, que é variável, sendo, por exemplo, bem mais lento em pacientes com insuficiência renal.

#### 5.4.2. Análise das imagens

**Figura 9:**
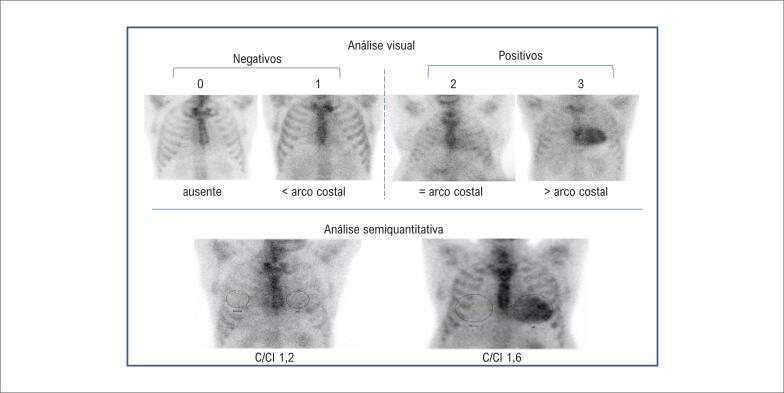
Cintilografias cardíacas com ^99^mTc-pirofosfato em pacientes com suspeita de amiloidose cardíaca (imagens planas na incidência anterior do tórax obtidas 3h após a administração do radiofármaco) mostrando exemplos de casos negativos e positivos para amiloidose cardíaca por ATTR. Análise visual: imagens à esquerda: casos negativos para ATTR (análise visual graus 0 e 1); imagens à direita: casos positivos para ATTR (análise visual graus 2 e 3). Análise semiquantitativa: à esquerda, caso negativo para AC por ATTR (relação coração/contralateral = 1,2 – nesse caso, as imagens de SPECT mostraram atividade no pool sanguíneo e não nas paredes do coração); à direita, caso positivo para ATTR (relação coração/contralateral = 1,6 – com imagens de SPECT que confirmaram captação nas paredes do VE).

- *Análise semiquantitativa (imagem plana de 1h):* a análise semiquantitativa foi definida para o radiofármaco ^99m^Tc-pirofosfato como sendo a razão entre a captação na projeção cardíaca e a captação no hemitórax contralateral, medidas na imagem plana de 1h na incidência anterior. Para isso, é desenhada uma área de interesse (AI) circular (ou elíptica) sobre a projeção cardíaca, sem incluir o esterno, evitando a inclusão tanto do pulmão adjacente como de áreas de hipercaptação focal em arcos costais. Uma AI idêntica (“em espelho”) é colocada no hemitórax contralateral, com os mesmos cuidados. As contagens no interior da AI cardíaca dividem-se pelas contagens da AI contralateral, obtendo-se, assim, a relação C/CL (coração/contralateral). A C/CL ≥ 1,5 à 1h identifica ATTR com elevada acurácia, caso a amiloidose AL sistêmica tenha sido excluída^90^,^91^ (Figura 9).
- *Graduação visual (imagem de 3h):* a graduação visual é feita comparando-se a captação cardíaca com a captação fisiológica nos arcos costais adjacentes, e pode ser realizada tanto nas imagens planas na projeção anterior como nas imagens SPECT ou mesmo nas imagens de corpo inteiro, obtidas 3h após a injeção do radiofármaco. A intensidade da captação é numericamente definida como grau 0 (sem captação miocárdica), 1 (captação miocárdica inferior à dos arcos costais adjacentes), 2 (captação semelhante à dos arcos costais) e 3 (superior à dos arcos costais). Intensidades de captação graus 2 ou 3 são fortemente sugestivas de ATTR, se gamopatia monoclonal tiver sido excluída (Figura 9).

Na interpretação visual, valoriza-se para diagnóstico da ATTR a intensidade de captação cardíaca igual (grau 2) ou maior (grau 3) que intensidade de captação nos arcos costais. Na análise semiquantitativa, a relação de contagens coração/contralateral ≥1,5 nas imagens de 1h e ≥ 1,3 nas imagens de 3h são consideradas altamente sugestivas de ATTR, quando confirmadas pelas imagens de SPECT. (Imagens de arquivos pessoais dos autores)

#### 5.4.3. Falso-positivos para ATTR

**Figura 10:**
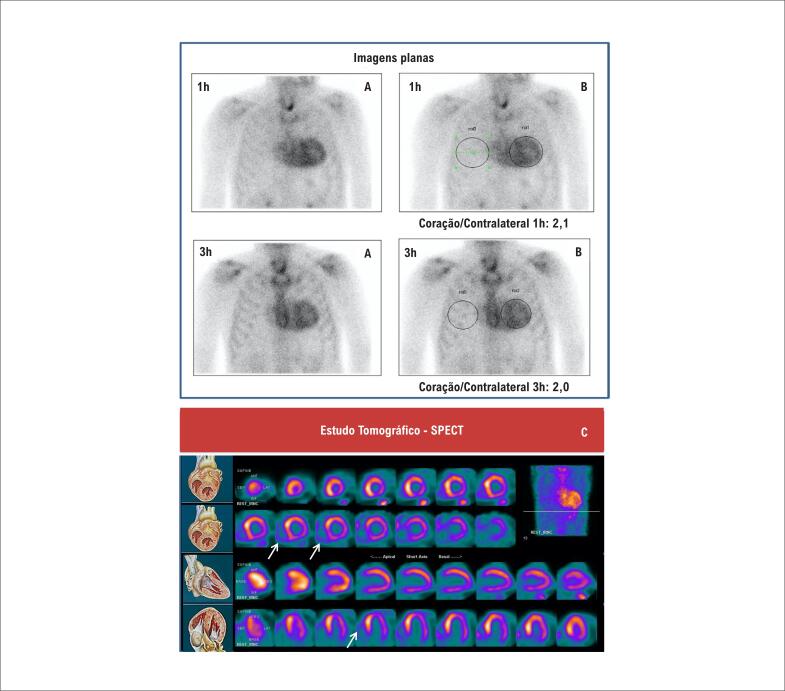
Imagens ilustrativas de paciente masculino de 76 anos com amiloidose cardíaca por ATTRv (VAL30MET). A cintilografia cardíaca com ^99m^Tc-pirofosfato (imagens planas A e B) mostrou intensa captação de radiotraçador nas imagens de 1h e de 3h. A análise visual (A) mostrou captação grau 3 tanto nas imagens de 1h quanto nas imagens de 3h (intensidade de captação miocárdica do radiofármaco maior que a dos arcos costais). A análise semiquantitativa (B) mostrou relação coração/contralateral em 1h = 2,1 e em 3h = 2,0. O estudo tomográfico (SPECT) mostrou que a captação do radiofármaco se distribui em todas as paredes do VE e confirmou acometimento do VD, já evidenciado nas imagens planas (setas). Os achados da cintilografia cardíaca com ^99m^Tc-pirofosfato indicam estudo altamente sugestivo de AC por ATTR. (Imagens de arquivos pessoais dos autores).

As características operacionais diagnósticas da cintilografia com traçadores ósseos são muito favoráveis para o seu uso clínico, com uma especificidade de 100% para o diagnóstico de ATTR quando a captação é de intensidade 2 ou 3, na ausência de gamopatia monoclonal. Entretanto, é sempre necessário enfatizar que falhas em excluir gamopatia monoclonal (quer por uso inadequado ou interpretação falha dos exames laboratoriais) implicam risco de diagnósticos imprecisos. A causa mais comum de diagnóstico errôneo de ATTR é a amiloidose AL. Estudos recentes apontam que até 22% dos pacientes com AC da forma AL podem apresentar captação grau 2 ou 3 na cintilografia com ^99m^Tc-pirofosfato.^6^ Também é importante lembrar que a utilização de imagens cintilográficas tomográficas (SPECT) é crucial na diferenciação de captação miocárdica anormal de captação residual no *pool* sanguíneo (Figura 10). A Tabela 3 lista as principais causas de falso-positivo com uso da cintilografia com ^99m^Tc-pirofosfato

**Tabela 3:** Causas de cintilografia com ^99m^Tc-pirofosfato falso-positiva (captação miocárdica de ^99m^Tc-pirofosfato que não é associada à ATTR)

1. Amiloidose cardíaca por cadeias leves (AL)
2. Captação em *pool* sanguíneo (imagens planares)
3. Fraturas de costelas (imagens planares)
4. Infarto do miocárdio (agudo ou subagudo)
5. Cardiotoxicidade por hidroxicloroquina
6. Formas raras de amiloidose cardíaca

*Fonte: adaptada de Hanna et al.* ^91^

Finalmente, é importante considerar que o estudo cintilográfico não é recomendado para o seguimento dos pacientes, visto que, até o momento, não se evidenciou correlação entre a alteração no padrão evolutivo das imagens e progressão da doença ou a resposta ao tratamento.^87^,^90^

A Tabela 4 resume os principais achados e orientações práticas para aquisição e análise das imagens para o diagnóstico de AC.

**Tabela 4:** Resumo dos principais achados sugestivos de amiloidose cardíaca nos exames complementares

Exames – achados sugestivos de amiloidose cardíaca (AC)	Dicas práticas – detalhes diagnósticos
**Eletrocardiograma (ECG)**	
Padrão de pseudoinfarto	Padrão clássico de baixa voltagem é mais comum na forma AL (até 70% dos casos), mas pouco comum na ATTR (apenas 30% dos casos)
Voltagem do QRS desproporcional ao grau de espessamento das paredes	
Baixa voltagem dos complexos QRS	É muito importante interpretar o ECG juntamente com os dados clínicos e do ecocardiograma
Arritmias atriais – fibrilação atrial	
Distúrbios de condução	BAV ou FA podem ser manifestação inicial de AC
**Ecocardiograma**	
Aumento da espessura ventricular (SIV > 12 mm)	Além das informações estruturais e funcionais do ecocardiograma convencional, a análise de deformação miocárdica é importante para identificar disfunção miocárdica mesmo com FE normal e o padrão de *apical sparing*
Aspecto granuloso/hiper-refringente do miocárdio espessado	
Espessamento das paredes do VD, além do VE	
Dilatação biatrial	
Espessamentos valvulares e de septo interatrial	
Disfunção diastólica (padrão restritivo)	
Deformação miocárdica longitudinal alterada de padrão regional poupando o ápice (*apical sparing*)	
**Ressonância magnética cardíaca (RMC)**	
Valores elevados de mapa T1 nativo	Além as informações estruturais e funcionais cardíacas que podem ser muito sugestivas, informações de realce tardio, mapa T1 e volume extracelular são importantes para sugerir presença de AC
Aumento acentuado do volume extracelular	
Realce tardio difuso transmural ou subendocárdico	
Realce tardio em paredes atriais	A RMC não permite diferenciar as formas de amiloidose AL e ATTR
Aumento da espessura das paredes de VD/VE e septo interatrial	A RMC pode ser útil para identificar outras formas de cardiomiopatia infiltrativa
**Cintilografia cardíaca com radiotraçadores ósseos**	
Captação miocárdica igual ou superior à óssea (grau 2 ou 3)	No Brasil, apenas o ^99m^Tc-pirofosfato é disponível para esta finalidade
Relação de captação coração/contralateral ≥ 1,5	Na vigência de cadeias leves negativas, cintilografia com ^99m^Tc-pirofosfato positiva faz diagnóstico de ATTR, sem necessidade de BEM
Captação de ^99m^Tc-pirofosfato nas paredes do coração confirmada por imagens de SPECT	Até 30% das AC forma AL podem exibir cintilografia positiva Cintilografia cardíaca com ^99m^Tc-pirofosfato negativa não afasta o diagnóstico de amiloidose cardíaca

*BEM: biópsia endomiocárdica; SIV: septo interventricular; GLS: strain global longitudinal; SPECT: cintilografia pelo método tomográfico de emissão; VD: ventrículo direito; VE: ventrículo esquerdo*.

### 5.5. Biomarcadores

Não existe um marcador laboratorial específico para o diagnóstico de AC.

A troponina e os peptídios natriuréticos têm se mostrado úteis para avaliar acometimento cardíaco pela amiloidose, permitindo auxílio diagnóstico não invasivo, acessível e relativamente de baixo custo.^96^ A detecção de alterações persistentes desses biomarcadores é sinal de alerta da presença de acometimento cardíaco pela amiloidose.

#### 5.5.1. Peptídios Natriuréticos

Análise de dados do registro THAOS (Transthyretin Amyloidosis Outcomes Survey), com pacientes acometidos por ATTR, demonstrou que a dosagem dos peptídios natriuréticos pode ser utilizada para auxílio diagnóstico, e foram observados valores mais elevados nos pacientes com mutações de acometimento preferencialmente cardíaco, como a V122I e V30M-tardia, do que nas formas de predomínio neurológico, como a V30M-precoce. Também foram observados maiores níveis dos biomarcadores na ATTRwt quando comparada com as formas hereditárias de predomínio neurológico. Porém, observou-se que, mesmo nos pacientes com fenótipo predominantemente neurológico, 45% a 90% apresentavam alterações desses biomarcadores, indicando algum grau de acometimento miocárdico subclínico.^97^

Observam-se maiores valores de NT-proBNP na forma AL, em comparação à ATTR. Isso se deve ao fato de as cadeias leves amiloidogênicas modularem a proteína cinase ativada por mitógeno p38 (MAPK), que diretamente promove a expressão do NT-proBNP, de forma que, para um mesmo grau de alterações hemodinâmicas nas duas formas de amiloidose, os níveis séricos da NT-proBNP podem ser maiores na forma AL.^98^

Vale ressaltar que os pacientes com ICFEP causada por ATTR apresentam valores de NT-ProBNP desproporcionalmente elevados em relação à gravidade da síndrome de IC, quando comparados aos pacientes com ICFEP não amiloide.^99^

#### 5.5.2. Troponinas

Elevação discreta e persistente dos níveis de troponina é frequentemente observada e sugere dano miocárdico subclínico em várias cardiomiopatias não isquêmicas.^100^ Contudo, tem sido relatado que os níveis são mais elevados em pacientes com AC ao se comparar a outras formas de cardiomiopatia.^21^ Estudo em pacientes com cardiomiopatia hipertrófica submetidos à biópsia endomiocárdica identificou níveis marcadamente mais elevados de troponina nos pacientes que tinham amiloidose em comparação com aqueles com cardiopatia livre de depósito amiloide, com alta sensibilidade diagnóstica.^101^

Vários mecanismos para justificar a elevação das troponinas nesses pacientes têm sido postulados: isquemia miocárdica, estresse de parede aumentado, lesão direta do miócito por citocinas inflamatórias e/ou estresse oxidativo, ativação neuro-hormonal e disfunção coronariana microvascular na insuficiência cardíaca. A disfunção microvascular na amiloidose é causada, presumidamente, por depósito intersticial e na região perivascular, pressão de enchimento ventricular aumentada e disfunção endotelial por toxicidade induzida pelas imunoglobulinas, na forma AL. Além disso, também é descrito efeito cardiotóxico direto das cadeias leves, independentemente do depósito extracelular de fibrilas, podendo justificar os níveis mais elevados de troponina em pacientes com forma AL do que em pacientes com ATTR, nos quais geralmente se observa elevação discreta e persistente.^101^

Vale ressaltar que, entre os pacientes com ATTR, muitos apresentam comorbidades, como miocardiopatia isquêmica, o que pode causar valores alterados de troponina. Sendo assim, a dosagem de troponina não deve ser utilizada para afastar ou confirmar acometimento cardíaco, mas sim como um potencial sinal de alerta para a doença, que será mais bem avaliada com exames mais específicos.

#### 5.5.3. Novos Biomarcadores

Uma série de outros biomarcadores tem sido estudada, alguns com alta especificidade para subtipos de amiloidose, como a dosagem de proteína carreadora de retinol 4 para a amiloidose hereditária pela mutação Val142Ile. No entanto, ainda mais estudos são necessários, assim como a disponibilização comercial dos testes para análises de rotina.^102^

## 6. Abordagem Diagnóstica Racional da Amiloidose Cardíaca

**Figura 11:**
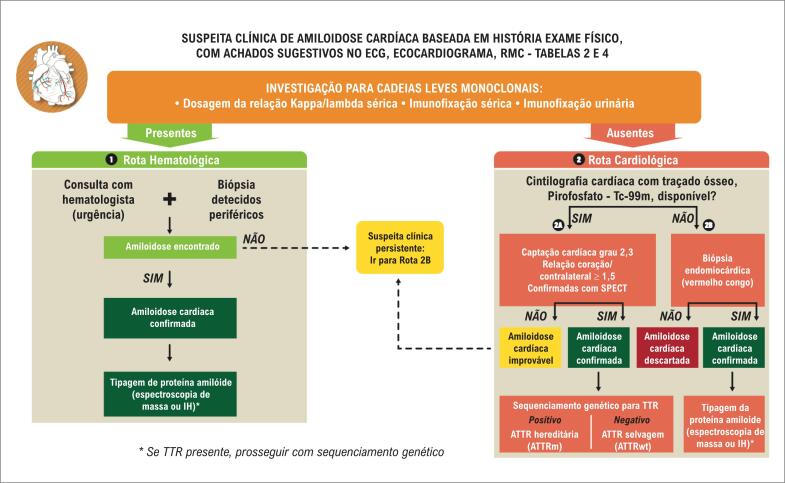
Fluxograma para o diagnóstico de amiloidose cardíaca.

Evidências recentes indicam que a AC, particularmente na forma ATTRwt, é uma condição mais prevalente do que anteriormente se estimava, e que, em parte, isso decorre de um amplo subdiagnóstico e do fato de esta doença mimetizar outras cardiopatias, tais como a miocardiopatia hipertrófica, a ICFEP não amiloide e a estenose aórtica de baixo fluxo e baixo gradiente.^42^ Tais aspectos indicam a necessidade de se assumir uma postura de alta suspeição da presença de doença nos diferentes cenários clínicos, para que se possa desencadear um processo diagnóstico racional.^54^ A Figura 11 traz um fluxograma diagnóstico para AC, cujas etapas são comentadas a seguir. As principais recomendações e classes de evidência para o diagnóstico da AC estão listadas na Tabela 5.

**Tabela 5:** Recomendações para investigação diagnóstica da amiloidose cardíaca

Indicação	Classe de recomendação	Nível de evidência (referências)
Iniciar a investigação diagnóstica para AC em pacientes com sinais e sintomas de IC exibindo um ou mais sinais de alerta da doença (ver Tabela 2)	I	C
Realizar ecocardiograma, com análise da deformação miocárdica (*strain*) do VE sempre que disponível, e/ou RMC com RT e mapa de T1 para pacientes com suspeita de AC	I	B (ref. 60, 61, 62)
Nos casos com alta suspeita de AC, prosseguir a investigação diagnóstica com exames de imunofixação sérica e urinária e dosagem de relação de cadeias leves kappa/lambda, para avaliar a possibilidade da forma AL	I	B (ref. 19)
Realizar cintilografia cardíaca com radiotraçador ósseo,* quando disponível, para diagnóstico de AC por ATTR, após exclusão da forma AL (cadeias leves negativas)	I	B (ref. 19, 94, 95)
Realizar BEM para diagnóstico de AC nos casos com alta suspeita clínica de forma AL (cadeias leves positivas), em que a biópsia de tecido periférico tenha sido negativa	I	B (ref. 108)
Realizar tipagem da proteína amiloide na BEM utilizando-se imuno-histoquímica ou espectroscopia de massa, quando disponíveis, quando o diagnóstico da forma de AC é indeterminado ou discordante com a suspeita clínica	I	C
Realizar BEM para confirmar diagnóstico de AC nos casos suspeitos de ATTR, quando a cintilografia com radiotraçador ósseo não for disponível	I	B (ref. 19)
Realizar BEM para confirmar diagnóstico de AC nos casos com alta suspeita de ATTR, mas com cintilografia com radiotraçador ósseo não diagnóstica	I	C
Realizar sequenciamento genético nos pacientes com diagnóstico de ATTR, para diferenciar ATTRv da ATTRwt	I	B (ref. 33)

* *Radiotraçadores que podem ser utilizados para esta finalidade incluem o ^99m^Tc-pirofosfato, o ^99m^Tc-DPD (ácido 3,3-difosfono-1,2-propanodicarboxílico) e o ^99m^Tc-HMDP (hidroximetilenodifosfonato), sendo que o ^99m^Tc-pirofosfato é o único disponível e aprovado para uso no Brasil.*

A ***etapa preliminar*** e fundamental é o estabelecimento da suspeita clínica, baseada na história clínica, exame físico (ver Tabela 2), com achados sugestivos de AC ao ECG, ecocardiograma e RMC (ver Tabela 4).

***A seguir, em casos com alta suspeita clínica, deve-se proceder a investigação das cadeias leves monoclonais de imunoglobulinas*** para uma triagem efetiva da presença de amiloidose AL, pois o diagnóstico da forma AL é uma urgência médica, devendo-se evitar ao máximo o retardo para o início do tratamento, o que se associa à acentuada piora do prognóstico.

A eletroforese de proteína não é um teste de *screening* adequado, uma vez que o componente monoclonal pode não ser detectado no sangue e/ou urina por esse método. Dessa forma, é importante a realização da imunofixação no sangue e urina, a qual aumenta a sensibilidade de detecção de cadeias leves clonais para em torno de 90%.^103^ A adição da dosagem da relação das cadeias leves livres (*free light chain*), *com detec*ção de uma relação anormal entre as cadeias kappa/lambda (maior que 1,65 ou menor que 0,26), aumenta a sensibilidade de detecção para acima de 99%.^103^,^104^ Portanto, o conjunto dos 3 testes, eletroforese com imunofixação no sangue e urina associada ao teste de dosagem da relação das cadeias leves livres, representa o melhor método não invasivo para detecção de cadeias leves clonais, que indicam a presença de AL.

A detecção de cadeias leves monoclonais torna necessário o encaminhamento do paciente para avaliação com hematologista, seguindo-se a ***rota hematológica*** no fluxograma, e a realização de biópsia tecidual, procedimento fundamental para confirmar o depósito de proteína amiloide e a elaboração da estratégia terapêutica.

### 6.1. Rota Hematológica

#### 6.1.1. Participação do Hematologista e Biópsia de Tecidos Periféricos

O diagnóstico de amiloidose AL dever ser confirmado através de biópsia.

Deve-se dar preferência, inicialmente, à biópsia de gordura abdominal, um método simples e seguro.^105^ A coloração de vermelho-congo com birrefringência sob a luz polarizada é o método usado na determinação de proteína amiloide tecidual.

Em casos de amiloidose sistêmica, em que há acometimento de vários órgãos ou tecidos, a biópsia de gordura abdominal com essas colorações específicas apresenta sensibilidade de 60% a 80% e especificidade de 90% a 100% no diagnóstico de amiloidose,^106^ e tem uma forte associação com a carga corporal total de depósito amiloide.^37^ Contudo, em casos de amiloidose localizada, restrita a um órgão ou tecido, a biópsia de tecido gorduroso subcutâneo é infrequentemente positiva.^107^ Em geral, a positividade da biópsia extracardíaca é maior quando o sitio da biópsia é a gordura abdominal e o tipo de amiloidose é a AL, seguida da ATTRv e, por último, a ATTRwt.^37^ Em uma serie de 131 pacientes com diagnóstico de AC por transtirretina confirmado em biópsia endomiocárdica, a biópsia de gordura abdominal foi positiva em 67% dos pacientes com ATTR hereditária e só 14% dos pacientes com ATTRwt.^8^ Portanto, apesar de a gordura abdominal ser o local inicial preferencial de biópsias extracardíacas, um resultado negativo não deve excluir o diagnóstico, e uma biópsia endomiocárdica deve ser realizada.^37^ Nesses casos, biópsia do órgão afetado oferece sensibilidade e especificidade de 100%.

Vale ressaltar que a coloração vermelho-congo permite a confirmação da infiltração amiloide nos tecidos, porém não identifica o tipo da proteína precursora. Além disso, 40% dos pacientes com ATTR podem ter uma gamopatia monoclonal de significado indeterminado (GMSI), apresentando investigação positiva para cadeias leves monoclonais.^109^

Considerando esses aspectos, para identificação do tipo do depósito amiloide, aspecto fundamental para o tratamento apropriado, torna-se necessária a realização de imuno-histoquímica ou, preferencialmente, de microdissecção a *laser* do material amiloide da biópsia com realização de espectrometria de massa. A imuno-histoquímica permanece o método mais amplamente disponível para identificação do tipo de depósito; contudo, quando o tipo de amiloidose é o de cadeias leves, os resultados não são sempre conclusivos, frequentemente com coexistência de positividade para mais de um tipo de antissoro, geralmente TTR e cadeia kappa ou lambda. A espectrometria de massa tornou-se, portanto, o novo “padrão-ouro” para a identificação do tipo do depósito amiloide.^37^^,^^54^^,^^110^

Perante ***resultados negativos para cadeias leves monoclonais***, situação em que o diagnóstico de ATTR é mais provável, ainda que outras formas mais raras de AC também possam ser diagnosticadas, a investigação deve seguir pela ***rota cardiológica***, tomando-se duas sub-rotas de acordo com a disponibilidade da cintilografia cardíaca com marcadores ósseos.

#### 6.2. Rota Cardiológica

No ***cenário de disponibilidade da cintilografia com marcadores ósseos (sub-rota 2A)***, desde que as cadeias leves monoclonais estejam ausentes, o achado de cintilografia cardíaca mostrando captação acentuada, graus 2 ou 3 da classificação de Perugini (captação cardíaca equivalente ou superior à captação vista nos arcos costais) e relação de captação na área cardíaca em relação à área contralateral do tórax ≥ 1,5, confirmando-se que a captação aumentada encontra-se nas paredes ventriculares por imagens tomográficas de emissão (SPECT), a AC por ATTR está confirmada sem a necessidade de realização de biópsia endomiocárdica. A seguir, o sequenciamento genético da TTR deve ser realizado para definição do caso se ATTR hereditária ou selvagem.

A diferenciação entre ATTR hereditária ou *wild-type* tem implicações prognósticas e terapêuticas, sendo também importante para potencial *screening* familiar e aconselhamento genético.

Na vigência de cintilografia cardíaca com marcadores ósseos negativa e associada à ausência de cadeias leves monoclonais, o diagnóstico de AC é improvável. Contudo, quando a suspeita clínica persiste, baseada principalmente nos resultados de outros métodos de imagem muito sugestivos de amiloidose, a biópsia endomiocárdica tem um papel diagnóstico relevante e deve ser solicitada. Nesse caso, podemos estar diante de ATTRv com algumas mutações com depósitos amiloides que não captam marcador ósseo, como a V30M de início precoce e a P64I, além de outros tipos não usuais de amiloidose.^111^

No cenário de *indisponibilidade da cintilografia com marcadores ósseos (sub-rota 2B)*, a biópsia endomiocárdica também está indicada para elucidar o diagnóstico.

## 7. Prognóstico e Estadiamento

### 7.1. Forma AL

Portadores de AC forma AL exibem depósitos amiloides mais tóxicos e de formação mais acelerada se comparados aos casos pela forma ATTR. Como consequência, aumentos mensais da espessura miocárdica da monta de 1,45 a 2,16 mm podem ser observados associados a elevações mais acentuadas de biomarcadores como troponina e BNP,^112^ bem como ao desenvolvimento de sintomas de insuficiência cardíaca e óbito em média dentro de 6 meses após o diagnóstico.^113^

A somatória desses achados às informações obtidas graças aos recentes avanços tecnológicos e laboratoriais permitiu estabelecer como principal determinante do prognóstico da amiloidose AL a extensão do envolvimento cardíaco pelo material amiloide,^114^ bem como a elaboração de escores de sobrevida. O mais amplamente utilizado é o da Clínica Mayo modificado, exibido na Tabela 6, e que atribui pontos à presença de troponina T ≥ 0,025 ng/mL; NT-ProBNP ≥ 1,800 pg/mL; diferença entre as cadeias leves ≥ 18 mg/dL para indicar indivíduos de mais alto risco.^115^

**Tabela 6:** Estadiamento prognóstico da amiloidose AL, segundo os critérios revisados da Clínica Mayo^115^

Valor de corte dos biomarcadores	Estadiamento (número de biomarcadores com valores elevados)	Sobrevida média
	Estágio I: nenhum biomarcador	94 meses
Troponina-T ≥ 0,025 ng/mL	Estágio II: 1 biomarcador	40 meses
NT-proBNP > 1.800 pg/mL	Estágio III: 2 biomarcadores	14 meses
DCL > 18mg/dL	Estágio IV: 3 biomarcadores	6 meses

*DCL: diferença da dosagem das cadeias leves; NT-proBNP: N-terminal pro-B-type natriuretic peptide*.

Adicionalmente, achados ecocardiográficos, tais como aumento acentuado da espessura das paredes, disfunção diastólica, disfunção do VE, espessamento das valvas e redução do *strain* global longitudinal do VE combinados aos biomarcadores BNP e troponina e *status* hematológico são preditores de maior mortalidade.^116^ Recentemente, uma técnica nova de avaliação miocárdica não invasiva que avalia a rigidez do coração, a elastografia miocárdica, demonstrou boa correlação com a massa ventricular, espessura miocárdica, biomarcadores com BNP, pressões de enchimento, piora de classe funcional e da disfunção diastólica.^117^

A RMC também fornece elementos que se correlacionam com a sobrevida em pacientes com AC.^84^^,^^119^^–^^121^ A presença e a extensão de fibrose miocárdica, detectada pela técnica de realce tardio com gadolínio, são marcadores de mau prognóstico.^118^^–^^119^ Estudo recente mostra que medidas de mapa T1 acima de 1.044ms e do ECV (*extracellular volume*) calculado na fase de equilíbrio do contraste superiores a 0,45 associam-se a aumento da mortalidade cardiovascular de 5,84 e 3,48 vezes, respectivamente.^9^,^10^

Os biomarcadores apresentam não apenas um papel no estadiamento desses doentes, mas também possibilitam avaliação da resposta ao tratamento, que na prática clínica é sempre um desafio, principalmente para a forma AL, uma vez que a quimioterapia pode ser um fator adicional para lesão miocárdica. O que se tem bem estabelecido na literatura é a concordância entre redução dos níveis de NT-proBNP e resposta hematológica ao tratamento, assim como melhora de classe funcional NYHA. O inverso também é verdadeiro, como o aumento dos níveis de NT-proBNP, troponina e redução da fração de ejeção. (ver Tabela 9 – tópico sobre tratamento da AC forma AL)

### 7.2. Forma ATTR

A AC por ATTR exibe um perfil evolutivo mais benigno e de maior sobrevida se comparado à forma AL.^121^ Além do tipo específico de depósito amiloide, outros fatores como genótipo, dados clínicos, biomarcadores laboratoriais e achados radiológicos fornecem instrumentos que auxiliam o planejamento terapêutico e permitem inferir o prognóstico.^13^

Nos casos de ATTR hereditária, o perfil genético determina o fenótipo clínico e a evolução da doença, ainda que a penetrância do gene possa variar em diferentes populações. Nesse sentido, variantes patogênicas de acometimento predominantemente miocárdico como a V122I apresentam sobrevida menor comparativamente à ATTRwt e às variantes mistas ou de predomínio neuropático como a V30M.^20^^,^^122^^,^^123^

Do ponto de vista clínico, o surgimento de sintomas de insuficiência cardíaca e a duração dos sintomas apresentam valor prognóstico.^124^ Observa-se uma redução da sobrevida quanto mais prolongada a descompensação clínica e quanto mais avançada a classe funcional da NYHA.^125^,^126^ As sobrevidas medianas observadas para cada classe funcional foram: I = 4,6 anos, II = 4,1 anos, III = 2,1 anos e IV = 1,3 ano. Apesar de o gênero, a idade ao diagnóstico e a presença de comorbidades como valvopatia aórtica ou taquiarritmias não demonstrarem efeito direto sobre a mortalidade, uma maior associação com desfechos cardíacos compostos desfavoráveis é observada em pacientes idosos do sexo masculino e com doenças associadas.^127^

Os resultados de exames laboratoriais de baixo custo, de realização rápida e de fácil interpretação têm sido usados em diferentes propostas para estadiamento da doença, obtendo bom nível de correlação com a expectativa de vida (Tabela 7).^128^^–^^130^ Os níveis séricos de troponinas T e I, do NT-proBNP e a taxa de filtração glomerular refletem a toxicidade dos depósitos amiloides nos órgãos-alvos tanto por ação direta quanto por ativação de processos de estresse oxidativo e inflamação.^131^ As recomendações para estratificação de risco/estadiamento da AC e monitoramento da resposta ao tratamento são mostradas na Tabela 8.

**Tabela 7:** Propostas de estadiamento da amiloidose ATTR utilizando biomarcadores

Proposta de estadiamento	Forma de AC	Ponto de corte dos biomarcadores	Estadiamento	Sobrevida média (meses)
Grogan et al. (2016)	ATTRwt	NT-proBNP > 3.000 ng/L Troponina-T ≥ 0,05 µg/L	Estágio I NT-proBNP < 3.000 ng/L TncT < 0,05 µg/L	Estágio I = 66
			Estágio II NT-proBNP **ou** TncT acima do ponto de corte	Estágio II = 40
			Estágio III NT-proBNP **e** TncT acima do ponto de corte	Estágio III = 20
Gillmore et al. (2018)	ATTRwt ATTRv	NT-proBNP > 3.000 ng/L eGFR < 45 mL/min	Estágio I NT-proBNP < 3.000ng/L eGFR ≥ 45mL/min	Estágio I = 69,2
			Estágio II NT-proBNP > 3.000 ng/L **ou** eGFR < 45 mL/min	Estágio II = 46,7
			Estágio III NT-proBNP > 3.000 ng/L **e** eGFR < 45 mL/min	Estágio III= 24,1

*ATTRwt: amiloidose ATTR forma selvagem; ATTRv: amiloidose ATTR forma hereditária; NT-proBNP: N-terminal pro-B-type natriuretic peptide; eGFR: taxa de filtração glomerular estimada; FEVE: fração de ejeção ventricular esquerda.*^11^.

**Tabela 8:** Recomendações para a estratificação de risco/estadiamento da amiloidose cardíaca (AC) e monitoramento da resposta ao tratamento e/ou progressão da doença

Indicação	Classe de recomendação	Nível de evidência (referências)
Estratificar o risco dos pacientes com AC utilizando-se sistemas de estadiamento validados, incluindo biomarcadores como BNP/NT-ProBNP e/ou troponinas	I	B (ref. 128-130)
Monitorar a progressão da AC e/ou resposta ao tratamento específico utilizando-se ecocardiograma ou ressonância magnética cardíaca e biomarcadores*	I	C

* *A periodicidade da avaliação depende da forma da AC e da evolução clínica.*

Achados em exames de imagem, como o ecocardiograma, a cintilografia cardíaca com Tecnécio-pirofosfato (^99m^Tc-PYP) e a RMC, fornecem dados complementares que auxiliam na estratificação prognóstica.^9^^,^^80^^,^^94^^,^^132^^–^^134^ (12-17). (Tabela 9). Além disso, a crescente qualidade das imagens obtidas, associada ao maior detalhamento quantitativo dos depósitos amiloides, diminuiu a necessidade de realização de biópsia miocárdica como elemento prognosticador de doença.^9^

**Tabela 9:** Parâmetros de exames de imagem com valor prognóstico na amiloidose cardíaca ATTR

Ecocardiograma	Cintilografia com^99m^Tc-pirofosfato	Ressonância magnética cardíaca
↓ FEVE	Relação C/CL ≥ 1,6	TAPSE
↓ FCM		↓ Volume de ejeção indexado
↓ SGL		↑ Realce tardio com gadolínio
*Apical Sparing*		↑ Volume extracelular
↓ *Stroke* Volume_I_^(17)^		↑ T1 nativo

*FEVE: fração de ejeção do ventrículo esquerdo; FCM: fração de contração miocárdica (FCM = VS/VOL VE); SGL: strain global longitudinal; SV_I_: índice de volume sistólico; C/CL: relação coração e região torácica contralateral; TAPSE: excursão sistólica do anel tricúspide*.

## 8. Tratamento

O tratamento da AC compreende medidas específicas direcionadas para reduzir ou evitar a progressão dos depósitos de fibrilas amiloides, sendo abordadas neste texto separadamente, para as formas AL e ATTR. Além destas, são também necessárias medidas gerais de manejo das anormalidades clínicas e hemodinâmicas causadas pela doença, incluindo a insuficiência cardíaca e os distúrbios do ritmo cardíaco, sendo aplicáveis para ambas as formas, AL e ATTR.

### 8.1. Princípios da Terapia Específica da Amiloidose AL

A amiloidose AL é uma doença causada pela produção aberrante da cadeia leve da imunoglobulina (*kappa* ou *lambda*). Com isso, a base do tratamento específico consiste em eliminar a sua produção por meio da erradicação do clone de plasmócitos na medula óssea. A redução rápida e, idealmente, normalização dos níveis de cadeias leves são alguns dos principais objetivos no tratamento da amiloidose AL, sendo denominada resposta hematológica. Já a reversão da lesão nos tecidos e órgãos acometidos pelo depósito amiloide é chamada de resposta orgânica, e compreende o segundo maior objetivo do tratamento. Outros objetivos do tratamento são melhorar a qualidade de vida e a sobrevida geral.^135^

Após confirmado o diagnóstico de amiloidose sistêmica AL, o plano terapêutico deve ser definido pelo hematologista. No entanto, a identificação e o manejo das disfunções orgânicas são essenciais, sendo imprescindível o trabalho multidisciplinar, incluindo acompanhamento conjunto com o cardiologista e outros especialistas (Tabela 12). O comprometimento cardíaco é o principal fator prognóstico na amiloidose AL, determinando não só a sobrevida, mas também a tolerabilidade aos tratamentos citotóxicos. O impacto do comprometimento cardíaco na sobrevida é bem estabelecido na literatura, e sistemas de estadiamento já validados auxiliam na estratificação de risco do paciente, sendo utilizados em conjunto com outros parâmetros para guiar diferentes estratégias terapêuticas.^135^

A sobrevida na amiloidose AL está relacionada à produção de cadeias leves amiloidogênicas e à lesão de órgão-alvo, principalmente o coração. Com isso, o estadiamento inclui biomarcadores que estão relacionados a essas ocorrências,^104^^,^^115^^,^^136^ conforme descrito em tópico anterior (ver Tabela 5).

O valor dessa estratificação prognóstica foi observado tanto em indivíduos tratados com transplante de células-tronco hematopoiéticas (TCTH) autólogo quanto naqueles tratados sem esse procedimento. A sobrevida mediana segundo essa estratificação prognóstica foi de 55, 19, 12 e 5 meses em pacientes que tiveram ao diagnóstico estágios I, II, III e IV, respectivamente, e que não receberam ou tiveram falha no TCTH e 97, 58 e 22 meses, para os estágios II, III e IV, respectivamente, naqueles submetidos ao TCTH.^115^

Pacientes avaliados como baixo risco são candidatos à terapia com quimioterapia (QT) em altas doses seguidas de TCTH autólogo (Tabela 12). Atualmente, esta é considerada a estratégia mais eficaz para erradicação do clone de plasmócitos.^135^ Pacientes de risco intermediário geralmente recebem QT em doses convencionais e, caso apresentem melhora clínica e laboratorial, podem se tornar candidatos ao TCTH autólogo. Por último, pacientes idosos, mais frágeis, com comprometimento de mais de um órgão ou com cardiomiopatia avançada são tratados com QT em doses ajustadas, representando um grupo de prognóstico desfavorável, uma vez que geralmente não toleram o tratamento.^135^

Após 3 meses do término do tratamento, as respostas hematológica e orgânica devem ser avaliadas com exames específicos relacionados à detecção de gamopatia monoclonal e comprometimento de cada órgão acometido no diagnóstico. Os critérios de resposta hematológica e orgânica estão resumidos nas Tabelas 10 e 11.^136^,^137^

**Tabela 10:** Critérios de resposta hematológica ao tratamento

**Resposta completa (RC):** normalização das cadeias leves livres, imunofixação no sangue e urina negativa (sem presença de componente clonal)
**Resposta parcial muito boa:** redução na diferença entre cadeia leve livre envolvida e não envolvida para < 40 mg/L
**Resposta parcial (RP):** redução > 50% na diferença entre cadeia leve livre envolvida e não envolvida
**Sem resposta:** não obtenção de uma RP
**Doença progressiva:** aumento de cadeia leve livre em 50% ou para > 100 mg/L. Em pacientes que obtiveram uma RC, reaparecimento de um componente clonal na imunofixação ou nas cadeias leves livres. Em pacientes que obtiveram uma RP, aumento em 50% do componente monoclonal no sangue ou urina

**Tabela 11:** Critérios de resposta órgão-específica ao tratamento

**Resposta cardíaca:** redução > 30% e > 300ng/L em pacientes com NT-proBNP ≥ 650 ng/L ou melhora funcional de pelo menos duas classes de acordo com a *New York Heart Association*
**Resposta renal:** diminuição da proteinúria ≥ 50% (redução de ao menos 0,5g/24h) em 6 meses sem piora do eGFR em ≥ 25%. Análises retrospectivas recentes sugerem que redução ≥ 30% na proteinúria está associada a melhor prognóstico, mas esse critério (de ≥ 30%) ainda não foi incorporado em recomendações formais^18^
**Resposta hepática:** redução em fosfatase alcalina em pelo menos 50% e redução da dimensão do fígado em pelo menos 2 cm
**Sistema nervoso periférico:** melhora da velocidade de neurocondução pela eletroneuromiografia

Mesmo alcançando resposta hematológica completa, a melhora das disfunções se dá mais lentamente, podendo ocorrer até meses a anos após a normalização das cadeias leves.^19^

#### 8.1.1. Tratamento de Pacientes Elegíveis ao Transplante Autólogo de Células-tronco Hematopoiéticas (TCTH)

Apenas 20% dos pacientes diagnosticados com amiloidose AL apresentam condições clínicas para o TCTH, e o número de pacientes elegíveis pode aumentar se houver resposta orgânica após esquemas de indução contendo terapias antiplasmocitárias eficazes (bortezomibe, daratumumabe).^135^^,^^139^^,^^140^

A avaliação de elegibilidade ao TCTH é ponto-chave para se obter sucesso terapêutico com essa estratégia. Um único estudo prospectivo randomizado comparou o TCTH com QT convencional, não havendo benefício de sobrevida global deste em relação à estratégia menos intensiva. Neste estudo, porém, a alta taxa de mortalidade associada ao TCTH (24%) foi relacionada aos critérios de inclusão, inexperiência do centro transplantador e uso de dose subterapêutica de melfalano.^141^

Uma vez que não há critérios precisos bem estabelecidos e validados prospectivamente na literatura, cada centro estabelece seus próprios critérios de elegibilidade ao TCTH. Embora a avaliação subjetiva seja relevante, alguns fatores podem guiar a indicação do TCTH: “idade fisiológica” ≤ 70 anos, ClCr > 30 mL/min (exceto aqueles em diálise crônica), troponina T < 0,06 ng/mL, escala de performance ECOG (Eastern Cooperative Oncology Group) ≤ 2, classe funcional da *New York Heart Association* I ou II e pressão arterial sistólica > 90 mmHg. A adequada estratificação de risco pré-tratamento concomitante ao desenvolvimento de melhores condições de suporte tais como antibioticoterapia e terapia intensiva tem levado à redução da mortalidade associada ao TCTH nas últimas décadas, com taxas de 2,4% a 3,4% em análises retrospectivas.^142^^–^^148^

Dessa forma, o TCTH deve ser recomendado como terapia de primeira linha nos indivíduos classificados como elegíveis (Tabela 12). Essa recomendação se deve à obtenção de altas taxas de resposta hematológica, levando à redução da produção e potencial reabsorção do amiloide, com consequente melhora da disfunção orgânica, *performance status,* aumento da sobrevida e qualidade de vida. Como exemplo, em uma série de 672 pacientes com amiloidose AL submetidos a TCTH, 84% apresentaram resposta hematológica, sendo 39% de RC, e a sobrevida alcançada foi > 50% em 15 anos.^146^^,^^147^^,^^149^

**Tabela 12:** Recomendações para tratamento da amiloidose cardíaca (AC)

Recomendação	Classe de recomendação	Nível de evidência (referências)
**Recomendações para o tratamento da insuficiência cardíaca**		
Diuréticos de alça para controle das manifestações congestivas	I	C
Evitar uso de substâncias bradicardizantes, salvo situações especiais	I	C
Uso rotineiro dos fármacos modificadores do prognóstico utilizados para o tratamento da ICFER: betabloqueador, iECA, BRA, INRA, iSGLT2, ARM	III	C
Anticoagulação oral para pacientes com AC e fibrilação atrial, independentemente do risco calculado de AVE ou embolia sistêmica	I	C
Oferecer aos pacientes com diagnóstico confirmado de AC o encaminhamento para um centro especializado	I	C
**Recomendações para o tratamento específico para ATTR**		
Tafamidis 80mg/dia, para o tratamento específico de pacientes com AC confirmada por ATTRv ou ATTRwt, com insuficiência cardíaca, em CF I a III da NYHA, sem disfunção renal grave, para redução da mortalidade e redução da taxa de progressão da incapacidade física e da perda da qualidade de vida	I	B (ref. 162, 163)
**Recomendações para o tratamento específico para AL**		
Avaliação conjunta com hematologista nos casos suspeitos ou confirmados de AL, para definição do tratamento:		
Considerar o transplante de células-tronco hematopoiéticas (TCTH), para pacientes com AL classificados como elegíveis: não exibindo comorbidades graves, sem doença cardíaca avançada, sem insuficiência renal grave, sem envolvimento de mais de dois órgãos ou idade avançada	I	B (ref. 147, 148, 149)
Iniciar terapia quimioterápica com medicações antiplasmocitárias para pacientes com AL não candidatos ao TCTH.	I	A (ref. 139,156)

*CF: classe funcional; NYHA: Sociedade Nova-iorquina de Cardiologia; AVE: acidente vascular encefálico; iECA: inibidor da enzima de conversão da angiotensina; BRA: bloqueador do receptor da angiotensina-II; iSGLT2: inibidor do cotransportador de sódio-glicose-2; ARM: antagonista do receptor de mineralocorticoide*.

O TCTH pode ser realizado logo após diagnóstico (*upfront*) em pacientes com < 10% de plasmócitos clonais na medula óssea ou ser precedido de terapia de indução com esquemas contendo bortezomibe e/ou anticorpo monoclonal anti-CD38. Esse último cenário deve ser considerado quando há associação com mieloma múltiplo, atraso previsível para realização do TCTH, baixa *performance* que pode melhorar com a terapia de indução e presença de > 10% de infiltração por plasmócitos clonais na medula óssea, a qual está associada a pior prognóstico.^135^^,^^150^

Os principais esquemas utilizados como terapia pré-transplante são ciclofosfamida, bortezomibe e dexametasona (CyBorD) e daratumumabe, bortezomibe, ciclofosfamida e dexametasona (Dara-CyBorD).^139^

Dado o novo cenário de surgimento de novas terapias no tratamento da amiloidose AL e a escassez de estudos randomizados consolidando o papel do transplante, serão necessários novos estudos para entender se o TCTH permanecerá sendo a terapia mais eficaz nas próximas décadas.

#### 8.1.2. Tratamento de Pacientes Não Elegíveis ao TCTH/Quimioterapia Convencional

A maior parte dos pacientes diagnosticados com amiloidose AL não é elegível à terapia de maior intensidade com TCTH autólogo devido a comorbidades, como doença cardíaca avançada, insuficiência renal, envolvimento de mais de dois órgãos ou idade avançada. Dessa forma, nesse grupo de pacientes, terapias quimioterápicas com medicações antiplasmocitárias são a base do tratamento (Tabela 12). Os esquemas utilizados são, em sua maioria, com base naqueles empregados para o mieloma múltiplo.

O esquema combinando ciclofosfamida, bortezomibe e dexametasona (CyBorD) demonstrou-se altamente eficaz (resposta hematológica 81% a 94%), bem tolerado e capaz de gerar resposta hematológica rápida (em até 3 meses) em dois estudos retrospectivos com pequeno número de pacientes (n = 17 e 43). A sobrevida global em 2 anos chegou a 92% nesses estudos.^151^^,^^152^ A maior casuística com este esquema incluiu 230 pacientes, com 60% de resposta hematológica, sendo 23% de resposta completa. Entretanto, resposta orgânica cardíaca e renal foi observada em somente 17% e 25% dos pacientes, respectivamente. Dentre os pacientes com cardiopatia avançada, menor taxa de resposta hematológica foi observada (42% global e 14% completa), sendo a mediana de sobrevida global de 7 meses para este grupo ^153^ A possibilidade do CyBorD de tornar pacientes inicialmente inelegíveis ao TCTH autólogo em elegíveis (após resposta hematológica e melhora clínica) é um ponto importante a ser considerado no desenvolvimento do plano terapêutico.^153^^–^^155^

Recentemente, dois estudos randomizados instituíram combinações de QT como possíveis novos tratamentos de escolha em pacientes com amiloidose AL não elegíveis a TCTH. O primeiro comparou o esquema bortezomibe, melfalano e dexametasona (BMD) a MD, demonstrando benefício da adição do bortezomibe a MD com maior taxa de resposta hematológica global após 3 meses (79% *vs.* 52%), resposta orgânica cardíaca (38% *vs.* 28%) e melhora de sobrevida global, com redução de 2 vezes na taxa de mortalidade.^156^ O maior estudo randomizado em amiloidose AL (andromeda) associou um anticorpo monoclonal anti-CD38 (daratumumabe) ao esquema CyBorD, com resultados promissores.^139^ Resposta hematológica global de 92% foi alcançada no grupo Dara-CyBorD *versus* 77% no grupo-controle que recebeu CyBorD sem daratumumabe, com RC de 53% *versus* 18%, e mediana de 2 meses para atingir esta resposta. Melhora de função orgânica foi também observada no grupo que recebeu o anticorpo monoclonal, com melhora da sobrevida livre de progressão. É importante ressaltar que pacientes com cardiopatia avançada (classificados como estadiamento IIIb) foram excluídos de ambos os estudos randomizados citados, sendo que o tratamento para este grupo permanece um desafio na prática clínica.

### 8.2. Terapias Específicas da ATTR

**Figura 12:**
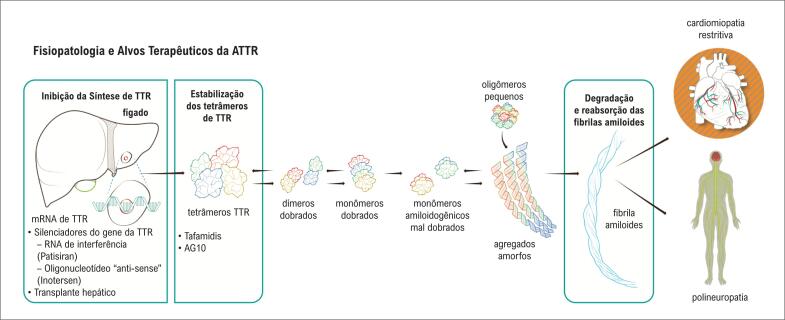
Ilustração mostrando a fisiopatogênese da deposição de fibrilas amiloides na ATTR e alvos terapêuticos reconhecidos.

Várias etapas do processo fisiopatogênico de formação e deposição das fibrilas amiloides no tecido cardíaco são potenciais alvos terapêuticos na ATTR, como ilustrado na Figura 12, incluindo-se: 1) transplante hepático; 2) estabilizadores dos tetrâmeros da TTR; 3) inibidores da síntese hepática de TTR; e 4) degradação e reabsorção das fibrilas amiloides depositadas.

#### 8.2.1. Transplante Hepático

O transplante hepático foi, no passado, proposto como tratamento curativo potencial para pacientes com polineuropatia associada à ATTRv.^157^ O transplante hepático, ao remover a fonte de produção das moléculas mutadas de TTR, associa-se a aumento da sobrevida, com relatos de taxa de sobrevida em 20 anos de 55,3%. Contudo, a deposição de TTR pode continuar após o transplante hepático e associar-se à progressão da cardiopatia, provavelmente porque a presença de fibrilas amiloides já acumuladas no miocárdio promove deposição adicional de TTR selvagem ao longo do tempo.^158^^,^^159^ Dessa forma, transplante duplo de coração e fígado pode ser possível e parece associado a melhor prognóstico em comparação a transplante de um dos órgãos isoladamente.^160^ Considerando a reduzida disponibilidade de órgãos e centros de transplante, além dos riscos representados pela imunossupressão ao longo da duração da vida, o desenvolvimento de novas terapias capazes de bloquear a síntese hepática de TTR deve substituir o transplante hepático como alternativa de tratamento para suprimir a produção de TTR.

#### 8.2.2. Estabilizadores Seletivos dos Tetrâmeros da TTR

##### 8.2.2.1. Tafamidis

Tafamidis é uma molécula pequena que inibe seletivamente a dissociação dos tetrâmeros de TTR ao ligar-se aos sítios de ligação da tiroxina (T4) e, assim, inibindo efetivamente a cascata que resulta na formação das fibrilas amiloides.^161^ Quando testado em estudo clínico fase 3, tafamidis se mostrou eficaz em reduzir a progressão de manifestações neurológicas em pacientes em estágios precoces da polineuropatia por ATTRh.^162^

Tafamidis é o único fármaco, até o momento, que foi especificamente testado em pacientes com AC em um estudo clínico multicêntrico prospectivo, randomizado e placebo controlado, o estudo ATTR-ACT.^163^ Este estudo fase 3 incluiu 441 pacientes (18 a 90 anos de idade) com diagnóstico de cardiomiopatia por ATTR hereditária ou selvagem, caracterizada por história de insuficiência cardíaca, espessura do septo interventricular > 12 mm ao ecocardiograma, demonstração de depósitos amiloides por TTR (biópsia ou cintilografia com marcadores ósseos positiva), NT-Pró-BNP > 600 pg/mL, distância percorrida no teste de caminhada de 6 minutos (TC6m) > 100 metros. Os principais critérios de exclusão foram: classe funcional IV da NYHA, presença de amiloidose AL, TFG < 25mL/min/1,73 m^2^. Os pacientes foram randomizados para receber tafamidis na dose de 80 mg/dia, tafamidis 20 mg/dia ou placebo nas proporções de 2:1:2. O desfecho primário do estudo avaliou de forma hierárquica mortalidade por qualquer causa, seguida pela frequência de hospitalização cardiovascular ao longo de 30 meses de seguimento. Os desfechos secundários principais foram a mudança da distância percorrida no TC6m e do escore de qualidade de vida obtido no *Kansas City Cardiomyopathy Questionnaire* (KCCQ). Para análise estatística, agruparam-se os resultados dos grupos de pacientes recebendo as doses de tafamidis de 80mg e 20mg. Os resultados reportados mostraram que os pacientes recebendo tafamidis (n=264) exibiram redução de 30% no risco relativo de mortalidade por qualquer causa quando comparados aos pacientes recebendo placebo (n = 177) [(RR = 0,70 [IC 95%: 0,51 – 0,96]), 32% na redução de internações cardiovasculares (RR = 0,68 [IC95%: 0,56 – 0,81]) e redução da taxa de declínio da distância percorrida no TC6m (p < 0,001) e menor taxa de declínio do escore do KCCQ (p < 0,001). As curvas de sobrevida de Kaplan-Meier mostraram que tafamidis resultou em redução da mortalidade por qualquer causa, com as curvas divergindo após cerca de 18 meses de tratamento, resultado concordante com o conceito de que tafamidis é um fármaco modificador da história natural da doença. A substância foi bem tolerada, com incidência similar dos diferentes efeitos adversos nos grupos tafamidis e placebo. Na análise de subgrupos de pacientes, tafamidis associou-se à redução da mortalidade por qualquer causa em comparação ao placebo de maneira independente à classe funcional da NYHA, ou genótipo da ATTR, se hereditária ou selvagem. Contudo, os pacientes exibindo classe funcional III da NYHA no momento da inclusão no estudo, alocados no grupo tafamidis, apresentaram maiores taxas de hospitalização quando comparados aos pacientes recebendo placebo, resultado provavelmente explicado por uma sobrevida mais longa durante uma fase de doença mais grave. Essa análise de subgrupos levanta a necessidade de um estudo com tamanho amostral adequado para avaliar especificamente o efeito do tafamidis em pacientes com ATTR e sintomas mais avançados de insuficiência cardíaca.

Mais recentemente, foram publicados os resultados do estudo de extensão e aberto do estudo ATTRACT mostrando que a dose de 80 mg/dia de tafamidis em comparação à dose de 20 mg/dia associou-se a aumento significativo da sobrevida [RR = 0,70 (IC 95%: 0,50 – 0,979)], p = 0,0374.^164^

Com base nessas evidências, recomenda-se o uso de tafamidis na dose de 80mg/dia para tratamento de pacientes com AC por ATTRv ou ATTRwt, para pacientes com insuficiência cardíaca, em CF I a III da NYHA, sem disfunção renal grave, devendo-se iniciar a terapia nas fases mais precoces da doença (Tabela 12). No Brasil, tafamidis na dose de 80mg/dia recebeu a aprovação da Anvisa para tratamento da AC por ATTR.

##### 8.2.2.2. AG10

AG10 é um estabilizador seletivo dos tetrâmeros da TTR, desenhado para mimetizar a influência estrutural da mutação protetora superestabilizadora T119M, que reduz significativamente a taxa de dissociação dos tetrâmeros.^165^ Essa droga foi avaliada em estudo multicêntrico fase 2, randomizado, duplo cego, placebo-controlado envolvendo 49 pacientes com cardiomiopatia por ATTR sintomáticos, em classe funcional II ou III da NYHA. O tratamento se mostrou bem tolerado e demonstrou estabilização da TTR quase completa.^166^ Um estudo clínico multicêntrico fase 3 testando o efeito do AG10 em pacientes com AC encontra-se em andamento. (ClinicalTrial.gov – Identificador: NCT03860935).

#### 8.2.3. Inibidores da Síntese Hepática de TTR

Terapias baseadas no silenciamento da expressão dos genes que codificam a produção hepática de TTR são muito promissoras, incluindo estratégias com RNA de interferência (Patisiran) e oligonucleotídeos *anti-sense* (Inotersen). Ambos os fármacos foram testados em estudos multicêntricos fase 3 em pacientes com polineuropatia por ATTRv e se mostraram efetivos em reduzir a progressão das manifestações neurológicas.^167^^,^^168^ Análises *post hoc* de subgrupos de pacientes com AC incluídos nesses estudos sugerem efeitos positivos sobre a progressão da cardiomiopatia. Ambas as classes de silenciadores de expressão gênica estão atualmente sendo testadas em estudos multicêntricos fase 3 em pacientes com diagnóstico de AC-ATTR (ClinicalTrials.gov Identifiers: NCT03997383 e NCT04136171).

#### 8.2.4. Degradação e Reabsorção das Fibrilas Amiloides

Alguns compostos com base em moléculas hidrofóbicas mostraram-se efetivos em estudos *in vitro* e experimentais para promover a desagregação das fibras amiloides depositadas nos tecidos, permitindo a reabsorção dos seus depósitos pelo sistema macrofágico.^169^ A doxiciclina, um antibiótico da família das tetraciclinas, mostrou ser efetiva para esta finalidade em estudos experimentais,^170^ com efeitos sinérgicos quando empregada junto com o ácido tauro-urso-desoxicólico.^171^ Ainda que esta abordagem se mostre promissora quando são contemplados os estudos pré-clínicos, a experiência clínica com uso desta combinação de medicamentos é muito limitada e não permite estabelecer sua eficácia ou embasar recomendações para seu uso.

## 9. Manejo da Síndrome da Insuficiência Cardíaca

Além da terapia específica para amiloidose, pode ser necessário tratamento de suporte para IC. A AC apresenta-se inicialmente como IC de fração de ejeção preservada (ICFEp) e padrão restritivo do enchimento ventricular esquerdo, podendo ocorrer, tardiamente, a progressão da doença e redução da fração de ejeção. Esse mecanismo fisiopatológico pode explicar as dificuldades de manejo clínico do paciente com amiloidose cardíaca ao se usar medicações consagradas para IC de fração de ejeção reduzida (ICFEr).^20^^,^^21^

A manutenção da euvolemia, com restrição hídrica e medicamentos, é o foco. A medicação mais utilizada é o diurético de alça, indicado para reduzir a congestão pulmonar e sistêmica, podendo associar-se os antagonistas da aldosterona (Tabela 12). O uso de diuréticos buscando a euvolemia pode ser um desafio, visto que o excesso pode prejudicar a função renal e/ou resultar em baixo débito cardíaco devido à redução da pré-carga em um coração com volume sistólico ejetado já reduzido.^172,173^ Além disso, em pacientes com polineuropatia autonômica, a presença de hipotensão pode dificultar o uso de diuréticos devido à pré-carga lábil.^172^

Em relação aos fármacos modificadores de prognóstico usados para o tratamento da ICFER, os antagonistas neuro-hormonais como inibidores da enzima de conversão da angiotensina II (iECA), bloqueadores dos receptores de angiotensina II (BRA) e betabloqueadores (BB), ou até mesmo substâncias recentemente descritas como os inibidores da neprilisina e antagonistas do receptor de angiotensina II (INRA) e inibidores de SGLT2, não há evidências científicas de benefício na amiloidose cardíaca, além de haver risco de hipotensão associada à disfunção autonômica. Aimo et al.^173^ observaram, em um estudo retrospectivo, unicêntrico com 99 pacientes com AC (33% com amiloidose AL e 67% com amiloidose TTR) que iECA/BRA e antagonistas mineralocorticoides (AMR) podem ser utilizados com segurança, com ajuste gradual da dose, se ausência de contraindicações, e que BB são menos tolerados em pacientes com amiloidose AL, disfunção de ventrículo esquerdo e direito. O uso de BB e bloqueadores de cálcio (BCC) não di-hidropiridínicos normalmente não é bem tolerado, pois, devido ao volume sistólico ejetado baixo, esses pacientes dispõem apenas da frequência cardíaca para manutenção do débito cardíaco. Além disso, o uso de BCC não di-hidropiridínicos em amiloidose por cadeias leves deve ser evitado, pois eles se ligam às fibrilas amiloides, podendo resultar em bloqueios avançados e choque cardiogênico.^172^^,^^173^

### 9.1. Manejo das Arritmias

As arritmias são muito comuns em pacientes com amiloidose cardíaca, e geralmente são sintomáticas e pouco toleradas. A avaliação de arritmias nesta população deve envolver três diferentes situações: arritmias atriais; arritmias ventriculares e doença do sistema de condução.

#### 9.1.1. Arritmias Atriais e Anticoagulantes

O depósito amiloide leva a espessamento atrial com alteração de relaxamento atrial, aumento das pressões intracavitárias, gerando dilatação atrial que, em associação à fibrose atrial, predispõe à fibrilação atrial (FA) ou a outras arritmias atriais. A prevalência de FA pode variar de 11% a 71% em pacientes com AC, sendo ainda mais elevada em pacientes com AC por ATTR, possivelmente por acometer pacientes mais idosos do sexo masculino.^174^^,^^175^

O manejo da FA nesses pacientes é, em geral, difícil, uma vez que costumam não tolerar os principais fármacos utilizados para o controle de frequência, tais como BB, BCC ou digitálicos, por precipitarem hipotensão postural e descompensação da IC. Diante da necessidade de uso dessas medicações, aconselha-se uso de baixas doses com monitoramento hemodinâmico cauteloso, além de controle de nível sérico, se digoxina for utilizada. Para o controle de ritmo, uma análise retrospectiva não mostrou diferença em relação à sobrevida quando comparados pacientes que receberam fármacos antiarrítmicos com pacientes que foram tratados apenas com controle de frequência.^176^ Dados recentes sugerem que a ablação por cateter pode se associar à redução de mortalidade em pacientes com AC, especialmente quando realizada precocemente. No entanto, esses dados são oriundos de estudos observacionais retrospectivos pequenos que também demostraram alta taxa de recorrência de FA.^177^

Por fim, a redução da contratilidade atrial provocada pela infiltração amiloide no tecido atrial pode ainda contribuir para a formação de trombos, dado este demonstrado em estudos de autópsia que revelam a presença de até 33% de trombos intracavitários em pacientes com AC^178^ e estudos retrospectivos revelando prevalência de trombo intracavitário de 15% a 33%.^179^^,^^180^ Diante disso, a anticoagulação está indicada em pacientes com AC que desenvolvam FA, independentemente dos cálculos de escores de risco (Tabela 12). Além disso, trombo atrial esquerdo tem sido descrito em até 30% dos pacientes submetidos ao ecocardiograma transesofágico realizado antes de uma cardioversão elétrica programada mesmo em vigência de anticoagulação adequada.^181^^,^^182^ Assim, recomenda-se a realização de ecocardiograma transesofágico em todo paciente candidato à cardioversão elétrica. O papel da anticoagulação em pacientes em ritmo sinusal ainda é incerto. Porém, mesmo na presença de ritmo sinusal, a alteração da contratilidade atrial é comum e está associada a formação de trombo atrial, especialmente em pacientes com amiloidose AL.^180^^,^^182^

#### 9.1.2. Arritmias Ventriculares

Arritmias ventriculares são frequentes em pacientes com AC, especialmente amiloidose AL. Estudos prévios têm detectado a presença de arritmias ventriculares complexas em pelo menos 50% dos pacientes com AC forma AL, sendo taquicardia ventricular não sustentada (TVNS) a arritmia mais frequente e associada a menor sobrevida.^183^^,^^184^

Neste contexto, discute-se o papel do uso de cardiodesfibrilador implantável (CDI) na prevenção de morte súbita de pacientes com AC. Na presença de taquicardia ventricular instável ou sobreviventes de parada cardíaca sem causa reversível com expectativa de vida maior que 1 ano com qualidade significativa, sugere-se potencial benefício do uso de CDI.^185^^–^^187^

No entanto, como prevenção primária, a indicação de CDI é mais difícil por diferentes motivos. O primeiro deles é que a maioria das causas de morte súbita descritas relaciona-se à dissociação eletromecânica e não arritmias ventriculares;^188^ o segundo é a histórica baixa expectativa de vida de pacientes com AC, especialmente AL, e, por fim, as ferramentas de estratificação de risco tradicionais, tais como fração de ejeção reduzida, não parecem de fato aplicáveis a pacientes com AC, uma vez que disfunção sistólica grave está associada a estágios finais da doença em que predomina a falência de bomba como causa de morte. O desafio é, portanto, identificar o paciente em risco na fase precoce da doença quando arritmia predomina e pode potencialmente ser corrigida pelo uso do CDI. Estudos prospectivos são necessários para definir quando a indicação de CDI pode ser benéfica em pacientes com AC.^189^

### 9.2. Distúrbios de Condução

Doença do sistema de condução é altamente prevalente entre pacientes com AC, sendo a condução atrioventricular mais comumente afetada do que o nó sinusal. A fisiopatologia envolvida não está totalmente definida, embora estudos pequenos sugiram envolvimento do sistema de condução por proteína amiloide.^190^ Há evidências de que essas alterações são a causa de óbito em um número considerável de pacientes com amiloidose cardíaca,^191^ sendo o marca-passo (MP) frequentemente indicado, especialmente em pacientes com AC por ATTR.^178^ Em uma coorte em Columbia, MP foi indicado em 43% dos pacientes com ATTRwt (*wild type*) e 36% dos pacientes com ATTRv.^192^ A indicação de MP nessa população deve seguir as recomendações tradicionais para implante.^193^

### 9.3. Opções Terapêuticas na Insuficiência Cardíaca Avançada

Na IC avançada associada à AC, o uso de estratégias avançadas de suporte incluindo dispositivos de assistência circulatória mecânica e transplante apresenta desafios e particularidades, especialmente por se tratar de doença multissistêmica. Além disso, pelo tamanho reduzido da cavidade do ventrículo esquerdo e o frequente acometimento do ventrículo direito, pode haver limitação ao uso de dispositivos de assistência circulatória mecânica de longa duração.^193^^,^^194^ Apesar de, historicamente, o transplante cardíaco apresentar uma pior curva de sobrevida na AC,^191^ os resultados mais recentes estão semelhantes às outras etiologias.^195^ Esta mudança está relacionada a uma melhor seleção dos pacientes e estratégias específicas, como o transplante duplo na AC por ATTRv e o transplante cardíaco precedendo o transplante de medula óssea na AL.^196^

A Tabela 12 resume as recomendações para o tratamento da AC.

## 10. Centros de Excelência/Referência e Novos Modelos de Remuneração do Tratamento

O acesso ao cuidado comprometido com melhoria contínua da qualidade assistencial é pilar fundamental para alcançar resultados de excelência, sendo item crítico nos casos das doenças complexas como a AC. Os gestores públicos e privados, pacientes, profissionais de saúde e todos envolvidos no cuidado ao paciente com amiloidose estão hoje comprometidos na construção de uma jornada que possibilite o cuidado certo e no lugar certo.

A AC deve ser abordada tendo como ponto fundamental o rápido diagnóstico, particularmente nas formas AL, e a disponibilidade de equipes multidisciplinares com comprovada experiência e utilizando protocolos clínicos embasados nas melhores evidências científicas. Ao lado disso, é necessário promover cuidado integral e monitorar e publicar os resultados assistenciais. Nesses centros de referência, deve-se observar um modelo de prática integrada e colaborativa, com estrutura de telemedicina para apoiar o diagnóstico de centros mais distantes, monitorar o paciente e promover pesquisa clínica, base para incorporação futura de novas tecnologias (terapias emergentes). Essa iniciativa está disponível em vários lugares do mundo e está sendo implementada com sucesso em algumas regiões do nosso país.^187^^–^^199^

Adicionalmente, é relevante considerar que os centros de referência devem ser os pilares para a construção de um Registro Nacional de Amiloidose Cardíaca, permitindo melhor conhecimento da epidemiologia no nosso meio e da qualidade do cuidado, além de mensurar os desfechos clínicos centrados no paciente, de forma a contribuir para elaboração de políticas públicas.

A construção de novos modelos de financiamento dos medicamentos de alto custo para doenças raras vem sendo discutida, testada em estudos de farmacoeconomia e implementada ao redor do mundo.^200^ Acoplar uma estratégia de compartilhamento de risco entre as empresas farmacêuticas, fontes pagadoras e provedores de saúde, com base na avaliação de desfechos clínicos e no impacto de protocolos clínicos, deve envolver pesquisa clínica. Esse novo paradigma começa a ser delineado e discutido em nosso país no contexto das doenças raras e pode ser empregado na AC.^201^^,^^202^ A sustentabilidade do sistema de saúde e a garantia de acesso ao tratamento de excelência aos pacientes com AC envolve, cada vez mais, o papel da Sociedade Brasileira de Cardiologia (SBC) e dos seus departamentos científicos/grupos de estudos. A SBC está promovendo debate sobre as boas práticas assistenciais em todas essas dimensões críticas para a construção de um sistema de saúde centrado nas necessidades do paciente e combatendo desperdícios.

De acordo com a avaliação da incorporação de novos tratamentos no Sistema Único de Saúde (SUS) para as doenças raras com o apoio da CONITEC (Comissão Nacional de Incorporação Tecnológica), foram incorporados cerca de 52% dos medicamentos avaliados. Nossa visão é que estamos construindo um novo cenário de tal forma, que o embasamento técnico, à luz de dados robustos e com o apoio de protocolos clínicos e centros de referência, possa substituir o modelo da busca de acesso via judicialização e seus impactos sobre os gastos da União.^197^

## 11. Lacunas do Conhecimento e Perspectivas Futuras

Apesar dos amplos avanços recentemente alcançados no entendimento da AC, ainda há inúmeras lacunas do conhecimento em torno desta doença complexa e plurifacetada que necessitam ser elucidadas. O diagnóstico da amiloidose, assim como seu tratamento, é uma área em franca evolução, o que pode ser ilustrado pelos 638 estudos recentemente completados ou em andamento registrados no *site* https://www.clinicaltrials.gov.

Na área da imagem cardiovascular para diagnóstico da AC, além da cintilografia cardíaca com radiotraçadores ósseos, como o ^99m^Tc-pirofosfato, que permite a obtenção de imagens moleculares do acúmulo de fibras amiloides no miocárdio, temos como perspectivas futuras o uso de imagens de PET usando ^18^F-Florbetapir em nervos periféricos e/ou outros locais extracardíacos como mais uma ferramenta diagnóstica, porém ainda não disponível no Brasil.^203^^,^^204^ Elastografia, ultrassonografia e RMC também estão sendo usadas para avaliação do grau de fibrose miocárdica e poderão ser ferramentas adicionais e promissoras para auxiliar no diagnóstico e prognóstico.

Trabalhos recentes têm se concentrado na análise do transcriptoma, em busca de diferenças de expressão entre indivíduos saudáveis e doentes, com análise molecular e genômica integrativa. O transcriptoma de pacientes com amiloidose AL é mais semelhante ao dos pacientes com gamopatia monoclonal de significado indeterminado. Além disso, o nível de microRNA circulante, que conhecido por se correlacionar com dano cardíaco, é aumentado nos pacientes AL. Por meio da análise de componentes principais, têm sido demonstrados perfis fenotípicos altamente sobrepostos entre AL e gamopatia monoclonal de significado indeterminado e mieloma múltiplo.^205^^,^^206^

Adicionalmente, a inteligência artificial (IA) aplicada na análise de dados disponíveis de banco de dados de prontuários médicos emerge como uma estratégia promissora para identificar indivíduos com sinais de alerta e permitir o diagnóstico mais precoce da AC, com potencial para reduzir o atraso para o início dos tratamentos modificadores da evolução da doença.

O desenvolvimento de novas terapias específicas para AC por ATTR é um campo intenso de investigação. Terapias de silenciamento gênico da TTR que se mostraram efetivas para o tratamento da polineuropatia amiloide hereditária estão sendo atualmente testadas em grandes estudos multicêntricos em pacientes com AC por ATTR, incluindo as plataformas de RNA de interferência, com o patisiran no estudo APOLLO-B (ClinicalTrials.gov Identifier: NCT03997383); e a tecnologia de oligonucleotídeos *anti-sense* no ensaio clínico CARDIO-TTRansform, testando um novo fármaco de segunda geração, AKCEA-TTR-LRx (ClinicalTrials.gov Identifier: NCT04136171).

Devido ao potencial arritmogênico e à lesão do sistema de condução associados ao depósito amiloide, é frequente o uso de dispositivos implantáveis como marca-passo ou cardiodesfibriladores implantáveis como estratégia de redução de mortalidade e aumento de sobrevida nessa população. Por outro lado, os indivíduos com AC e fibrilação ou *flutter* atrial exibem alto risco de eventos cardioembólicos e tem sido recomendado o tratamento com anticoagulantes. Essas intervenções, apesar de terem um racional fisiopatológico para aplicação em pacientes com amiloidose, ainda precisam ser testadas em ensaios clínicos adequados, sendo áreas importantes para investigação clínica futura. Além disso, o transplante cardíaco tem se mostrado como estratégia segura nesses pacientes, mas o uso de ventrículo artificial e a possibilidade de combinação das terapias devem ainda ser analisados em ensaios clínicos adequados.

É fundamental que esperemos os resultados de estudos distintos testando diferentes intervenções e terapias específicas, para que possamos definir as melhores opções e combinações de tratamento para essa doença, tendo em mente que: 1) não conhecemos completamente todos os detalhes da sua fisiopatologia; 2) não temos compreensão e experiência ampla de como as medicações funcionam nessa doença; 3) ainda não temos uma avaliação acurada, detalhada e de longo prazo dos riscos e benefícios dos diferentes tratamentos; 4) e também não conhecemos a relação entre dose e resposta das diferentes medicações para esse cenário clínico.

Dessa forma, julgamos que são ações relevantes e fundamentais a serem implantadas:

1. Criação de novos centros de referência/excelência em AC.
2. Capacitação de profissionais para o reconhecimento precoce e encaminhamento para centros especializados.
3. Estímulo para elaboração de um Registro Nacional de Amiloidose Cardíaca.
4. Organização e debate dos desafios para uma “Jornada do Paciente com Amiloidose” com segurança e qualidade.
5. Discussão de novos modelos de remuneração e de atenção na AC.
6. Incentivo à pesquisa clínica da AC em nosso país.
