# Supplementary material for: Posicionamento sobre Diagnóstico e Tratamento da Amiloidose Cardíaca – 2021
Source: Arq Bras Cardiol. 2021 Sep 1;117(3):561–98. [Article in Portuguese] doi: 10.36660/abc.20210718 (PMC8462947; doi:10.36660/abc.20210718)
Supplement: Translation

# Translation: Position Statement on Diagnosis and Treatment of Cardiac Amyloidosis – 2021

**Authors:** Marcus V. Simões, Fabio Fernandes, Fabiana G. Marcondes-Braga, Philip Scheinberg, Edileide de Barros Correia, Luis Eduardo P. Rohde, Fernando Bacal, Silvia Marinho Martins Alves, Sandrigo Mangini, Andréia Biolo, Luis Beck-da-Silva, Roberta Shcolnik Szor, Wilson Marques, Acary Souza Bulle Oliveira, Márcia Waddington Cruz, Bruno Vaz Kerges Bueno, Ludhmila Abrahão Hajjar, Aurora Felice Castro Issa, Felix José Alvarez Ramires, Otavio Rizzi Coelho, André Schmidt, Ibraim Masciarelli Francisco Pinto, Carlos Eduardo Rochitte, Marcelo Luiz Campos Vieira, Cláudio Tinoco Mesquita, Celso Dario Ramos, José Soares-Junior, Minna Moreira Dias Romano, Wilson Mathias, Marcelo Iório Garcia, Marcelo Westerlund Montera, Marcelo Dantas Tavares de Melo, Sandra Marques e Silva, Pedro Manoel Marques Garibaldi, Aristóteles Comte de Alencar, Renato Delascio Lopes, Diane Xavier de Ávila, Denizar Viana, José Francisco Kerr Saraiva, Manoel Fernandes Canesin, Glaucia Maria Moraes de Oliveira, Evandro Tinoco Mesquita

**Affiliations:** 1 Universidade de São Paulo Ribeirão Preto Brazil Faculdade de Medicina de Ribeirão Preto da Universidade de São Paulo, Ribeirão Preto – Brazil; 2 Universidade de São Paulo São Paulo SP Brazil Instituto do Coração (InCor) do Hospital das Clínicas da Faculdade de Medicina da Universidade de São Paulo (HCFMUSP), São Paulo, SP – Brazil; 3 Hospital da Beneficência Portuguesa de São Paulo São Paulo SP Brazil Hospital da Beneficência Portuguesa de São Paulo, São Paulo, SP – Brazil; 4 Instituto Dante Pazzanese de Cardiologia São Paulo SP Brazil Instituto Dante Pazzanese de Cardiologia, São Paulo, SP – Brazil; 5 Hospital de Clínicas de Porto Alegre Porto Alegre RS Brazil Hospital de Clínicas de Porto Alegre, Porto Alegre, RS – Brazil; 6 Hospital Moinhos de Vento Porto Alegre RS Brazil Hospital Moinhos de Vento, Porto Alegre, RS – Brazil; 7 Universidade Federal do Rio Grande do Sul Porto Alegre RS Brazil Universidade Federal do Rio Grande do Sul (UFRGS), Porto Alegre, RS – Brazil; 8 Pronto Socorro Cardiológico de Pernambuco Recife PE Brazil Pronto Socorro Cardiológico de Pernambuco (PROCAPE), Recife, PE – Brazil; 9 Universidade de Pernambuco Recife PE Brazil Universidade de Pernambuco (UPE), Recife, PE – Brazil; 10 Fundação Faculdade de Medicina São Paulo SP Brazil Fundação Faculdade de Medicina, São Paulo, SP – Brazil; 11 Universidade de São Paulo São Paulo SP Brazil Instituto do Câncer do Estado de São Paulo da Faculdade de Medicina da Universidade de São Paulo, São Paulo, SP – Brazil; 12 Universidade Federal de São Paulo São Paulo SP Brazil Universidade Federal de São Paulo, São Paulo, SP – Brazil; 13 Universidade Federal do Rio de Janeiro Rio de Janeiro RJ Brazil Hospital Universitário Clementino Fraga Filho (HUCFF) da Universidade Federal do Rio de Janeiro (UFRJ), Rio de Janeiro, RJ – Brazil; 14 Faculdade de Ciências Médicas da Santa Casa de São Paulo São Paulo SP Brazil Faculdade de Ciências Médicas da Santa Casa de São Paulo, São Paulo, SP – Brazil; 15 Instituto Nacional de Cardiologia Rio de Janeiro RJ Brazil Instituto Nacional de Cardiologia, Rio de Janeiro, RJ – Brazil; 16 Hospital Israelita Albert Einstein São Paulo SP Brazil Hospital Israelita Albert Einstein, São Paulo, SP – Brazil; 17 Universidade Estadual de Campinas Campinas SP Brazil Faculdade de Ciências Médicas da Universidade Estadual de Campinas (UNICAMP), Campinas, SP – Brazil; 18 Grupo Fleury São Paulo SP Brazil Grupo Fleury, São Paulo, SP – Brazil; 19 Hospital do Coração São Paulo SP Brazil Hospital do Coração (HCor), São Paulo, SP – Brazil; 20 Hospital Pró-Cardíaco Rio de Janeiro RJ Brazil Hospital Pró-Cardíaco, Rio de Janeiro, RJ – Brazil; 21 Universidade Federal Fluminense Rio de Janeiro RJ Brazil Universidade Federal Fluminense (UFF), Rio de Janeiro, RJ – Brazil; 22 Universidade Federal da Paraíba João Pessoa PB Brazil Universidade Federal da Paraíba (UFPB), João Pessoa, PB – Brazil; 23 Hospital de Base do DF Brasília DF Brazil Hospital de Base do DF, Brasília, DF – Brazil; 24 Duke University Durham USA Duke University, Durham – USA; 25 Complexo Hospitalar de Niterói Rio de Janeiro RJ Brazil Complexo Hospitalar de Niterói, Rio de Janeiro, RJ – Brazil; 26 Hospital e Maternidade Christóvão da Gama Santo André SP Brazil Hospital e Maternidade Christóvão da Gama, Santo André, SP – Brazil; 27 Hospital Universitário Antônio Pedro Rio de Janeiro RJ Brazil Hospital Universitário Antônio Pedro (Huap), Rio de Janeiro, RJ – Brazil; 28 Universidade do Estado do Rio de Janeiro Rio de Janeiro RJ Brazil Universidade do Estado do Rio de Janeiro, Rio de Janeiro, RJ – Brazil; 29 Sociedade Campineira de Educação e Instrução Campinas SP Brazil Sociedade Campineira de Educação e Instrução, Campinas, SP – Brazil; 30 Universidade Estadual de Londrina Londrina PR Brazil Hospital Universitário da Universidade Estadual de Londrina, Londrina, PR – Brazil; 31 Universidade Federal do Rio de Janeiro Rio de Janeiro RJ Brazil Universidade Federal do Rio de Janeiro (UFRJ), Rio de Janeiro, RJ – Brazil; 32 Centro de Ensino e Treinamento Edson de Godoy Bueno Rio de Janeiro RJ Brazil Centro de Ensino e Treinamento Edson de Godoy Bueno/UHG, Rio de Janeiro, RJ – Brazil

**Table:** 

Position Statement on Diagnosis and Treatment of Cardiac Amyloidosis – 2021	
The report below lists declarations of interest as reported to the SBC by the experts during the period of the development of these statement, 2020/2021.	
Expert	Type of relationship with industry
Acary Souza Bulle Oliveira	Nothing to be declared
André Schmidt	Financial declaration
	A - Economically relevant payments of any kind made to (i) you, (ii) your spouse/partner or any other person living with you, (iii) any legal person in which any of these is either a direct or indirect controlling owner, business partner, shareholder or participant; any payments received for lectures, lessons, training instruction, compensation, fees paid for participation in advisory boards, investigative boards or other committees, etc. From the brazilian or international pharmaceutical, orthosis, prosthesis, equipment and implants industry:
	- Bayer: Anticoagulants.
	Other relationships
	Funding of continuing medical education activities, including travel, accommodation and registration in conferences and courses, from the brazilian or international pharmaceutical, orthosis, prosthesis, equipment and implants industry:
	- Sanofi: Anticoagulants.
Andréia Biolo	Nothing to be declared
Aristóteles Comte de Alencar Neto	Nothing to be declared
Aurora Felice Castro Issa	Nothing to be declared
Bruno Vaz Kerges Bueno	Financial declaration
	A - Economically relevant payments of any kind made to (i) you, (ii) your spouse/partner or any other person living with you, (iii) any legal person in which any of these is either a direct or indirect controlling owner, business partner, shareholder or participant; any payments received for lectures, lessons, training instruction, compensation, fees paid for participation in advisory boards, investigative boards or other committees, etc. From the brazilian or international pharmaceutical, orthosis, prosthesis, equipment and implants industry:- Pfiser: Eliquis; Alnylam: Patisiran.
Carlos Eduardo Rochitte	Nothing to be declared
Celso Dario Ramos	Nothing to be declared
Cláudio Tinoco Mesquita	Financial declaration
	A - Economically relevant payments of any kind made to (i) you, (ii) your spouse/partner or any other person living with you, (iii) any legal person in which any of these is either a direct or indirect controlling owner, business partner, shareholder or participant; any payments received for lectures, lessons, training instruction, compensation, fees paid for participation in advisory boards, investigative boards or other committees, etc. From the brazilian or international pharmaceutical, orthosis, prosthesis, equipment and implants industry:
	- Pfizer: Participation in amyloidosis advisory board.
	B - Research funding under your direct/personal responsibility (directed to the department or institution) from the brazilian or international pharmaceutical, orthosis, prosthesis, equipment and implants industry:
	- Alnylam: Principal Investigator of the Apollo B study with Patisiran.
	Other relationships
	Funding of continuing medical education activities, including travel, accommodation and registration in conferences and courses, from the brazilian or international pharmaceutical, orthosis, prosthesis, equipment and implants industry:
	- Pfizer: Amyloidosis.
Denizar Viana	Nothing to be declared
Diane Xavier de Ávila	Financial declaration
	A - Economically relevant payments of any kind made to (i) you, (ii) your spouse/partner or any other person living with you, (iii) any legal person in which any of these is either a direct or indirect controlling owner, business partner, shareholder or participant; any payments received for lectures, lessons, training instruction, compensation, fees paid for participation in advisory boards, investigative boards or other committees, etc. From the brazilian or international pharmaceutical, orthosis, prosthesis, equipment and implants industry:
	- Alnylam: Patisiran.
	Other relationships
	Funding of continuing medical education activities, including travel, accommodation and registration in conferences and courses, from the brazilian or international pharmaceutical, orthosis, prosthesis, equipment and implants industry:
	- Alnylam: Patisiran.
Edileide de Barros Correia	Financial declaration
	A - Economically relevant payments of any kind made to (i) you, (ii) your spouse/partner or any other person living with you, (iii) any legal person in which any of these is either a direct or indirect controlling owner, business partner, shareholder or participant; any payments received for lectures, lessons, training instruction, compensation, fees paid for participation in advisory boards, investigative boards or other committees, etc. From the brazilian or international pharmaceutical, orthosis, prosthesis, equipment and implants industry:
	- Pfizer: Tafamidis (Vyndaqel); Alnylam: Patsiram (Onpatro); Takeda: Replagal.
Evandro Tinoco Mesquita	Employment relationship with the brazilian or international pharmaceutical, orthosis, prosthesis, equipment and implants industry, as well as any employment relationship with health insurance companies or medical audit companies (including part-time jobs) in the year to which your declaration refers:
	- UnitedHealth Group.
Fabiana G. Marcondes-Braga	Financial declaration
	A - Economically relevant payments of any kind made to (i) you, (ii) your spouse/partner or any other person living with you, (iii) any legal person in which any of these is either a direct or indirect controlling owner, business partner, shareholder or participant; any payments received for lectures, lessons, training instruction, compensation, fees paid for participation in advisory boards, investigative boards or other committees, etc. From the brazilian or international pharmaceutical, orthosis, prosthesis, equipment and implants industry:
	- Novartis: Lectures; AstraZeneca: Lectures and Advisory Council; Boehringer: Advisory Council.
Fabio Fernandes	Declaração financeira
	A - Pagamento de qualquer espécie e desde que economicamente apreciáveis, feitos a (i) você, (ii) ao seu cônjuge/ companheiro ou a qualquer outro membro que resida com você, (iii) a qualquer pessoa jurídica em que qualquer destes seja controlador, sócio, acionista ou participante, de forma direta ou indireta, recebimento por palestras, aulas, atuação como proctor de treinamentos, remunerações, honorários pagos por participações em conselhos consultivos, de investigadores, ou outros comitês, etc. Provenientes da indústria farmacêutica, de órteses, próteses, equipamentos e implantes, brasileiras ou estrangeiras:
	- Pfiser, Alnylan: Lectures.
	Other relationships
	Funding of continuing medical education activities, including travel, accommodation and registration in conferences and courses, from the brazilian or international pharmaceutical, orthosis, prosthesis, equipment and implants industry:
	- Phifesr: Participation in international congress.
Felix José Alvarez Ramires	Financial declaration
	A - Economically relevant payments of any kind made to (i) you, (ii) your spouse/partner or any other person living with you, (iii) any legal person in which any of these is either a direct or indirect controlling owner, business partner, shareholder or participant; any payments received for lectures, lessons, training instruction, compensation, fees paid for participation in advisory boards, investigative boards or other committees, etc. From the brazilian or international pharmaceutical, orthosis, prosthesis, equipment and implants industry:
	- Novartis: Sacubitril/Valsartana; Pfizer: Patisiran; Merck: Vericiquat; Amgen.
Fernando Bacal	Nothing to be declared
Glaucia Maria Moraes de Oliveira	Nothing to be declared
Ibraim Masciarelli Francisco Pinto	Financial declaration
	A - Economically relevant payments of any kind made to (i) you, (ii) your spouse/partner or any other person living with you, (iii) any legal person in which any of these is either a direct or indirect controlling owner, business partner, shareholder or participant; any payments received for lectures, lessons, training instruction, compensation, fees paid for participation in advisory boards, investigative boards or other committees, etc. From the brazilian or international pharmaceutical, orthosis, prosthesis, equipment and implants industry:
	- Novo Nordisk: Diabetes.
José Francisco Kerr Saraiva	Nothing to be declared
José Soares-Junior	Nothing to be declared
Ludhmila Abrahão Hajjar	Nothing to be declared
Luis Beck-da-Silva	Financial declaration
	A - Economically relevant payments of any kind made to (i) you, (ii) your spouse/partner or any other person living with you, (iii) any legal person in which any of these is either a direct or indirect controlling owner, business partner, shareholder or participant; any payments received for lectures, lessons, training instruction, compensation, fees paid for participation in advisory boards, investigative boards or other committees, etc. From the brazilian or international pharmaceutical, orthosis, prosthesis, equipment and implants industry:
	- Novartis: Heart failure; AstraZeneca: Heart failure.
	B - Research funding under your direct/personal responsibility (directed to the department or institution) from the brazilian or international pharmaceutical, orthosis, prosthesis, equipment and implants industry:
	- Amgen: Heart failure.
Luis Eduardo P. Rohde	Financial declaration
	A - Economically relevant payments of any kind made to (i) you, (ii) your spouse/partner or any other person living with you, (iii) any legal person in which any of these is either a direct or indirect controlling owner, business partner, shareholder or participant; any payments received for lectures, lessons, training instruction, compensation, fees paid for participation in advisory boards, investigative boards or other committees, etc. From the brazilian or international pharmaceutical, orthosis, prosthesis, equipment and implants industry:
	- Pfizer: Tafamidis; Novartir: Sacubitril-Valsartan; AstraZeneca: Dpaglifozina; Boehringer Ingelheim: Empaglifozina; Merck: Bisoprolol; Amgen: Omecamtiv-Mercabil.
	Other relationships
	Funding of continuing medical education activities, including travel, accommodation and registration in conferences and courses, from the brazilian or international pharmaceutical, orthosis, prosthesis, equipment and implants industry:
	- AstraZeneca: Dapaglifozina; Boehringer Ingelheim: Empaglifozina.
Manoel Fernandes Canesin	Nothing to be declared
Marcelo Dantas Tavares de Melo	Nothing to be declared
Marcelo Iório Garcia	Nothing to be declared
Marcelo Luiz Campos Vieira	Nothing to be declared
Marcelo Westerlund Montera	Nothing to be declared
Márcia Waddington Cruz	Financial declaration
	A - Economically relevant payments of any kind made to (i) you, (ii) your spouse/partner or any other person living with you, (iii) any legal person in which any of these is either a direct or indirect controlling owner, business partner, shareholder or participant; any payments received for lectures, lessons, training instruction, compensation, fees paid for participation in advisory boards, investigative boards or other committees, etc. From the brazilian or international pharmaceutical, orthosis, prosthesis, equipment and implants industry:
	- Pfizer, Alnylam, Genzyme, Ionis, Prothena, FoldRx, PTC, NIH: Presentation at a scientific event as principal investigator of clinical trials and as a consultant.
	B - Research funding under your direct/personal responsibility (directed to the department or institution) from the brazilian or international pharmaceutical, orthosis, prosthesis, equipment and implants industry:
	- Pfizer, Alnylam, Genzyme, Ionis, Prothena, FoldRx, PTC, NIH: Presentation at a scientific event as principal investigator of clinical trials and as a consultant.
	Other relationships
	Funding of continuing medical education activities, including travel, accommodation and registration in conferences and courses, from the brazilian or international pharmaceutical, orthosis, prosthesis, equipment and implants industry:
	- Pfizer, Alnylam, Genzyme, Ionis, Prothena, FoldRx, PTC, NIH: Presentation at a scientific event as principal investigator of clinical trials and as a consultant.
Marcus V. Simões	Financial declaration
	A - Economically relevant payments of any kind made to (i) you, (ii) your spouse/partner or any other person living with you, (iii) any legal person in which any of these is either a direct or indirect controlling owner, business partner, shareholder or participant; any payments received for lectures, lessons, training instruction, compensation, fees paid for participation in advisory boards, investigative boards or other committees, etc. From the brazilian or international pharmaceutical, orthosis, prosthesis, equipment and implants industry:
	- Novartis: Entresto; AstraZeneca: Dapagliflozina.
	B - Research Funding Under Your Direct/Personal Responsibility (Directed To The Department Or Institution) From The Brazilian Or International Pharmaceutical, Orthosis, Prosthesis, Equipment And Implants Industry:
	- Amgen: Omecamtiv/Mecarbil; Beringher Ingelheim: Empagliflozina.
Minna Moreira Dias Romano	Nothing to be declared
Otavio Rizzi Coelho Filho	Financial declaration
	A - Economically relevant payments of any kind made to (i) you, (ii) your spouse/partner or any other person living with you, (iii) any legal person in which any of these is either a direct or indirect controlling owner, business partner, shareholder or participant; any payments received for lectures, lessons, training instruction, compensation, fees paid for participation in advisory boards, investigative boards or other committees, etc. From the brazilian or international pharmaceutical, orthosis, prosthesis, equipment and implants industry:
	- Pfizer: Treatment for TTR amyloidosis; Takeda: Fabry Disease; Novartis: Heart failure; AstraZeneca; Daiichi Sankyo; Merck; Shire; Bayer.
	B - Research funding under your direct/personal responsibility (directed to the department or institution) from the brazilian or international pharmaceutical, orthosis, prosthesis, equipment and implants industry:
	- Pfizer: Treatment for TTR amyloidosis.
	Other relationships
	Funding of continuing medical education activities, including travel, accommodation and registration in conferences and courses, from the brazilian or international pharmaceutical, orthosis, prosthesis, equipment and implants industry:
	- AstraZeneca: Heart failure.
Pedro Manoel Marques Garibaldi	Nothing to be declared
Philip Scheinberg	Financial declaration
	A - Economically relevant payments of any kind made to (i) you, (ii) your spouse/partner or any other person living with you, (iii) any legal person in which any of these is either a direct or indirect controlling owner, business partner, shareholder or participant; any payments received for lectures, lessons, training instruction, compensation, fees paid for participation in advisory boards, investigative boards or other committees, etc. From the brazilian or international pharmaceutical, orthosis, prosthesis, equipment and implants industry:
	- Novartis, Abbvie, Roche: Hematology.
Renato Delascio Lopes	Financial declaration
	A - Economically relevant payments of any kind made to (i) you, (ii) your spouse/partner or any other person living with you, (iii) any legal person in which any of these is either a direct or indirect controlling owner, business partner, shareholder or participant; any payments received for lectures, lessons, training instruction, compensation, fees paid for participation in advisory boards, investigative boards or other committees, etc. From the brazilian or international pharmaceutical, orthosis, prosthesis, equipment and implants industry:
	- Bayer: Anticoagulante; Boehringer Ingleheim: Anticoagulação e Diabetes; Pfizer: Anticoagulação; Bristol-Myers Squibb; Daiichi Sankyo; Glaxo Smith Kline; Medtronic; Merck; Portola; Sanofi.
	B - Research funding under your direct/personal responsibility (directed to the department or institution) from the brazilian or international pharmaceutical, orthosis, prosthesis, equipment and implants industry:
	- Pfizer: Apixaban; Bayer: Rivaroxaban; Novartis: Sacubitril, Valsartan.
	Other relationships
	Funding of continuing medical education activities, including travel, accommodation and registration in conferences and courses, from the brazilian or international pharmaceutical, orthosis, prosthesis, equipment and implants industry:
	- Bayer: Rivaroxabana; Pfizer: Apixabana.
Roberta Shcolnik Szor	Financial declaration
	A - Economically relevant payments of any kind made to (i) you, (ii) your spouse/partner or any other person living with you, (iii) any legal person in which any of these is either a direct or indirect controlling owner, business partner, shareholder or participant; any payments received for lectures, lessons, training instruction, compensation, fees paid for participation in advisory boards, investigative boards or other committees, etc. From the brazilian or international pharmaceutical, orthosis, prosthesis, equipment and implants industry:
	- Pfizer: Amyloidosis.
	B - Financiamento de pesquisas sob sua responsabilidade direta/pessoal (direcionado ao departamento ou instituição) provenientes da indústria farmacêutica, de órteses, próteses, equipamentos e implantes, brasileiras ou estrangeiras:
	- Janssen-Cilag Farmacêutica Ltda.: Light chain amyloidosis.
	Other relationships
	Funding of continuing medical education activities, including travel, accommodation and registration in conferences and courses, from the brazilian or international pharmaceutical, orthosis, prosthesis, equipment and implants industry:
	- Janssen-Cilag Farmacêutica Ltda.: Light chain amyloidosis.
Sandra Marques e Silva	Financial declaration
	A - Economically relevant payments of any kind made to (i) you, (ii) your spouse/partner or any other person living with you, (iii) any legal person in which any of these is either a direct or indirect controlling owner, business partner, shareholder or participant; any payments received for lectures, lessons, training instruction, compensation, fees paid for participation in advisory boards, investigative boards or other committees, etc. From the brazilian or international pharmaceutical, orthosis, prosthesis, equipment and implants industry:
	- Sanofi, Takeda, Amicus Therapeutics: Rare deseases; Pfiser.
	Other relationships
	Funding of continuing medical education activities, including travel, accommodation and registration in conferences and courses, from the brazilian or international pharmaceutical, orthosis, prosthesis, equipment and implants industry:
	- Sanofi, Takeda, Amicus Therapeutics: Rare deseases; Pfiser.
Sandrigo Mangini	Financial declaration
	A - Economically relevant payments of any kind made to (i) you, (ii) your spouse/partner or any other person living with you, (iii) any legal person in which any of these is either a direct or indirect controlling owner, business partner, shareholder or participant; any payments received for lectures, lessons, training instruction, compensation, fees paid for participation in advisory boards, investigative boards or other committees, etc. From the brazilian or international pharmaceutical, orthosis, prosthesis, equipment and implants industry:
	- Novartis: Sacubitril/Valsartan; Pfizer: Rare diseases.
	Other relationships
	Funding of continuing medical education activities, including travel, accommodation and registration in conferences and courses, from the brazilian or international pharmaceutical, orthosis, prosthesis, equipment and implants industry:
	- Pfizer: Rare diseases
Silvia Marinho Martins Alves	Nothing to be declared
Wilson Marques Junior	Financial declaration
	A - Economically relevant payments of any kind made to (i) you, (ii) your spouse/partner or any other person living with you, (iii) any legal person in which any of these is either a direct or indirect controlling owner, business partner, shareholder or participant; any payments received for lectures, lessons, training instruction, compensation, fees paid for participation in advisory boards, investigative boards or other committees, etc. From the brazilian or international pharmaceutical, orthosis, prosthesis, equipment and implants industry:
	- Pfizer: Tafamidis; Alnylam: Patisiran; PTC: Inotersen.
	B - Research funding under your direct/personal responsibility (directed to the department or institution) from the brazilian or international pharmaceutical, orthosis, prosthesis, equipment and implants industry:
	- Pfizer: Amyloidosis.
	Other relationships
	Funding of continuing medical education activities, including travel, accommodation and registration in conferences and courses, from the brazilian or international pharmaceutical, orthosis, prosthesis, equipment and implants industry:
	- Pfizer: Tafamidis; Alnylam: Patisiran; PTC: Inotersen.
Wilson Mathias Junior	Nothing to be declared

## 1. Introduction

Significant advances in understanding cardiac amyloidosis (CA) have been made in recent years, leading to a thorough reformulation of its clinical significance. In addition to convincing evidence that CA is a relatively common cause of heart failure with preserved ejection fraction (HFpEF), we are witnessing the emergence of specific therapies that can change the course of the disease and prolong the survival of affected patients.

In parallel, relevant advances in cardiovascular imaging techniques have greatly contributed to earlier and more accurate identification of the disease. Cardiac scintigraphy with bone-seeking radiotracers has allowed non-invasive diagnosis of transthyretin (TTR) cardiac amyloidosis (ATTR-CA), eliminating the need for endomyocardial biopsy, which has greatly simplified the diagnostic flow.

Thus, we aim to present the most current recommendations for the diagnosis, prognostic staging, and treatment of CA based on a critical review of the current scientific evidence.

In this position paper, the recommendations and levels of evidence are classified according to the following parameters:

**Table:** 

Grades of recommendation:	
**Grade I –**	Conclusive evidence or, in its absence, general consensus that the procedure is safe and useful/effective
**Grade IIa –**	Conflicting evidence and/or divergence of opinion about the procedure’s safety and usefulness/effectiveness. The weight of evidence/opinion favors it and most studies/experts approve it
**Grade IIb –**	Conflicting evidence and/or divergence of opinion about the procedure’s safety and usefulness/effectiveness. Its safety and usefulness/efficacy are less well established, and opinion is not predominantly in its favor
**Grade III –**	Evidence and/or consensus that the procedure is not useful/effective and, in some cases, may be harmful
**Levels of evidence:**	
**Level A –**	Data obtained from several large randomized studies or concordant and/or robust meta-analysis of randomized clinical trials
**Level B –**	Data obtained from less robust meta-analysis, from a single randomized study, or from non-randomized (observational) studies
**Level C –**	Data obtained from a consensus of expert opinion

## 2. General Concepts

Systemic amyloidosis is caused by tissue deposition of fibrillar and insoluble protein aggregates in different organs, including the heart, which leads to organ dysfunction.^1^ More than 30 types of amyloidogenic proteins have been described,^2^ and five of them can affect the heart (immunoglobulin heavy and light chain (AL), TTR, amyloid A, and apolipoprotein A1), with the AL and ATTR types accounting for 95% of all CA cases, both in its wild type (ATTRwt) and hereditary/variant (ATTRv) forms.^3^^–^^7^

TTR is a protein composed of four monomers, which circulate as a tetramer.^8^ It acts as a thyroxine and retinol (vitamin A) transporter under physiological conditions. The limiting step in the amyloid fibril formation rate is the tetramer’s dissociation into monomers, which may involve proteolysis. Subsequently, partial denaturation of the monomer allows for incorrect assembly in various aggregate structures. Amyloidosis through mutation of the TTR gene (ATTRv) has an autosomal dominant character. This gene is located on chromosome 18, and more than 140 mutations of it have been described. By producing less stable TTR, aggressive and systemic amyloid deposition occurs.^9^

In ATTRwt, the amino acid sequence is normal and the process by which the wild-type protein becomes unstable and aggregates into amyloid fibrils is not completely clear. However, aging appears to be involved in its pathophysiology.^8^^,^^9^

In the AL form, amyloidogenic light chains originate from plasma cells or, less frequently, abnormal B lymphocytes. Thus, it is a clonal and neoplastic hematologic disease. In the heart, the deposition of amyloid fibrils causes structural damage by increasing cardiac and vascular rigidity, impairing cardiac contraction and relaxation and creating conduction disturbances. In parallel, circulating light chains are directly toxic to the myocardium through lysosomal dysfunction, defective autophagy, production of reactive oxygen species, cell and mitochondrial dysfunction, alterations in cardiomyocyte calcium homeostasis and, finally, cell death.^10^

Figure 1 represents the physiopathogenesis of transthyretin (TTR) and light chain (AL) cardiac amyloidosis.

**Figure 1:** Physiopathogenesis of cardiac amyloidosis.

Different subtypes of amyloidosis can lead to overlapping clinical manifestations and, once diagnosed, it is essential to correctly characterize the precursor protein to determine a specific treatment.^11^^–^^13^

Depending on the affected organs and degree of dysfunction, a wide spectrum of clinical manifestations can be observed, with a progressive and potentially fatal evolution. The main organs affected by systemic amyloidosis are the heart, kidneys, eyes, central and peripheral nervous system, and liver. Nonspecific clinical manifestations are frequently observed and include fatigue, weight loss, peripheral edema and orthostatic hypotension. For this reason, late diagnosis is common. Thus, knowledge of the disease and a high degree of clinical suspicion are necessary to complete the diagnosis.

In ATTRv, depending on the mutation, the clinical picture is dominated by neuropathy or heart disease. In ATTRwt, heart disease is the main clinical manifestation, occurring mainly in elderly men who develop HFpEF without previously known risk factors.

Some extracardiac alterations may precede CA by several years, especially bilateral carpal tunnel syndrome and spontaneous rupture of the biceps tendon. It is essential to recognize such signs as part of the clinical picture of amyloidosis, which could lead to earlier diagnosis and specific treatments that could prevent the progression of heart disease.^14^^,^^15^

There is a higher incidence of ATTRwt in older patients, usually those over 70 years of age. However, since the clinical manifestations of ATTRv also usually occur in older adults, age should not be taken into account when differentiating between the two forms of ATTR. Regarding sex, there is a strong predominance (80% to 90%) of ATTRwt in men.

Regarding ATTRv, V30M is the most widespread mutation worldwide, being endemic in Portugal, Sweden, and Japan. It is probably the most common form in Brazil. Another common mutation is V122I, which is present in 3.4% of African-Americans and is related to the development of heart disease in patients over 60 years of age.^7^^,^^9^^,^^16^^,^^17^

Table 1 summarizes the demographic and clinical characteristics of the AL, ATTRv, and ATTRwt subtypes.

**Table 1:** Demographic and clinical presentation aspects, comparative between the forms AL, ATTRv and ATTRwt

Demographic Aspects	AL	ATTRv	ATTRwt
Starting age (years)	> 60 years	Depends on the genotype	> 70 years
Sex	Slightly predominant in men	No predominance	Predominant in men
Ethnic origin	None	Most frequent mutation: African-Americans = Val122Ile Portuguese = Val30Met	None
Prevalence/Incidence	10 cases/million people/year, increases with age	Variable according to genotype	Unknown, increases with age
Clinical aspects			
HFpEF	√	√	√
Peripheral and/or autonomic neuropathy	√	√	-
Proteinuria	√	-	-
Periorbital hyperpigmentation	√	-	-
Macroglossia	√	-	-
Bilateral carpal tunnel syndrome	√	-	√
Spontaneous biceps tendon rupture	-	-	√

*HFpEF: heart failure with preserved ejection fraction*.

The incidence of AL-CA is 6-10/million people/year and it is considered the main cause of CA.^18^ With the development of less invasive CA diagnosis techniques for ATTR^19^and the prospect of more effective treatments, the number of diagnosed cases, especially of ATTRwt, has been increasing significantly.^20^ AL is currently the most common cause of CA. Studies have found ATTR deposits in the heart of 13% of HFpEF patients^21^ and in 25% of autopsied older adults,^22^^,^^23^ mainly males.^24^

Thus, CA could be considered an underdiagnosed condition, rather than a rare disease. Recent data from the USA indicate a progressive increase in CA prevalence (from 18 to 55.2/100,000 person-years),^25^ which supports this idea. The patient’s journey to diagnosis is long; it is estimated that there is a delay of more than 2 years from symptom onset to diagnosis, with the involvement of an average of five different professionals.^26^ Thus, it is essential to disseminate knowledge about CA; clinicians and cardiologists must give greater consideration to this entity, aiming at earlier diagnosis and adequate therapeutic guidance, thus improving patient prognosis and survival.

Regarding prognosis, AL affects multiple organs and is more aggressive than other subtypes. Late diagnosis is associated with high early mortality in the first 6 to 12 months due to advanced heart disease complications.^7^^,^^8^The median estimated survival for ATTRwt is 3.6 years, while the prognosis for ATTRv depends on the mutation. In cases of neurological phenotype, the progression of neuropathy leads to sensorimotor disability, although mortality is more associated with cardiac impairment.^12^^,^^13^

## 3. Neurological Manifestations

Mutations in the TTR gene are associated with a wide variety of clinical manifestations, which reflect the deposition of the variant protein in different types of tissues. Cardiac and peripheral nervous system tissue are the most frequent; the former is particularly associated with the V122I mutation, and the latter with the V30M mutation.^27^

In this chapter, we will describe the main neurological manifestations suggestive of ATTRv.

Neurological manifestations in ATTRv can be divided into peripheral neuropathy, ie, late manifestations of central nervous system involvement linked to amyloid angiopathy, and central nervous system manifestations associated with oculoleptomeningeal infiltration.

### 3.1. Peripheral Neuropathy

The involvement of the peripheral nerves in ATTRv is typically mixed sensory and motor length-dependent axonal neuropathy, ie, it initially affects more distal segments of the limbs, especially the lower ones, progressing to the proximal segments and upper limbs.^28^^,^^29^

In its early onset form (< 50 years of age), it is usually associated with the ATTRv V30M (V50M) mutation, and thin fibers with little or no myelination (autonomic, heat, cold, and pain) are initially affected. This is followed, in the degree that the disease progresses, by thick fibers, which are very myelinated and responsible for vibratory, postural-kinetic, and motor sensitivity. The initial symptoms are erectile dysfunction, early satiety, nausea, vomiting, diarrhea, constipation, alternating diarrhea with constipation, orthostatic hypotension, syncope, arrhythmias, altered atrioventricular conduction, dry eye, urinary retention or incontinence, neuropathic pain, lost sensitivity to heat and cold, and significant weight loss. The initial phase can include painless lesions, plantar perforating ulcers and their repercussions, such as localized infections, cellulitis, osteomyelitis, and even septicemia. After a few years, gait instability and muscle atrophy appear, always evolving from distal to proximal segments.^28^^,^^29^

In its late forms, neuropathy compromises all types of fibers, although dysautonomia is not as important, at least in the initial phase. These forms can be associated with V30M or a number of other mutations, and the evolution is usually more aggressive. In a Brazilian study, 26% of ATTRv patients with V30M had late onset.^30^

Bilateral carpal tunnel syndrome is a frequent manifestation in ATTRv and may be the initial manifestation. Although it can be associated with any mutation, it is particularly important in some of them, including TTR V122I. This mutation appears to be frequent in Brazil and is associated with heart disease, which it can precede by several years.^31^

### 3.2. Central Nervous System Manifestations

Prolonging survival, which was initially associated with liver transplantation and is currently possible with new drugs, has enabled the appearance of previously uncommon manifestations. The long-term production of TTR by the choroid plexus (only 2% of the total) is associated with both amyloid angiopathy and meningeal infiltration. Amyloid angiopathy manifests as focal stroke-like, transient ischemic attack-like, or aura-like episodes, as well as an irritative epileptic type. In more severe cases, ischemia or even intracranial hemorrhaging can occur. The following neurological manifestations stand out: hearing impairment, migraine, dementia, cerebellar syndrome, myelopathy, and radiculopathy.

Some rare mutations have a predilection for oculocerebral involvement and lead to oculoleptomeningeal amyloidosis (ATTR Y69H: oculocerebral; Val30Gly: oculoleptomeningeal).^32^

**Stages of neuropathy**: The Coutinho stages for ATTRv are classified according to polyneuropathy. In stage 1 sensorimotor polyneuropathy affects gait, but walking support is not necessary. In stage 2, one or more support devices are needed for walking. In stage 3, the patient is confined to bed or a wheelchair.

### 3.3. Genetic Analysis

ATTRv is a disease of autosomal dominant inheritance but variable penetrance. It is mutation- and age-dependent, as well as regionally influenced.^33^ The V30Met mutation, for example, has an 80% and 91% penetrance in Portugal in individuals aged 50 and 70 years, respectively. On the other hand, in Sweden, the values are 11% and 36%, respectively, for the same age groups.

At least 140 different mutations have been described to date, but not all are pathogenic. Some polymorphisms are well-defined, while the significance of others is still indeterminate.^34^ This suggests that the diagnostic test must always include complete TTR gene sequencing and that care must be taken regarding the interpretation of rare or undescribed variants. Among pathogenic mutations, some predominantly cause neuropathy (V30Met), heart disease (V122I), or both (Leu58Hist) (Figure 2).^35^ It should be considered, however, that the genotypic/phenotypic correlation is not strict.

**Figure 2:** Distribution of hereditary transthyretin amyloidosis mutations associated with neurological, cardiological, and mixed phenotypes.

A special topic among genetic aspects is pre-symptomatic testing, ie, testing the relatives of individuals known to be affected. Unlike diagnostic testing, it must be performed by specialized personnel and there must be a support team, including a psychologist. It must include a preparation phase, pre-diagnosis, genetic testing and a post-result support phase. Such testing should not be performed on children and should only be applied to individuals who expressly state that it is their wish and are considered psychologically prepared for the results.^36^

## 4. Cardiovascular Manifestations

CA progresses as the cardiac extracellular matrix is infiltrated by amyloid fibrils, resulting in a progressive increase in the thickness of the ventricular wall and a marked increase in chamber stiffness, resulting in impaired diastolic function, which leads to heart failure with restrictive physiology.^37^ Systolic function is also compromised and is usually indicated by abnormal longitudinal tension despite normal ejection fraction, which may be preserved until the late stages of the disease.^38^^–^^40^ Atrial amyloid infiltration is frequent, leading to contractile dysfunction. Deposits can also occur in the heart valves, usually without causing major dysfunctions, as well as in the perivascular region.^3^

Thus, the most frequent clinical manifestation is heart failure syndrome, most commonly with preserved ejection fraction (HFpEF), although a drop in ejection fraction can occur in more advanced stages of the disease. The clinical syndrome may present predominant left HF symptoms with pulmonary congestion (dyspnea, orthopnea, paroxysmal nocturnal dyspnea), right HF symptoms (edema, ascites, hepatomegaly, increased abdominal volume, early satiety, severe fatigue), or both sets of symptoms. Cardiac amyloidosis should be considered in the differential diagnosis of HFpEF etiology in older men,^41^ particularly when there is no apparent history of systemic arterial hypertension or the thickness of the interventricular septum increases ≥ 12 mm, which raises the possibility of infiltrative cardiomyopathy.^21^

Syncope and orthostatic hypotension are common symptoms and indicate the presence of dysautonomia. One typical clinical aspect that may raise the suspicion of amyloidosis is the need for dose reduction or discontinuation of antihypertensive drugs in patients previously diagnosed with systemic arterial hypertension, especially beta-blockers and angiotensin-converting enzyme inhibitors/angiotensin receptor blockers.^15^

Amyloid infiltration can also occur, causing cardiac conduction system disease from the early stages, with variable degrees of atrioventricular block, which results in high-risk bradycardia in some cases, requiring pacemaker implantation. Another important change is the hardening of the atrial walls, which leads to high rates of atrial arrhythmias, including atrial fibrillation, as well as atrial thrombi, with cardioembolic stroke being a common clinical manifestation, even in individuals with sinus rhythm. Complex ventricular arrhythmias seem to be frequent in advanced stages of the disease, an aspect that is best documented in AL amyloidosis.

### 4.1. Increased Suspicion of Cardiac Amyloidosis

CA, particularly the ATTR type, is often underdiagnosed due to factors associated with the medical evaluation, in addition to the characteristics of the disease itself, including: fragmented knowledge among different specialties and subspecialties, a scarcity of centers and specialists dedicated to the management of this disease, the mistaken belief that CA is a rare and incurable disease, and the phenotypic and genotypic heterogeneity of ATTR-CA.^42^It should be pointed out that early CA diagnosis is critical, since the prognosis rapidly worsens with continued amyloid protein deposition and increasing organ dysfunction.

Thus, recognizing “red flags” can help diagnose CA in HF patients,^35^^,^^43^ the most relevant of which are summarized in Table 2.

**Table 2:** Clinical clues that should raise suspicion of cardiac amyloidosis in patients with manifestations of heart failure

Clinical history and physical examination
HFpEF, particularly in men > 65 years of age
Intolerance to ACEi/ARB/ARNi and/or beta-blockers
Unexplained LV block with prior pacemaker implantation
Bilateral carpal tunnel syndrome
Spinal canal stenosis
Biceps tendon rupture
Unexplained sensorimotor polyneuropathy (paraesthesia, neuropathic pain, weakness)
Autonomic dysfunction (postural hypotension, alternating diarrhea with constipation, erectile dysfunction)
Spontaneous/minimal trauma periorbital purpura
Macroglossia
Vitreous opacity and pupillary changes
Family history of cardiomyopathy or polyneuropathy
**Imaging examinations**
Infiltrative phenotype on echocardiogram (IVS ≥ 12 mm), biventricular hypertrophy, myocardial hyperrefringence, valve thickening, thickening of the interatrial septum
Concentric thickening of the LV walls with reduced or normal QRS amplitude in proportion to the increase in LV wall thickness
**Combined clues**
Low flow/low gradient aortic stenosis in patients > 60 years of age
Clinical presentation of late-onset hypertrophic cardiomyopathy in patients > 60 years of age

*ACEi: angiotensin-converting enzyme inhibitor; ARB: angiotensin II receptor blocker; ARNi: Angiotensin receptor neprilysin inhibitor; HFpEF: heart failure with preserved ejection fraction; IVS: interventricular septum; LV: left ventricle*.

Bilateral carpal tunnel syndrome, often one of the first indicators of ATTR-CA, is the most common non-cardiac manifestation and can precede symptoms of HF by several years. A recent study found that approximately 50% of individuals with ATTRwt had carpal tunnel syndrome 5 to 7 years prior to diagnosis.^44^ Lumbar stenosis and atraumatic rupture of the biceps tendon have also been identified as clinical manifestations of extracardiac deposition in ATTRwt. Biceps tendon rupture may occur in up to 33% of ATTRwt cases.^45^ On the other hand, macroglossia and periorbital purpura are highly specific to AL-CA, although they occur in only 15% of cases.^46^ Sensorimotor polyneuropathy or dysautonomia in HF patients should raise suspicion of CA.^47^^,^^48^

Other warning signs may emerge from typical changes in routine complementary cardiac examinations, which are covered as specific topics in this document.

It should also be pointed out that CA can often simulate other heart diseases. Amyloidosis should be considered one possible etiology for patients with a hypertrophic cardiomyopathy phenotype, particularly if it developed after 60 years of age. The asymmetrical pattern of myocardial hypertrophy in ATTR-CA patients differs from that of AL-CA patients, which is usually symmetrical. In a study that compared 263 confirmed ATTR-CA patients with 50 AL-CA patients, among the ATTR cases, asymmetric hypertrophy was present in 79%, symmetric hypertrophy in 18%, and no myocardial hypertrophy in 3%.^49^

Older patients with severe low-flow/low-gradient aortic stenosis may have CA in 10% to 15% of cases, with an unfavorable prognosis.^50^

## 5. Additional Diagnostic Examinations

### 5.1. Electrocardiogram

The electrocardiogram (ECG) is an essential test for diagnostic assessment and therapeutic planning, and its interpretation in conjunction with clinical and echocardiographic information is important. Although low-voltage ECG has great specificity in diagnosing myocardial infiltration secondary to CA, this is not the most prevalent finding. A lack of R-wave progression in precordial leads, which simulates an electrically inactive anteroseptal zone (pseudoinfarction pattern) is a much more frequent finding, with a prevalence of 60% to 70% in confirmed CA diagnoses, regardless of the type (Figure 3).

**Figure 3:** Sample electrocardiogram image from a patient with wild-type transthyretin cardiac amyloidosis, showing low voltage in peripheral leads, no R-wave progression in precordial leads V1 to V3 (pseudoinfarction pattern) and first degree atrioventricular block. (Image from the authors’ personal archive)

In ATTR-CA, less than 40% of patients with a biopsy-confirmed diagnosis have low-voltage ECG.^51^

Thus, without low-voltage ECG criteria or signs of left ventricle (LV) overload, diagnostic suspicion of CA should not be ruled out, especially ATTR-CA. Disproportionate voltage in relation to myocardial thickness is also an important warning sign, reaching a prevalence of 73% to 80% in CA patients, regardless of type.^52^^,^^53^

Among cardiac rhythm alterations, atrial fibrillation is more prevalent in ATTR patients, as well as atrioventricular blocks.

### 5.2. Echocardiogram

Echocardiography should be performed in all patients with clinical suspicion of the disease. Classic CA findings are usually present at an advanced stage of the disease and characterize a restrictive, infiltrative-type cardiomyopathy. The dimensions of the LV are not increased, volumes are normal or reduced, and there is a thickening of the ventricular walls. Increased atrial dimensions are common, reflecting early and progressive diastolic dysfunction, with increased filling pressures. Atrioventricular valves may be thickened and mitral and tricuspid regurgitations are functional. There may also be signs suggestive of infiltration of the interatrial septum, as well as an increase in the pulmonary artery systolic pressure. The right ventricle (RV) may also be affected. Pleural and pericardial effusions are very common and, in cases involving intense tissue infiltration, a granular sparkling appearance can be observed (Figure 4)^35^^,^^54^

**Figure 4:** Classic echocardiographic presentation of cardiac amyloidosis. In A, the longitudinal parasternal projection shows a normal-sized left ventricle (LV) with increased wall thickness and a granular aspect in the interventricular septum (green arrow). There are also signs of left atrium (LA) enlargement and mild pericardial effusion (yellow arrow). In B, the apical projection shows large atria, normal-sized ventricles, and increased wall thickness, as well as a granular aspect in the interventricular septum (green arrow). The mitral and tricuspid valves are slightly thickened. LV: left ventricle; RV: right ventricle; LA: left atrium. (Images from the authors’ personal archive).

The LV ejection fraction is normally preserved until the more advanced stages of CA, although the longitudinal contractile function is reduced early.^55^ Quantitatively, systolic function can be assessed in 2D mode by calculating LV stroke volume and ejection fraction, as well as by Doppler-derived techniques such as estimating left ventricular dP/dt.^56^

Assessing the ventricular diastolic filling pattern is essential and often demonstrates some degree of diastolic dysfunction. In the initial phases, alterations compatible with type I diastolic dysfunction can be observed (inversion of the mitral inflow E/A wave ratio, prolongation of the isovolumetric relaxation time, and early diastolic deceleration. In tissue Doppler recording of myocardial velocities, E’ wave deceleration may be observed (Figure 5).

**Figure 5:** Tissue Doppler images of the mitral annulus. There is E’ wave(*) deceleration in both the medial (a) and lateral (b) mitral annulus. Both speeds are below 4 cm/s (right ventricle > 8 cm/s). (Images from the authors’ personal archive).

As the disease progresses, a pseudonormal pattern of diastolic dysfunction (normal E/A wave ratio and normal deceleration time) may develop as a result of increased left atrial pressure.

In more advanced phases of the disease, there is a restrictive pattern of ventricular filling (E/A wave ratio > 2, decreased relaxation time, and an increased deceleration slope of the E-wave).

Analyzing myocardial deformation allows the early identification of signs of myocardial dysfunction in relation to LV ejection fraction.^57^^–^^59^ Global longitudinal strain – a function predominantly performed by the endocardium – is reduced early.^60^ Regional myocardial deformation also frequently presents an apical sparing (“cherry on top”) pattern.^61^^,^^62^ This aspect is best visualized in a parametric (“bulls eye”) image of the LV (Figure 6).

**Figure 6:** Analyses of global and regional LV myocardial deformation in a patient with cardiac amyloidosis, showing the parametric graphical representation (bulls-eye), apical segments in dark red (preserved values), and basal segments in light red (reduced values) in the “cherry-on-top” pattern. (Image from the authors’ personal archive)

Other analyses of myocardial deformation, such as RV systolic function,^63^ atrial function,^65^ and estimating myocardial work,^59^have been applied to CA patients and have shown good diagnostic accuracy.

### 5.3. Cardiac Magnetic Resonance

Cardiovascular magnetic resonance (CMR) allows accurate assessment of myocardial tissue changes in CA.^65^ Classically, the deposition of myofibrils leads to an increase in the thickness of the LV myocardial wall and the interatrial septum, which can be visualized by the morphological techniques of CMR.^66^^,^^67^ Another tissue change is increased total water content in the myocardium, which may be derived from increased extracellular volume, a direct result of protein deposition and the water it attracts through osmosis, as well as from the increased intracellular water in myocytes suffering the cytotoxic effects of the deposition or even from decreased myocardial perfusion (increased distance from the capillaries and/or obstruction by the deposition).

The global increase in myocardial tissue water content leads to an increase in mean hydrogen relaxation time, whether T1 (longitudinal) or T2 (transverse).^68^ However, the most dramatic and relevant myocardial tissue change in CA is the extreme increase in myocardial extracellular volume, which occurs in the clinical stages of the disease, not only due to the deposition of amyloid fibrils, but also to myocardial fibrosis repair.

Thus, the combination of myofibril deposition and interstitial fibrosis can be detected easily and precisely, and can even be quantified by late enhancement (LE) techniques^67^^,^^69^ and by calculating the extracellular myocardial volume.^70^^–^^72^ As an example, normal values for myocardial extracellular space are approximately 25%, while in CA they can reach 60% (mainly in ATTR-CA).^72^^,^^73^

The CMR contrast medium is based on gadolinium, which is bound to a macromolecular chelator that does not allow it to pass through the entire cell membrane. Thus it is distributed exclusively in the myocardial extracellular volume. The distribution pattern in the LE image can raise suspicion of CA (global subendocardial, apical non-involvement of the LV, and distribution outside the coronary vascular territory).^67^

#### 5.3.1. Assessment of Cardiac Morphology and Function

CA can modify the appearance of all cardiac chambers.^74^^,^^75^ Changes in the atrial plane can be observed the initial stages, including dilation and apparent thickening of the interatrial septum, which, in most cases, consists of fat.^74^ In later stages of cardiac impairment, when atrial function decreases, signs of slow flow and thrombi in the left atrial appendage can be seen, which may not appear in cardiovascular magnetic resonance imaging if there are artifacts related to rhythm or if specific series are not used for this purpose.

CA is also commonly associated with increased myocardial thickness, which is, in most cases, more expressive than in cases secondary to hypertension. The thickness of the cardiac muscle is generally greater in ATTR-CA than AL-CA.^52^^,^^76^ The increased thickness may be concentric or eccentric,^74^^,^^75^and may involve the RV.^74^^,^^75^ The ejection fraction may be preserved for a long time, and the earliest changes in ventricular function include diastolic restriction and changes in ventricular strain.^75^^,^^76^

As a result of diastolic dysfunction, it is not uncommon to observe pericardial or pleural effusions.^74^ Assessing morphological characteristics with cardiovascular magnetic resonance imaging (Figure 7) is helpful and can suggest the diagnosis. However, tissue characterization is usually performed with LE and T1 mapping techniques, which we will discuss below in other sections.

**Figure 7:** Example of cardiovascular magnetic resonance imaging of transthyretin amyloidosis, showing pericardial effusion (A, yellow arrow and C), increased atrial dimensions (A and B), and increased interatrial septum (A and B, red arrows) and ventricular wall thickness (A to C, white arrows). Diastolic function is reduced, but contractility may be preserved until the later stages of the disease. (Image from the authors’ personal archive).

#### 5.3.2. Assessment with Late Enhancement

The LE technique after gadolinium contrast injection has been widely recognized as a pillar of CA imaging diagnosis.^77^^,^^78^ When crossing the interstitial space in normal tissue, gadolinium contrast medium is not delayed and the agent quickly diffuses, leaving normal tissue dark. When there is amyloid deposition in the interstitial space, it begins to slow the transit of the gadolinium-based contrast agent, and the myocardium “shines” in the dedicated sequences, for which an anatomopathological correlation has been demonstrated.^65^ Subendocardial, transmural and focal patterns of amyloid infiltration have been described, the latter being less frequent (Figure 8).^67^ A recent meta-analysis found that LE had a sensitivity and specificity of 85% and 92%, respectively, for amyloidosis.^79^

**Figure 8:** Example of a patient with ATTR transthyretin with concentric hypertrophy of the left ventricle and enlargement of both atria in cine images (A to C). Late enhancement shows a predominantly diffuse transmural pattern (red arrows, D and E). (Image from the authors’ personal archive).

In a series of 250 patients with different forms of amyloidosis, LE with a transmural pattern was associated with a 5.4 times greater risk of death (CI: 2.1-13.7; p < 0.0001).^80^ In addition, LE has an incremental prognostic effect for cardiac markers in AL-CA^81^ or in isolation.^82^

#### 5.3.3. T1 Mapping

Different groups have investigated the utility of cardiovascular magnetic resonance-derived T1 maps to improve diagnostic and prognostic performance in CA.^70^^,^^71^^,^^73^^,^^83^^,^^84^ Both native T1 data, which do not require a contrast agent, as well as extracellular volume data have effectively identified patients with CA, who show markedly higher native T1 and extracellular volume values than healthy controls.^70^^,^^73^^,^^83^These changes can be detected before LE.^70^^,^^72^^,^^73^ Therefore, combining native T1 mapping and extracellular volume measurements can help determine the amyloid burden and confirm the diagnosis of CA.^85^^,^^86^

## 5.4. Cardiac Scintigraphy with Bone-seeking Radiotracers

Technetium-99m-labeled bisphosphonate-derived radiotracers, originally developed for bone imaging, have found a new role as a non-invasive diagnostic tool for ATTR-CA.^1^^–^^7^ Bone radiotracers safely allow non-invasive diagnosis of ATTR-CA once the presence of monoclonal gammopathy is excluded.^91^

The main ^99m^Tc-labeled bone radiotracers used in ATTR-CA diagnosis are ^99m^Tc-pyrophosphate, ^99m^Tc-DPD (3,3-diphosphono-1,2-propanedicarboxylic acid), and ^99m^Tc-HMDP (99mTc-labeled hydroxymethylene diphosphonate).^1^^–^^7^ ^99m^Tc-pyrophosphate is the only one of these available in Brazil. It should be pointed out that although ^99m^Tc-MDP (99mTc-labeled methylene diphosphonate) has proven efficient for bone scintigraphy, it has low sensitivity for diagnosing ATTR-CA and should not be used for this purpose.^89^

Although the structural component of the amyloid deposition to which ^99m^Tc-pyrophosphate binds in the heart is unknown, it is widely accepted that a calcium-dependent uptake mechanism is involved.^93^ Several binding sites have been found in animals: microcalcifications, calcium deposits, intracellular pyrophosphate, and intracellular macromolecules. The mechanism of ^99m^Tc-pyrophosphate uptake in the myocardium is probably related to the presence of microcalcifications.^88^ ATTR-CA involves more microcalcifications than AL-CA and has greater ^99m^Tc-pyrophosphate uptake, while AL-CA has little or no affinity for bone radiotracers. In addition, ATTR-CA has a more indolent evolution, providing more microcalcifications and, consequently, greater radiotracer accumulation.

Although the echocardiogram and cardiac magnetic resonance findings may be indicative of CA, they cannot differentiate ATTR-CA from AL-CA. This is the main advantage of ^99m^Tc-pyrophosphate cardiac scintigraphy: it is a simple, easy, widely available method with low dosimetry that can differentiate ATTR-CA from AL-CA noninvasively and with high specificity, and thus guide treatment. This differentiation is useful, since AL-CA and ATTR-CA have completely different prognostic and therapeutic implications.

The role of bone radiotracers in ATTR diagnosis was recently re-evaluated by an international group of several centers with expertise in CA: in a cumulative analysis of 1,217 patients, 867 with biopsy-confirmed amyloidosis and 360 with non-amyloid cardiomyopathy, scintigraphy was highly sensitive (99%) and specific (86%) for ATTR-CA.^19^ This study further demonstrated that the combined finding of positive bone radiotracer scintigraphy in patients with no evidence of detectable monoclonal protein in urine or serum (serum free light chain analysis and electrophoresis with immunofixation) was 100% specific for ATTR-CA, leading the authors to conclude that scintigraphy allows for accurate detection without the need for cardiac biopsy.^19^ Another recent study with pooled cases from three U.S. centers showed that, among a total of 171 patients (121 ATTR, 34 AL, and 16 non-amyloid HFpEF), ^99m^Tc-pyrophosphate had an 88% sensitivity and 88% specificity for ATTR when only visual assessment was used (score ≥ 2).^94^ When semiquantitative analysis was used (heart/contralateral ratio > 1.6), its sensitivity and specificity were 91% and 92%, respectively, for detecting ATTR. Furthermore, when considering all variables, a heart/contralateral ratio ≥ 1.6 was predictive of worse survival in ATTR-CA patients.^94^ Vraniam et al.^95^also demonstrated that, in patients with suspected CA, the intensity of cardiac ^99m^Tc-pyrophosphate uptake was predictive of overall mortality and hospitalization for HF. In these studies, combined assessment of the intensity of radiotracer uptake in the myocardium (heart/contralateral ratio) with anatomical, functional and biomarker variables improved risk stratification.

### 5.4.1. Recommended Technical Aspects for Image Acquisition

No preparation required. Images are obtained after intravenous administration of 10 to 25 mCi (370 to 925 Mbq) of ^99m^Tc-pyrophosphate (dosimetry: 3.2 mSv for the whole body for 15 mCi). Flat and single photon emission computed tomography (SPECT) images of the chest are taken 1 and 3 hours after administration of the radiopharmaceutical.

- *Planar chest images:* can be obtained in the anterior, left anterior oblique, and left lateral projections using ^99m^Tc photopic (140 keV, 15% window), low energy/high resolution collimator and a 256 × 256 matrix, 500,000-750,000 counts.
- *SPECT chest images:* obtained with a 128 × 128 matrix (64 × 64 is acceptable), 180-degree rotation from right anterior oblique to left posterior oblique (360-degree is acceptable), 1 image every 3 to 6 degrees. If available, SPECT/CT images (SPECT combined with computed tomography) provide greater confidence for interpreting the images.
- *Minimum recommended images:* 1 h SPECT and 1 h and 3 h flat images in the anterior projection. The use of SPECT images is recommended to differentiate diffuse uptake of the radiopharmaceutical in the myocardium from its persistence in the blood pool, focal uptake by myocardial infarction, and bone overlay. In addition, 1 h and 3 h flat images are useful for quantifying and monitoring the blood pool washout, which is variable, eg, much slower in patients with renal failure.

### 5.4.2. Image Analysis

- *Semiquantitative analysis (1 h planar image):* For ^99m^Tc-pyrophosphate, the semiquantitative analysis was defined as the ratio between uptake in the cardiac projection and uptake in the contralateral hemithorax, measured in a planar 1 h image in the anterior view. To do this, a circular (or elliptical) area of interest is drawn over the cardiac projection without including the sternum, avoiding the inclusion of both the adjacent lung and areas of focal uptake in the costal arches. An identical (“mirror”) area of interest is placed in the contralateral hemithorax following the same procedure. The count within the cardiac area of interest is divided by the count in the contralateral area of interest, thus obtaining the heart/contralateral (H/CL) ratio. H/CL ≥ 1.5 at 1 h identifies ATTR-CA with high accuracy if systemic AL-CA has been excluded^90^^,^^91^ (Figure 9).
- *Visual graduation (3 h image):* visual grading is performed by comparing cardiac uptake with physiological uptake in adjacent costal arch, and can be performed with planar images in the anterior projection, in SPECT images, or even in whole-body images obtained 3 h after injecting the radiopharmaceutical. Uptake intensity is defined as grade 0 (no myocardial uptake), grade 1 (myocardial uptake lower than that of adjacent costal arch), grade 2 (uptake similar to that of costal arch), and grade 3 (greater than that of the costal arch). Grades 2 or 3 are strongly suggestive of ATTR if monoclonal gammopathy has been excluded (Figure 9).

**Figure 9:** ^99m^Tc-pyrophosphate cardiac scintigraphy in patients with suspected cardiac amyloidosis (flat images in anterior chest view taken 3 h after administration) showing negative and positive transthyretin (ATTR) amyloidosis cases. Visual analysis: images on the left: negative ATTR cases (grades 0 and 1); images on the right: positive ATTR cases (grades 2 and 3). Semi-quantitative analysis: on the left, negative ATTR case (heart/contralateral ratio = 1.2 – in this case, the SPECT images showed activity in the blood pool and not in the heart walls); on the right, positive ATTR case (heart/contralateral ratio = 1.6 –SPECT imaging confirmed uptake in the left ventricle walls).

In visual interpretation, cardiac uptake equal to (grade 2) or greater (grade 3) than that of the costal margins is important for ATTR diagnosis. In semiquantitative analysis, heart/contralateral count ratios ≥ 1.5 in 1 h images and ≥ 1.3 in 3 h images are considered highly suggestive of ATTR when confirmed by SPECT images. (Images from the authors’ personal files)

### 5.4.3. False-Positive ATTR

The operational characteristics of bone scintigraphy are very favorable for use in clinical diagnosis, with a specificity of 100% for ATTR when the uptake is grade 2 or 3 and there is no monoclonal gammopathy. However, it must be pointed out that failure to exclude monoclonal gammopathy (either due to inappropriate use or to misinterpretation of laboratory tests) entails a risk of inaccurate diagnosis. The most common cause of ATTR misdiagnosis is AL-CA. Recent studies indicate that up to 22% of patients with AL-CA may have grade 2 or 3 uptake in ^99m^Tc-pyrophosphate scintigraphy.^6^ It should also be pointed out that SPECT imaging is crucial to differentiate abnormal myocardial uptake from residual uptake in the blood pool (Figure 10). Table 3 lists the main causes of false-positive diagnosis with ^99m^Tc-pyrophosphate scintigraphy.

**Table 3:** Causes of false-positive ^99m^Tc-pyrophosphate scintigraphy (myocardial ^99m^Tc-pyrophosphate uptake that is not associated with amyloid transthyretin)

1. Light-chain cardiac amyloidosis
2. Capture in blood pool (planar images)
3. Rib fractures (planar images)
4. Myocardial infarction (acute or subacute)
5. Hydroxychloroquine cardiotoxicity
6. Rare forms of cardiac amyloidosis

*Source: adapted from Hanna et al*.^91^

**Figure 10:** Illustrative images of a 76-year-old male patient with ATTRv (VAL30MET) cardiac amyloidosis. Cardiac scintigraphy with ^99m^Tc-pyrophosphate (flat images A and B) showed intense radiotracer uptake in the 1 h and 3 h images. Visual analysis (A) showed grade 3 uptake in both 1 h and 3 h images (myocardial uptake intensity greater than that of the costal margins). The semiquantitative analysis (B) showed a 2.1 heart/contralateral ratio at 1 h 2.0 and at 3 h. Single photon emission computed tomography (SPECT) showed radiopharmaceutical uptake in all left ventricle walls and confirmed right ventricle involvement, as shown in the flat images (arrows). The ^99m^Tc-pyrophosphate cardiac scintigraphy findings are highly suggestive of ATTR-CA. (Images from the authors’ personal files).

Finally, scintigraphy is not recommended for patient follow-up, since there is no current evidence of a correlation between a change in image pattern and disease progression or response to treatment.^87^^,^^90^

Table 4 summarizes the main findings and practical guidelines for image acquisition and analysis in CA diagnosis.

**Table 4:** Summary of the main findings suggestive of cardiac amyloidosis in complementary examinations

Examinations - results suggestive of cardiac amyloidosis	Practical tips - diagnostic details
Electrocardiogram	
Pseudoinfarction pattern	A classic low voltage pattern is common in AL-CA (up to 70% of cases) but uncommon in ATTR-CA (only 30% of cases)
QRS voltage disproportionate to degree of wall thickening	The ECG results should be interpreted together with clinical and echocardiographic data
Low voltage of QRS complexes	BAV or AF can be an initial manifestation of CA
Atrial arrhythmias - atrial fibrillation	
Conduction disorders	
Echocardiogram	
Increased ventricular thickness (IVS > 12 mm)	In addition to structural and functional information from conventional echocardiography, myocardial deformation analysis is important to identify myocardial dysfunction, even with normal EF and an apical sparing pattern
Granular/hyperrefringent appearance of thickened myocardium	
Thickening of both the RV and LV walls+	
Biatrial dilation	
Valve and thickening of the interventricular septum	
Diastolic dysfunction (restrictive pattern)	
Longitudinal myocardial deformation with relative apical sparing pattern	
Cardiac Magnetic Resonance	
High native T1 values	In addition to structural and functional cardiac information, which can be very suggestive, late enhancement information, T1 mapping and extracellular volume are important indicators of CA
Marked increase in extracellular volume	CMR cannot differentiate between AL-CA and ATTR-CA
Diffuse transmural or subendocardial delayed enhancement	CMR may help identify other forms of infiltrative cardiomyopathy
Late enhancement in atrial walls	
Increased RV/LV wall and interatrial septum thickness	
Cardiac scintigraphy with bone radiotracer	
Myocardial uptake ≥ bone uptake (grade 2 or 3)	In Brazil, only 99mTc-pyrophosphate is available for this purpose
Heart/contralateral uptake ratio ≥ 1.5	In the absence of light chains, positive 99mTc-pyrophosphate scintigraphy diagnoses ATTR-CA without the need for EMB
99mTc-pyrophosphate uptake in heart walls confirmed by SPECT imaging
	Up to 30% of AL-CA may show positive scintigraphy
	Negative 99mTc-pyrophosphate scintigraphy does not rule out CA

*AF: atrial fibrillation; BAV bicuspid aortic valve; EMB: endomyocardial biopsy; CA: cardiac amyloidosis; CMR: Cardiac Magnetic Resonance; ECG: electrocardiogram; EF: ejection fraction; IVS: interventricular septum; GLS: global longitudinal strain; LV: left ventricle; SPECT: single photon emission computed tomography; RV: right ventricle*.

## 5.5. Biomarkers

No specific laboratory marker can be used to diagnose CA.

Troponin and natriuretic peptides have been found useful for assessing cardiac damage due to amyloidosis and are non-invasive, accessible, and relatively low-cost diagnostic aids.^96^ When these biomarkers detect persistent changes, it is a warning sign of cardiac damage due to amyloidosis.

### 5.5.1. Natriuretic Peptides

Analysis of data from the THAOS (Transthyretin Amyloidosis Outcomes Survey) registry showed that natriuretic peptide levels can be used as a diagnostic aid, with higher values observed in mutations associated with amyloid cardiomyopathy, such as V122I and late-onset V30M, than in those predominantly associated with neurologic manifestations, such as early-onset V30M. Higher levels of biomarkers were also observed in ATTRwt than ATTRv. However, it was observed that even in patients with a predominantly neurological phenotype, 45% to 90% had altered levels of these biomarkers, indicating some degree of subclinical myocardial involvement.^97^

Higher NT-proBNP values have been observed in AL-CA than ATTR-CA. This is because amyloidogenic light chains modulate p38 mitogen-activated protein kinase, which directly promotes NT-proBNP expression. Thus, despite the same degree of hemodynamic changes in both forms of amyloidosis, serum levels of NT-proBNP may be higher in AL-CA.^98^

Of note, patients with HFpEF due to ATTR have disproportionately high NT-ProBNP values with respect to HF severity compared to patients with non-amyloid HFpEF.^99^

### 5.5.2. Troponins

Mild and persistent elevation of troponin levels is frequently observed and suggests subclinical myocardial damage in several non-ischemic cardiomyopathies.^100^ However, it has been reported that levels are higher in CA than in other forms of cardiomyopathy.^21^ A study of patients with hypertrophic cardiomyopathy who underwent endomyocardial biopsy identified markedly higher troponin levels in CA patients than in those with amyloid-free heart disease and had high diagnostic sensitivity.^101^

Several mechanisms have been postulated to explain the elevated troponin levels in these patients: myocardial ischemia, increased wall stress, direct myocyte damage by inflammatory cytokines and/or oxidative stress, neurohormonal activation, and microvascular dysfunction in heart failure. Microvascular dysfunction in amyloidosis is presumably caused by interstitial and perivascular deposition, increased ventricular filling pressure, and endothelial dysfunction due to immunoglobulin-induced toxicity in AL-CA. In addition, it has also been found that light chains have a direct cardiotoxic effect, regardless of extracellular deposition of fibrils, which might explain the higher troponin levels in AL-CA than in ATTR-CA, in which mild and persistent elevation is usually observed.^101^

It is important to point out that many ATTR patients have comorbidities, such as ischemic cardiomyopathy, which can cause altered troponin values. Therefore, troponin levels should not be used to rule out or confirm cardiac involvement. Rather, they should serve as a potential warning sign for the disease, which can be better evaluated through more specific tests.

### 5.5.3 New Biomarkers

A number of other biomarkers have been studied, some with high specificity for amyloidosis subtypes, such as retinol-binding protein 4 for hereditary amyloidosis through the Val142Ile mutation. However, further studies are necessary, as is the commercial availability of tests for routine analysis.^102^

## 6. Rational Diagnostic Approach to Cardiac Amyloidosis

Recent evidence indicates that CA, particularly the ATTRwt form, is more prevalent than previously estimated, and this is due in part to widespread underdiagnosis and the fact that this disease mimics other heart diseases, such as hypertrophic cardiomyopathy, non-amyloid HFpEF, and low-flow/low-gradient aortic stenosis.^42^ These factors indicate that high suspicion of the disease in different clinical scenarios is needed for a more rational diagnostic process.^54^ Figure 11 shows a diagnostic algorithm for CA, whose steps are discussed below. The main recommendations and classes of evidence for diagnosis are listed in Table 5.

**Table 5:** Recommendations for cardiac amyloidosis diagnostics

Indication	Recommendation class	Level of evidence (references)
Begin CA investigation in patients with signs and symptoms of HF and one or more warning sign of the disease (see Table 2)	I	C
Whenever available, perform echocardiography with analysis of LV myocardial strain, and/or CMR with LE and T1 mapping for patients with suspected CA	I	B (ref. 60, 61, 62)
For suspected CA, continue the diagnostic investigation with serum and urine immunofixation tests and kappa/lambda light chain ratio measurement to assess the possibility of AL-CA	I	B (ref. 19)
When available, perform cardiac scintigraphy with a bone-seeking radiotracer* to diagnose ATTR-CA, after AL type has been excluded (light-chain negative)	I	B (ref. 19, 94, 95)
Perform EMB to diagnose CA in cases with high clinical suspicion of the AL form (light- chain positive) when the peripheral tissue biopsy is negative	I	B (ref. 108)
Perform amyloid protein typing in EMB using immunohistochemistry or mass spectroscopy, when available, when the CA type is indeterminate or conflicts with the clinical suspicion	I	C
Perform EMB to confirm CA diagnosis in suspected cases of ATTR when bone scintigraphy is not available	I	B (ref. 19)
Perform EMB to confirm CA diagnosis when there is high suspicion of ATTR, but bone scintigraphy is indeterminate	I	C
Perform genetic sequencing in patients diagnosed with ATTR to differentiate ATTRv from ATTRwt	I	B (ref. 33)

* *Radiotracers that can be used for this purpose include ^99m^Tc-pyrophosphate, ^99m^Tc-DPD (3,3-diphosphono-1,2-propanedicarboxylic acid) and ^99m^Tc-HMDP (hydroxymethylene diphosphonate); of these, only ^99m^Tc-pyrophosphate is available and approved for use in Brazil. AL: light-chain amyloidosis; ATTR: transthyretin amyloidosis; ATTRv: hereditary ATTR; ATTRwt: wild-type ATTR; CA: cardiac amyloidosis; EMB: endomyocardial biopsy; HF: heart failure; LV: left ventricle; LE: late enhancement*

**Figure 11:** Flowchart for diagnosing cardiac amyloidosis.

The ***first and most important step*** is clinical suspicion, which is based on clinical history, a physical examination (see Table 2) with findings suggestive of CA in ECG, echocardiogram, and CMR (see Table 4).

***Thus, in cases with high clinical suspicion, an investigation of immunoglobulin monoclonal light chains should be performed*** for effective screening of AL-CA, given that an AL-CA diagnosis is a medical emergency, and treatment delay should be avoided, since it is associated with a markedly worse prognosis.

Protein electrophoresis is not an adequate screening test since this method may not detect the monoclonal component in blood and/or urine. Thus, it is important to carry out immunofixation in blood and urine, which increases the detection sensitivity for clonal light chains to around 90%.^103^ Adding the serum free light chain ratio, which detects an abnormal relationship between kappa/lambda chains (> 1.65 or < 0.26), increases the detection sensitivity to > 99%.^103^^,^^104^ Therefore, electrophoresis with immunofixation in blood and urine associated with free light chain ratio analysis represents the best non-invasive method for detecting clonal light chains, which indicate the presence of AL.

The patients with positive results for monoclonal light chains must be referred to a hematologist (following the ***hematological route*** on the algorithm), and a tissue biopsy must be performed, which is fundamental for confirming amyloid protein deposition and definition of the therapeutic strategy.

### 6.1. Hematological Route

#### 6.1.1 Hematologist Participation and Peripheral Tissue Biopsy

An AL-CA diagnosis must be confirmed by biopsy. Abdominal fat biopsy, a simple and safe method, is preferred initially.^105^

Congo red staining with birefringence under polarized light is used to determine tissue amyloid protein. In cases of systemic amyloidosis, ie, affecting numerous organs or tissues, abdominal fat biopsy with this stain has a sensitivity of 60-80% and a specificity of 90-100% for diagnosing amyloidosis,^106^ as well as a strong association with the total body load of amyloid deposition.^37^ However, in cases of localized amyloidosis, ie, restricted to one organ or tissue, a subcutaneous fat tissue biopsy is seldom positive.^107^ In general, there is a higher chance of positive extracardiac biopsy results when the site is abdominal fat and the amyloidosis is AL. AL is followed by ATTRv and ATTRwt in this regard.^37^ In a series of 131 patients whose ATTR-CA diagnosis was confirmed by endomyocardial biopsy, the abdominal fat biopsy was positive in 67% of ATTRv patients but only in 14% of ATTRwt patients.^8^ Therefore, although abdominal fat is the preferred initial site for extracardiac biopsies, a negative result should not exclude the diagnosis, and an endomyocardial biopsy should be performed.^37^ In these cases, biopsy of the affected organ has 100% sensitivity and specificity.

It should be pointed out that although Congo red staining can confirm amyloid infiltration in tissue, it cannot identify the type of precursor protein. In addition, 40% of ATTR patients may have a monoclonal gammopathy of undetermined significance, presenting positive results in a monoclonal light chain analysis.^109^

In light of these aspects, since identifying the amyloid deposition type is fundamental for appropriate treatment, immunohistochemistry or, preferably, laser microdissection and mass spectrometry of the amyloid biopsy material must be performed. Immunohistochemistry remains the most widely available method for identifying the deposition type. However, when the amyloidosis is light chain, the results are not always conclusive; they may be positive for more than one type of antiserum, usually TTR and kappa or lambda chain. Thus, mass spectrometry has become the new gold standard for identifying amyloid deposition type.^37^^,^^54^^,^^110^

For ***negative monoclonal light chain results***, ie, when ATTR is more likely, although other rare forms of CA may also be diagnosed, the investigation should follow the ***cardiological route***, taking two sub-routes according to the availability of scintigraphy with bone markers.

### 6.2. Cardiological Route

When ***scintigraphy with bone tracers (sub-route 2A)*** is available and monoclonal light chains are absent, grades 2 or 3 cardiac uptake (equivalent to or greater than that of the costal archs) and a ≥ 1.5 H/CL uptake ratio, with SPECT imaging showing that the increased uptake is in the ventricular walls, confirms ATTR-CA without the need for endomyocardial biopsy. TTR gene sequencing should then be performed to determine whether the ATTR is hereditary or wild type.

Differentiating between hereditary and wild-type ATTR has prognostic and therapeutic implications and is also important for family screening and genetic counseling.

When cardiac scintigraphy with bone tracers is negative and is associated with an absence of monoclonal light chains, CA is unlikely. However, when clinical suspicion persists, based mainly on the results of other imaging methods highly suggestive of amyloidosis, endomyocardial biopsy can have a relevant diagnostic role and should be performed. Such cases could indicate ATTRv involving mutations and amyloid deposits, to which bone tracers do not adhere, such as early-onset V30M and P64I, in addition to other unusual types of amyloidosis.^111^

When ***bone scintigraphy is unavailable (sub-route 2B)***, endomyocardial biopsy is recommended to clarify the diagnosis.

## 7. Prognosis and Staging

### 7.1. AL-CA

AL-CA patients have faster-forming and more toxic amyloid deposits than ATTR-CA patients. As a consequence, monthly increases of 1.45 to 2.16 mm can be observed in myocardial thickness, which are associated with higher elevations of biomarkers, such as troponin and BNP,^112^ as well as the development of HF symptoms and death within an average of 6 months after diagnosis.^113^

Recent technological and laboratory advances indicate that the main prognostic determinant in AL-CA is the extent of amyloid deposition in the heart.^114^ The most widely used staging criteria were developed by the Mayo Clinic (shown in Table 6), which consider individuals to be at higher risk when troponin T ≥ 0.025 ng/mL, NT-ProBNP ≥ 1,800 pg/ml, and the difference between the light chains ≥ 18 mg/dL.^115^

**Table 6:** Prognostic staging of light-chain amyloidosis according to revised Mayo Clinic criteria^115^

Biomarker cut-off values	Staging (number of biomarkers with high values)	Mean survival
Troponin T ≥ 0.025 ng/mL	Stage I: no biomarkers	94 months
NT-proBNP > 1,800 pg/mL	Stage II: 1 biomarker	40 months
dFLC > 18 mg/dL	Stage III: 2 biomarkers	14 months
	Stage IV: 3 biomarkers	6 months

*dFLC: difference in serum free light chains; NT-proBNP: N-terminal pro-B-type-natriuretic peptide*.

Additionally, echocardiographic findings such as a marked increase in wall thickness, diastolic dysfunction, LV dysfunction, valve thickening, and reduction in LV global longitudinal strain, combined with BNP and troponin biomarkers and hematological status, are predictors of higher mortality.^116^ Myocardial elastography, a new non-invasive myocardial assessment technique that assesses heart stiffness, has shown a good correlation with ventricular mass, myocardial thickness, biomarkers such as BNP, filling pressures, worsening functional class, and diastolic dysfunction.^117^

CMR also provides data that correlates with survival in CA patients.^84^^,^^119^^–^^121^ The presence and extent of myocardial fibrosis, detected by late gadolinium enhancement, indicate poor prognosis.^118^^–^^119^ A recent study found that T1 mapping measurements > 1,044 ms and extracellular volume measurements (calculated in the equilibrium phase) > 0.45 are associated with 5.84 and 3.48 times higher cardiovascular mortality, respectively.^9^^,^^10^

Biomarkers not only play a role in staging these patients, but also enable treatment response assessment, which is always a challenge in clinical practice, especially for AL-CA, since chemotherapy can be an additional factor in myocardial damage. What has been well established in the literature is the agreement between lower NT-proBNP levels and hematologic response to treatment, as well as improvement in New York Heart Association (NYHA) functional class. The reverse is also true, such as increased levels of NT-proBNP and troponin and reduced ejection fraction. (see Table 9 regarding AL-CA treatment)

### 7.2. ATTR-CA

ATTR-CA has a more benign profile and is associated with longer survival than AL-CA.^121^ In addition to the specific type of amyloid deposition, other factors such as genotype, clinical data, laboratory biomarkers, and radiological findings can guide therapeutic planning and determine prognosis.^13^

In cases of hereditary ATTR, the genetic profile determines the clinical phenotype and evolution of the disease, although the gene’s distribution may vary in different populations. Thus, the survival time for pathogenic variants with predominantly myocardial involvement, such as V122I, is shorter than for ATTRwt and mixed or predominantly neuropathic variants such as V30M.^20^^,^^122^^,^^123^

From a clinical point of view, the onset and duration of HF symptoms have prognostic value.^124^ It has been determined that the longer the clinical decompensation and the more advanced the NYHA functional class, the lower the survival.^125^^,^^126^ The median survival observed for each functional class is: I = 4.6 years, II = 4.1 years, III = 2.1 years and IV = 1.3 years. Although sex, age at diagnosis, and comorbidities such as aortic valve disease or tachyarrhythmias do not have a direct effect on mortality, a greater association with unfavorable cardiac outcomes has been observed in older men with associated diseases.^127^

The results of inexpensive, quick, and easy-to-interpret laboratory tests have been used in different proposals for staging the disease, being closely correlated with life expectancy. (Table 7).^128^^–^^130^ Serum levels of troponins T and I, NT-proBNP, and glomerular filtration rate reflect the toxicity of amyloid deposition in target organs by direct action, as well as oxidative stress and inflammation processes.^131^ Recommendations for CA risk stratification/staging and treatment response monitoring are shown in Table 8.

**Table 7:** ATTR amyloidosis staging proposals involving biomarkers

Staging proposal	CA form	Biomarker cutoff points	Staging	Mean survival (months)
Grogan et al. (2016)	ATTRwt	NT-proBNP > 3,000 ng/L Troponin-T > 0.05 µg/L	Stage I NT-proBNP < 3,000 ng/L cTnT < 0.05 µg/L	Stage I = 66
			Stage II NT-proBNP or cTnT above the cutoff point	Stage II = 40
			Stage III NT-proBNP and cTnT above the cut-off point	Stage III = 20
Gillmore et al. (2018)	ATTRwt ATTRv	NT-proBNP > 3,000 ng/L eGFR < 45 mL/min	Stage I NT-proBNP ≤ 3,000 ng/L eGFR > 45 mL/min	Stage I = 69.2
			Stage II NT-proBNP > 3,000 ng/L ^OR^ eGFR < 45 mL/min	Stage II = 46.7
			Stage III NT-proBNP > 3,000 ng/L ^AND^ eGFR < 45 mL/min	Stage III= 24.1

*ATTR transthyretin amyloidosis; ATTRwt: wild-type transthyretin amyloidosis; cTnT cardiac troponin T level; eGFR: estimated glomerular filtration rate; ATTRv: hereditary ATTR amyloidosis; LVEF: left ventricular ejection fraction; NT-proBNP: N-terminal pro–B-type natriuretic peptide*.

**Table 8:** Recommendations for risk stratification/staging of cardiac amyloidosis and monitoring treatment response and/or disease progression

Recommendation	Recommendation class	Level of evidence(references)
Stratify the risk of CA patients using validated staging systems, including biomarkers such as BNP/NT-ProBNP and/or troponins	I	B (ref. 128-130)
Monitor CA progression and/or response to specific treatments using echocardiogram or cardiac magnetic resonance and biomarkers**	I	C

** *The periodicity of the evaluation depends on the type of amyloidosis and the patient’s clinical evolution*.

Imaging findings, such as echocardiography, cardiac scintigraphy with technetium-pyrophosphate (^99m^Tc-PYP), and CMR, provide complementary data that facilitate prognostic stratification.^9^^,^^80^^,^^94^^,^^132^^–^^134^ (Table 9). The increasing quality of the images, associated with greater quantitative detailing of amyloid deposits, has reduced the need for myocardial biopsy as a predictor of disease.^9^

**Table 9:** Imaging parameters that have prognostic value in transthyretin cardiac amyloidosis

Echocardiogram	^99m^Tc-pyrophosphate scintigraphy	Cardiac Magnetic Resonance
↓ LVEF	H/CL ratio ≥ 1.6	TAPSE
↓ MCF		↓ Indexed ejection volume
↓ GLS		↑ Late gadolinium enhancement
Apical sparing		↑ Extracellular volume
↓ ***SVI***^(17)^		↑ Native T1

*LVEF: left ventricular ejection fraction; MCF: myocardial contraction fraction (MCF = SV/ LV volume); GLS: global longitudinal strain; SVI: stroke volume index; H/CL: heart-to-contralateral ratio; TAPSE: tricuspid annular plane systolic excursion*.

## 8. Treatment

CA treatment consists of specific measures aimed at reducing or preventing the progression of amyloid fibril deposition. These measures will be addressed separately for AL-CA and ATTR-CA. General measures are also needed to manage the resultant clinical and hemodynamic abnormalities, including heart failure and cardiac rhythm disturbances, which apply to both AL-CA and ATTR-CA.

### 8.1. Specific Therapy for AL-CA

AL-CA is caused by the aberrant production of kappa or lambda immunoglobulin light chains (*kappa* or *lambda*). Thus, specific treatment is based on eliminating this light chains production by eradicating plasma cell clones in bone marrow. The rapid reduction and, ideally, normalization of light chain levels (hematologic response) is a primary goal in AL-CA treatment. The reversal of damage to tissues and organs affected by amyloid deposits (organ response) is the second major objective of treatment. Further goals include improved quality of life and overall survival.^135^

After the diagnosis of systemic AL-CA has been confirmed, the therapeutic plan must be defined by the hematologist. However, the identification and management of organ dysfunction is essential, as is multidisciplinary work, including joint follow-up with a cardiologist and other specialists (see Table 12). Cardiac involvement is the main prognostic factor in AL-CA, determining not only survival, but also tolerance to cytotoxic treatment. The impact of cardiac impairment on survival has been well established in the literature, and validated staging systems can help with patient risk stratification. These systems are used together with other parameters to determine therapeutic strategies.^135^

Survival in AL-CA is related to the production of amyloidogenic light chains and target organ damage, especially to the heart. Thus, staging includes biomarkers related to these factors,^104^^,^^115^^,^^136^as described above (see Table 5).

The value of this prognostic stratification has been confirmed in individuals treated with autologous hematopoietic stem cell transplantation (HSCT), as well as in those treated without this procedure. The median survival for stage I, II, III, and IV patients is 55, 19, 12 and 5 months, respectively, in patients who did not receive or failed HSCT, and 97, 58 and 22 months, respectively for stages II, III and IV, in patients who underwent HSCT.^115^

Low-risk patients are candidates for high-dose chemotherapy followed by autologous HSCT (Table 12). This is considered the most effective current strategy to eradicate plasma cell clones.^135^ Intermediate-risk patients usually receive chemotherapy at conventional doses and, if they show clinical and laboratory improvement, they can become candidates for autologous HSCT. Finally, older or frail patients with multiple affected organs or patients with advanced cardiomyopathy are treated with chemotherapy at adjusted doses and have a poor prognosis, since they generally cannot tolerate the treatment.^135^

Three months after the end of treatment, the hematological and organ responses must be evaluated with specific tests for monoclonal gammopathy and each affected organ. The hematological and organ response criteria are summarized in Tables 10 and 11.^136^^,^^137^

**Table 10:** Criteria for determining hematological response to treatment

**Complete response:** normalization of the serum free light chain ratio, immunofixation in blood, and negative urine (no clonal component)
**Very good partial response:** reduction in the difference between involved and uninvolved free light chains to < 40 mg/L
**Partial response:** > 50% reduction in the difference between involved and uninvolved free light chains
**No response:** partial response not obtained
**Progressive disease:** free light chain increase of 50% or to > 100 mg/L. In patients with a complete response, the reappearance of a clonal component in immunofixation or free light chains; in patients with a partial response, a 50% increase in the monoclonal component in blood or urine

**Table 11:** Criteria for determining organ response to treatment

**Cardiac response:** > 30% reduction and > 300 ng/L in patients with NT-proBNP ≥ 650 ng/L, or functional improvement of at least two classes according to New York Heart Association criteria
**Renal response:** decrease in proteinuria ≥ 50% (reduction of at least 0.5 g/24 h) at 6 months without a ≥ 25% worsening in estimated glomerular filtration rate. Recent retrospective analyses suggest that a ≥ 30% reduction in proteinuria is associated with better prognosis, although this criterion has not yet been incorporated into formal recommendations^18^
**Liver response:** ≥ 50% reduction in alkaline phosphatase and ≥ 2 cm reduction in liver size
**Peripheral nervous system:** improvement in nerve conduction speed according to electroneuromyography

Even when a complete hematological response occurs, dysfunction improves more slowly, and may take months or even years to appear after the light chain ratio has been normalized.^19^

#### 8.1.1. Treating Patients Eligible for Autologous Hematopoietic Stem Cell Transplantation

Only 20% of patients diagnosed with AL amyloidosis are eligible for HSCT, although this number could increase with organ response after induction regimens with effective anti-cancer therapies (bortezomib and daratumumab).^135^^,^^139^^,^^140^

HSCT eligibility assessment is central to therapeutic success in this strategy. A single prospective randomized study compared HSCT with conventional chemotherapy, finding no overall survival benefit compared to a less intensive strategy. However, in this study the high mortality rate associated with HSCT (24%) was related to the inclusion criteria, the inexperience of the transplant center and the use of a subtherapeutic dose of melphalan.^141^

Since the literature provides no precise well-established prospectively validated HSCT eligibility criteria, each center establishes its own. Although subjective assessment is relevant, HSCT can be guided by certain factors: “physiological age” ≤ 70 years, creatinine clearance > 30 mL/min (except for patients on chronic dialysis), troponin T < 0.06 ng/mL, Eastern Cooperative Oncology Group performance scale ≤ 2, NYHA functional class I or II, and systolic blood pressure > 90 mmHg. Adequate pre-treatment risk stratification, concomitant with the development of better supportive conditions, such as antibiotic therapy and intensive care, has led to a reduction in HSCT-associated mortality in recent decades, with rates of 2.4% to 3.4% in retrospective analyses.^142^^–^^148^

Therefore, HSCT should be recommended as the first-line therapy in eligible patients (Table 12). This recommendation is due to the achievement of high hematological response rates, leading to reduced production and potential reabsorption of amyloid fibrils, with consequent improvement in organ dysfunction and performance status, as well as increased survival and quality of life. As an example, in a series of 672 AL-CA patients undergoing HSCT, 84% had a hematologic response, 39% of which were a complete response, and survival was > 50% at 15 years.^146^^,^^147^^,^^149^

An upfront HSCT can be performed soon after diagnosis in patients with < 10% clonal plasma cells in the bone marrow or when preceded by induction therapy with regimens containing bortezomib and/or anti-CD38 monoclonal antibody. The latter should be considered when there is an association with multiple myeloma, a predictable delay in HSCT, poor performance that may improve with induction therapy, and > 10% infiltration of the bone marrow by clonal plasma cells, which is associated with worse prognosis.^135^^,^^150^

The main regimens used as pre-transplant therapy are cyclophosphamide, bortezomib and dexamethasone (CyBorD) and daratumumab, bortezomib, cyclophosphamide and dexamethasone (Dara-CyBorD).^139^

Given that new therapies for treating AL amyloidosis are emerging and the scarcity of randomized studies on transplantation, further studies will be needed to determine whether HSCT will remain the most effective therapy in coming decades.

#### 8.1.2. Treating Patients Ineligible for HSCT/Conventional Chemotherapy

Most patients diagnosed with AL-CA are not eligible for higher intensity autologous HSCT therapy due to comorbidities, such as advanced heart disease, renal failure, the involvement of more than two organs, or advanced age. Thus, in this group of patients, chemotherapy with anti-cancer medications is the basis of treatment (Table 12). The regimens are similar to those for multiple myeloma.

The CyBorD regimen was found to be highly effective (81% to 94%), well tolerated, and capable of producing a rapid hematological response (within 3 months) in two retrospective studies with small samples (n = 17 and 43). Two-year overall survival reached 92% in these studies.^151^^,^^152^ The largest sample to have been tested with this regimen included 230 patients, 60% of whom had a hematologic response, and 23% of whom had a complete response. However, cardiac and renal organ responses were observed in only 17% and 25% of patients, respectively. A lower hematologic response rate was observed among patients with advanced heart disease (42% global and 14% complete), with a median overall survival of 7 months for this group^153^ Since the CyBorD regimen may make patients who are initially ineligible for autologous HSCT eligible (after hematologic response and clinical improvement), it should be considered when developing the therapeutic plan.^153^^–^^155^

Two recent randomized studies tested chemotherapy combinations as new treatments of choice in AL-CA patients who are ineligible for HSCT. The first compared a regimen of bortezomib, melphalan and dexamethasone to melphalan and dexamethasone, finding that adding bortezomib resulted in a higher overall hematologic response rate after 3 months (79% vs. 52%), a greater cardiac organ response (38% vs. 28%), and improved overall survival, with a 2-fold reduction in mortality.^156^ The largest randomized trial for AL-CA (ANDROMEDA) associated an anti-CD38 monoclonal antibody (daratumumab) with the CyBorD regimen, finding promising results.^139^ The Dara-CyBorD group had an overall hematologic response of 92%, compared to 77% in the CyBorD group, with an OR of 53% vs. 18%, which took a median of 2 months to achieve. Better organ function was also observed in the group that received the monoclonal antibody, with improved progression-free survival. Importantly, advanced heart disease patients (classified as stage IIIb) were excluded from both of these randomized studies, and treatment for this group remains a challenge in clinical practice.

### 8.2. ATTR-specific Therapies

Several steps in the pathophysiological process of amyloid fibril formation and deposition in cardiac tissue are potential therapeutic targets in ATTR-CA, as illustrated in Figure 12, including: 1) liver transplantation; 2) TTR tetramer stabilizers; 3) hepatic TTR synthesis inhibitors; and 4) the degradation and resorption of deposited amyloid fibrils.

**Figure 12:** The pathophysiological process of amyloid fibril deposition in ATTR and recognized therapeutic targets.

#### 8.2.1. Liver Transplantation

In the past, liver transplantation has been proposed as a treatment for patients with ATTRv-associated polyneuropathy.^157^ Liver transplantation, which removes the source of mutated TTR molecules, is associated with increased survival, with a reported 20-year survival rate of 55.3%. However, TTR deposition may continue after liver transplantation and is associated with heart disease progression, probably because the accumulated amyloid fibrils in the myocardium promote additional deposition of wild-type TTR over time.^158^^,^^159^ Thus, heart and liver transplantation may be possible and appears to be associated with better prognosis than the transplantation of either organ alone.^160^ Considering the reduced availability of organs and transplant centers, in addition to the risks posed by lifelong immunosuppression, the development of new therapies capable of blocking hepatic TTR synthesis should replace liver transplantation as a means of suppressing TTR production.

#### 8.2.2. Selective Transthyretin Kinetic Stabilizers

##### 8.2.2.1. Tafamidis

Tafamidis is a small molecule that selectively inhibits the dissociation of TTR tetramers by binding to thyroxine binding sites, thereby effectively inhibiting the cascade that results in amyloid fibril formation.^161^ When tested in a phase 3 clinical trial, tafamidis was effectively reduced the progression of neurological manifestations in patients with early-stage ATTRv polyneuropathy.^162^

Tafamidis is the only drug to have been specifically tested in CA patients in a prospective, randomized, placebo-controlled, multicenter clinical trial (ATTR-ACT).^163^ This phase 3 trial included 441 patients (18 to 90 years of age) diagnosed with hereditary or wild-type ATTR cardiomyopathy who were characterized by a history of heart failure, interventricular septal thickness > 12 mm on echocardiography, TTR amyloid deposits (confirmed by biopsy or positive bone marker scintigraphy), NT-Pro-BNP > 600 pg/mL, and > 100 meters walked in the 6-minute walk test. The main exclusion criteria were: NYHA functional class IV, AL-CA, and GFR < 25 mL/min/1.73 m^2^. The patients were randomized to receive tafamidis 80 mg/day, tafamidis 20 mg/day, or placebo in a 2:1:2 proportion. The study’s primary endpoint was hierarchically assessed all-cause mortality, followed by the frequency of cardiovascular hospitalization over 30 months of follow-up. The main secondary outcomes were change in 6-minute walk test results and quality of life scores according to the Kansas City Cardiomyopathy Questionnaire. The tafamidis 80 and 20 mg groups were merged for the statistical analysis, and the results indicated that these patients (n = 264) had a 30% lower relative risk of all-cause mortality (RR = 0.70 [95% CI: 0 .51-0.96]), 32% fewer cardiovascular hospitalizations (RR = 0.68 [95% CI: 0.56-0.81]), a lower rate of decline in 6-minute walk test results (p < 0.001), and a lower rate of decline in Kansas City Cardiomyopathy Questionnaire scores (p < 0.001) than the placebo group (n = 177). The Kaplan-Meier survival curves showed that tafamidis resulted in lower all-cause mortality, with the curves diverging after approximately 18 months of treatment, a result that agrees with the concept that tafamidis modifies the natural history of the disease. Tafamidis was well tolerated, with a similar incidence of adverse effects in the treatment and placebo groups. In the patient subgroup analysis, tafamidis was associated with lower all-cause mortality than placebo independently of NYHA functional class or ATTR genotype (hereditary or wild-type). However, patients in NYHA functional class III at the time of inclusion who were allocated to the tafamidis group had higher rates of hospitalization than the placebo group, a result likely explained by longer survival during a more severe disease phase. This subgroup analysis demonstrates the need for a trial with a large enough sample size to specifically assess the effect of tafamidis in ATTR patients with more advanced symptoms of heart failure.

More recently, an open-label extension study of ATTR-ACT found that 80 mg/day of tafamidis resulted in significantly higher survival than 20 mg/day (RR = 0.70 [95%CI: 0.50 - 0.979], p = 0.0374).^164^

Based on this evidence, tafamidis 80 mg/day is recommended for patients with ATTRv or ATTRwt, with NYHA I to III HF, without severe renal dysfunction, and who are beginning therapy at the earliest stages of the disease (Table 12). In Brazil, tafamidis 80 mg/day was approved by the Brazilian Health Regulatory Agency for treating ATTR-CA.

**Table 12:** Recommendations for cardiac amyloidosis treatment

Recommendation	Recommendation class	Level of evidence (references)
Recommendations for heart failure treatment		
Loop diuretics to control congestive manifestations	I	C
Avoid substances that cause bradycardia, except in special situations	I	C
Routine use of HFrEF drugs: beta-blockers, ACEi, ARB, ARNi, SGLT2i, MRA	III	C
Oral anticoagulation for patients with CA and atrial fibrillation, regardless of the calculated risk of stroke or systemic embolism	I	C
For patients with a confirmed diagnosis of CA, offer referral to a specialized center	I	C
Recommendations for specific ATTR-CA treatment		
Tafamidis 80 mg/day for ATTRv or ATTRwt CA with heart failure (NYHA FC I to III) and no severe renal dysfunction in order to reduce mortality, disability progression, and quality-of-life loss	I	B (ref. 162, 163)
Recommendations for specific AL-CA treatment		
Joint assessment with hematologist in suspected or confirmed AL-CA cases to determine treatment:		
Consider HSCT for eligible AL-CA patients, ie, no severe comorbidities, advanced heart disease, or severe renal failure, having no more than two affected organs, and not of advanced age	I	B (ref. 147, 148, 149)
Initiate chemotherapy with anti-cancer medications in AL-CA patients who are ineligible HSCT	I	A (ref. 139,156)

*ACEi: angiotensin converting enzyme inhibitor; ARNi Angiotensin–neprilysin inhibitor; ARB: angiotensin-II receptor blocker; CA: cardiac amyloidosis; FC: functional class; HSCT: hematopoietic stem cell transplantation; MRA: mineralocorticoid receptor antagonist.; NYHA: New York Heart Association; SGLT2i: sodium-glucose cotransporter 2 inhibitor*

##### 8.2.2.2. AG10

AG10 is a selective TTR tetramer stabilizer designed to mimic the structural influence of a super-stabilizing mutation (T119M), which significantly reduces the dissociation rate of tetramers.^165^ This agent was evaluated in a multicenter, phase 2, randomized, double-blind, placebo-controlled trial that included 49 patients with symptomatic ATTR cardiomyopathy and NYHA functional class II or III. The treatment was well tolerated and resulted in almost complete TTR stabilization.^166^ There is an ongoing phase 3 multicenter clinical trial testing the effects of AG10 in CA patients. (ClinicalTrial.gov – Identifier: NCT03860935).

#### 8.2.3. Inhibitors of Hepatic TTR Synthesis

Therapies based on silencing the expression of genes that encode hepatic TTR production are very promising, including RNA interference (patisiran) and antisense oligonucleotide (inotersen) strategies. Both drugs have been tested in multicenter phase 3 trials in patients with ATTRv polyneuropathy and were shown to be effective in reducing the progression of neurological manifestations.^167^^,^^168^ Post-hoc analyses of subgroups of CA patients in these studies suggest positive effects on the progression of cardiomyopathy. Both classes of gene expression silencers are currently being tested in multicenter phase 3 trials in ATTR-CA patients (ClinicalTrials.gov Identifiers: NCT03997383 and NCT04136171).

#### 8.2.4. Degradation and Resorption of Amyloid Fibrils

In vitro and experimental studies have shown that certain compounds based on hydrophobic molecules promote the degradation of amyloid tissue deposits, allowing their resorption through the macrophage system.^169^ Experimental studies have found that doxycycline, an antibiotic of the tetracycline family, is one such compound,^170^ having synergistic effects with tauroursodeoxycholic acid.^171^ Although promising in preclinical studies, clinical experience with this approach is limited and has not established its efficacy or provided recommendations for its use.

## 9. Heart Failure Syndrome Management

In addition to specific therapy for amyloidosis, supportive treatment for HF may be necessary. CA initially presents as HFpEF and a restrictive pattern of LV filling, which could lead to disease progression and reduced EF. This pathophysiological mechanism could explain the difficulties in clinical management of CA patients when using HFrEF medications.^20^^,^^21^

Maintaining euvolemia through water restriction and medications is the focus. Loop diuretics, the most frequently used medication, are indicated to reduce pulmonary and systemic congestion, and they may be associated with aldosterone antagonists (Table 12). Using diuretics to achieve euvolemia can be a challenge, since excessive dosage can impair renal function and/or result in low cardiac output due to reduced preload in hearts with already reduced stroke volume.^172^^,^^173^ Furthermore, in patients with autonomic polyneuropathy, hypotension may impede diuretic use due to unstable preload conditions.^172^

Regarding HFrEF drugs, there is no scientific evidence that neurohormonal antagonists such as angiotensin II-converting enzyme inhibitors, angiotensin II receptor blockers, and beta blockers, or even substances recently described as neprilysin inhibitors, angiotensin II receptor antagonists, and SGLT2 inhibitors, have any effect on CA, and they also involve the risk of hypotension and autonomic dysfunction. In a retrospective, single-center study of 99 CA patients (33% AL-CA and 67% ATTR-CA), Aimo et al.^173^ observed that an association of angiotensin II-converting enzyme inhibitors/angiotensin II receptor blockers and mineralocorticoid antagonists can be used safely and with gradual dose adjustments if there are no contraindications, as well as that tolerance for beta-blockers is lower in AL-CA patients with left or right ventricular dysfunction. Beta-blockers and non-dihydropyridine calcium channel blockers are usually not well tolerated, because, due to the low ejected systolic volume, cardiac output is maintained through increased heart rate. In addition, non-dihydropyridine calcium channel blockers should be avoided in in AL-CA patients, since they bind to amyloid fibrils, which can result in advanced blocks and cardiogenic shock.^172^^,^^173^

### 9.1. Arrhythmia Management

Arrhythmias are very common in CA patients and are usually symptomatic and poorly tolerated. Arrhythmia assessment in this population should involve three different situations: atrial arrhythmias, ventricular arrhythmias, and conduction system disease.

#### 9.1.1. Atrial Arrhythmia and Anticoagulants

Amyloid deposition leads to atrial thickening, including changes in atrial relaxation and increased intracavitary pressure, which produces atrial dilatation that, in association with atrial fibrosis, predisposes patients to atrial fibrillation (AF) or other atrial arrhythmias. The prevalence of AF in CA patients can range from 11% to 71%, being even higher in ATTR-CA, possibly because it affects older men.^174^^,^^175^

Managing AF in these patients is generally difficult, since they usually cannot tolerate drugs such as beta-blockers, non-dihydropyridine calcium channel blockers or digitalis due to the fact that they cause postural hypotension and HF decompensation. Given the need for these medications, it is advisable to use low doses with careful hemodynamic monitoring, in addition to serum level control, if digoxin is used. Regarding rhythm control, a retrospective analysis found no difference in survival between patients who received antiarrhythmic drugs and those treated with frequency control alone.^176^ Recent data suggest that catheter ablation may be associated with lower mortality in CA patients, especially when performed early. However, these data are from small retrospective observational studies that also had a high rate of AF recurrence.^177^

Finally, the reduced contractility caused by amyloid infiltration in atrial tissue may also contribute to thrombus formation. Autopsy studies have found that up to 33% CA patients have intracavitary thrombi,^178^ while retrospective studies have found a prevalence of 15 to 33%.^179^^,^^180^Therefore, anticoagulation is indicated in CA patients who develop AF, regardless of risk score calculations (Table 12). In addition, left atrial thrombus has been described in up to 30% of patients who underwent transesophageal echocardiography prior to a planned electrical cardioversion, even with adequate anticoagulation.^181^^,^^182^ Thus, a transesophageal echocardiogram is recommended for every electrical cardioversion candidate. The role of anticoagulation in patients in sinus rhythm is still uncertain. However, even in sinus rhythm, changes in atrial contractility are common and are associated with atrial thrombus formation, especially in AL-CA patients.^180^^,^^182^

#### 9.1.2. Ventricular Arrhythmias

Ventricular arrhythmias are frequent in CA patients, especially AL-CA. Previous studies have detected complex ventricular arrhythmias in at least 50% of AL-CA patients, in whom non-sustained ventricular tachycardia was the most frequent arrhythmia and was associated with lower survival.^183^^,^^184^

In this context, implantable cardioverter-defibrillators (ICD) may have a role in preventing sudden death in CA patients. ICD may benefit patients with unstable ventricular tachycardia or who have survived cardiac arrest without a reversible cause and a life expectancy > 1 year with significant quality.^185^^–^^187^

However, recommending ICD as primary prevention is difficult for a number of reasons. The first is that most causes of sudden death are related to electromechanical dissociation and not ventricular arrhythmias.^188^ The second reason is the historically low life expectancy of CA patients, especially those with AL-CA. Finally, traditional risk stratification tools, such as reduced EF, do not really seem applicable to CA patients, since severe systolic dysfunction is associated with the late stages of the disease, in which pump failure predominates as the cause of death. The challenge is, therefore, to identify at-risk patients in the early stage of the disease when arrhythmia predominates and can potentially be corrected with ICD. Prospective studies are necessary to determine when ICD can be beneficial in CA patients.^189^

### 9.2. Conduction Disorders

Conduction-system disease is highly prevalent among CA patients, with atrioventricular conduction being more commonly affected than the sinus node. The involved pathophysiology has not yet been fully defined, although small studies have suggested that the amyloid protein conduction system is involved.^190^ There is evidence that these changes are the cause of death in a considerable number of CA patients,^191^for whom pacemakers are often indicated, especially ATTR-CA patients.^178^In a Columbian cohort, a pacemaker was indicated in 43% of ATTRwt patients and 36% of ATTRv patients.^192^ Pacemaker use in this population should follow traditional implantation recommendations.^193^

### 9.3. Therapeutic Options in Advanced Heart Failure

In advanced HF associated with CA, advanced support strategies, such as mechanical circulatory assistance and transplantation, present challenges, especially since it is a multisystem disease. Moreover, due to the reduced size of the left ventricular cavity and the frequent involvement of the right ventricle, the use of long-term mechanical circulatory assistance devices may be limited.^193^^,^^194^ Although heart transplantation has historically had a lower survival curve in CA,^191^the most recent results indicate that it is similar to other etiologies.^195^ This change is related to better patient selection and specific strategies, such as double transplantation for ATTRv patients and heart transplantation prior to bone marrow transplantation in AL-CA.^196^

Table 12 summarizes the recommendations for CA treatment.

## 10. Centers of Excellence/Reference and New Treatment Remuneration Models

Access to care and a sustained commitment to improving care quality are fundamental for achieving excellent results, which are critical for complex diseases such as CA. Developing a course of care that provides the right care at the right time for CA requires the involvement of public and private health care managers, health professionals, and everyone involved in the care of these patients.

CA, particularly AL-CA, must be approached with a view to rapid diagnosis, and multidisciplinary teams with proven experience and clinical protocols based on the best scientific evidence must be available. In addition, comprehensive care and the monitoring and publishing of care results should be promoted. An integrated and collaborative practice model should be observed at these reference centers, with a telemedicine structure to support diagnosis at more distant centers, patient monitoring, and clinical research, which is the basis for emerging therapies. Such initiatives have been undertaken at a number of centers worldwide, including some in Brazil.^187^^–^^199^

Additionally, such reference centers should contribute to a National Registry of Cardiac Amyloidosis, allowing better insight into regional epidemiology and care quality, in addition to measuring patient-centered clinical outcomes and contributing to new public policies.

New financing models for high-cost rare disease treatments have been discussed, tested in pharmacoeconomic studies, and implemented around the world.^200^ A risk-sharing strategy for pharmaceutical companies, funders, and healthcare providers must involve clinical research, being based on outcome assessment and the impact of clinical protocols. This new paradigm is under development in Brazil for rare diseases, and it can be used for CA.^201^^,^^202^ Ensuring the sustainability of the health system and access to excellent treatment for CA patients will increasingly involve the Brazilian Society of Cardiology and its scientific departments/study groups. The Society is promoting debate on good care practices in all of these critical dimensions in order to build a health system that focuses on patient needs and fights waste.

An assessment of new treatments for rare diseases by the National Commission for Technological Incorporation in the Unified Health System (CONITEC) indicated that approximately 52% of the evaluated medicines have already been incorporated. In our vision were are building a new, technologically-based scenario developed through robust data with the support of clinical protocols and reference centers that can replace the current model, where access is sought through judicialization, which has a heavy impact on the federal budget.^197^

## 11. Knowledge Gaps and Future Perspectives

Despite recent advances in CA research, there are still numerous knowledge gaps to be filled regarding this complex and multifaceted disease. Both the diagnosis and treatment of amyloidosis are evolving, which is exemplified by the 638 studies registered in the clinicaltrials.gov site that have recently been completed or are in progress.

Current cardiovascular imaging for CA diagnosis involves cardiac scintigraphy with bone-seeking radiotracers, such as ^99m^Tc-pyrophosphate, which allows molecular images of amyloid fibril deposition in the myocardium. Although not yet available in Brazil, another promising technique is PET imaging with ^18^F-Florbetapir in peripheral nerves and/or other extracardiac sites.^203^^,^^204^Elastography, ultrasound, and CMR are also being used to assess the degree of myocardial fibrosis and may be additional and promising tools to aid in diagnosis and prognosis.

Recent studies have focused on transcriptome analysis, seeking differences in expression between healthy and sick individuals by means of molecular analysis and integrative genomics. The transcriptome of AL-CA patients is similar to that of patients with monoclonal gammopathy of undetermined significance. Furthermore, the level of circulating microRNA, which is known to correlate with cardiac damage, is increased in AL patients. Through principal component analysis, highly overlapping phenotypic profiles have been found between AL, monoclonal gammopathy of undetermined significance, and multiple myeloma.^205^^,^^206^

Additionally, using artificial intelligence to analyze data in medical databases is a promising strategy for identifying individuals with warning signs. This strategy could lead to earlier CA diagnosis and reduce treatment delay.

New therapies specifically for ATTR-CA are under intense investigation. TTR gene silencing therapies that have been found effective for hereditary amyloid polyneuropathy are currently being tested in ATTR-CA patients in large multicenter studies. The APOLLO-B study is testing the RNA interference agent patisiran (ClinicalTrials.gov Identifier: NCT03997383). The CARDIO-TTRansform clinical trial is testing antisense oligonucleotide technology in a new second-generation drug, AKCEA-TTR-LRx (ClinicalTrials.gov Identifier: NCT04136171).

Since amyloid deposition has arrhythmogenic potential and can damage the conduction system, implantable devices such as pacemakers or defibrillators are frequently used to reduce mortality and increase survival in this population. On the other hand, individuals with CA and atrial fibrillation or flutter are at high risk for cardioembolic events, and treatment with anticoagulants has been recommended. Although there is a pathophysiological rationale for using these interventions in CA patients, they must still be tested in appropriate clinical trials, and this is an important area for future clinical investigation. In addition, heart transplantation has proven to be a safe strategy in these patients, although artificial ventricles and combined therapies must still be assessed in clinical trials.

To define the best treatment options and combinations for this disease, we must wait for the results of studies that are testing different interventions and specific therapies, bearing in mind that: 1) we are not fully aware of the details of the disease’s pathophysiology; 2) we lack a broad understanding of how medications work in this disease; 3) we still do not have an accurate, detailed and long-term assessment of the risks and benefits of different treatments; 4) and we also do not know the dose-response relationship of different medications for this clinical scenario.

Thus, we believe that the following basic steps are relevant and should be implemented:

1. Creating new centers of reference/excellence in CA.
2. Training professionals in early CA recognition and referral to specialized centers.
3. Promoting the development of a National Registry of Cardiac Amyloidosis.
4. Discussing the safety and care quality challenges involved in the journey of CA patients.
5. Discussing new remuneration and care models for CA.
6. Encouraging clinical research on CA in Brazil.
